# Human-in-the-Loop Optimization of Transcranial Electrical Stimulation at the Point of Care: A Computational Perspective

**DOI:** 10.3390/brainsci12101294

**Published:** 2022-09-26

**Authors:** Yashika Arora, Anirban Dutta

**Affiliations:** 1 Neuroimaging and Neurospectroscopy Lab, National Brain Research Centre, Gurgaon 122052, India; 2 Neuroengineering and Informatics for Rehabilitation and Simulation-Based Learning (NIRSlearn), University of Lincoln, Lincoln LN6 7TS, UK

**Keywords:** systems analysis, model predictive control, transcranial electrical stimulation, functional near-infrared spectroscopy, pupillometry

## Abstract

Individual differences in the responsiveness of the brain to transcranial electrical stimulation (tES) are increasingly demonstrated by the large variability in the effects of tES. Anatomically detailed computational brain models have been developed to address this variability; however, static brain models are not “realistic” in accounting for the dynamic state of the brain. Therefore, human-in-the-loop optimization at the point of care is proposed in this perspective article based on systems analysis of the neurovascular effects of tES. First, modal analysis was conducted using a physiologically detailed neurovascular model that found stable modes in the 0 Hz to 0.05 Hz range for the pathway for vessel response through the smooth muscle cells, measured with functional near-infrared spectroscopy (fNIRS). During tES, the transient sensations can have arousal effects on the hemodynamics, so we present a healthy case series for black-box modeling of fNIRS–pupillometry of short-duration tDCS effects. The block exogeneity test rejected the claim that tDCS is not a one-step Granger cause of the fNIRS total hemoglobin changes (HbT) and pupil dilation changes (*p* < 0.05). Moreover, grey-box modeling using fNIRS of the tDCS effects in chronic stroke showed the HbT response to be significantly different (paired-samples *t*-test, *p* < 0.05) between the ipsilesional and contralesional hemispheres for primary motor cortex tDCS and cerebellar tDCS, which was subserved by the smooth muscle cells. Here, our opinion is that various physiological pathways subserving the effects of tES can lead to state–trait variability, which can be challenging for clinical translation. Therefore, we conducted a case study on human-in-the-loop optimization using our reduced-dimensions model and a stochastic, derivative-free covariance matrix adaptation evolution strategy. We conclude from our computational analysis that human-in-the-loop optimization of the effects of tES at the point of care merits investigation in future studies for reducing inter-subject and intra-subject variability in neuromodulation.

## 1. Introduction

Grey-box modeling of the signals from brain–computer interfaces—viz., portable brain imaging and pupillometry—can provide causal inference of the impairments in neurological patients [1,2,3], such as those with Alzheimer’s disease and Alzheimer’s-disease-related dementias [4]. Specifically, evoked brain responses in a multimodal brain–computer interface—when combined with cognitive, motor, or transcranial electrical stimulation (tES)—can enable system analysis and design of therapeutic interventions with human-in-the-loop optimization [5] using brain-based metrics. Then, multimodal brain imaging can cross-validate the metrics across different physiological domains. For example, simultaneous functional near-infrared spectroscopy (fNIRS) and electroencephalography (EEG) can elucidate neurovascular modulation by tES in health and disease [6], which can be dysfunctional post-stroke [7]. Here, transient coupling relationships between the changes in EEG power spectra and fNIRS hemodynamics signals during tES can be monitored using Kalman-filter-based online parameter estimation of an autoregressive (ARX) model [8]. Then, a grey-box modeling approach [9] can provide physiological insights based on a detailed multi-compartmental neurovascular model incorporating vascular smooth muscle, perivascular space, synaptic space, and astrocyte glial cells. Such computational modeling, when fitted to individual dysfunction, can allow personalized therapeutic interventions, e.g., tES with model predictive control using subject-specific fNIRS-based measures of tES evoked blood volume changes (e.g., total hemoglobin concentration changes)—called cerebrovascular reactivity (CVR) to tES. However, for model predictive control, computationally expensive and physiologically detailed nonlinear models need to be simplified with an appropriate complexity (e.g., based on Akaike information criterion) adequate for online “real-time” performance. Here, the simplified model needs to capture the inherent dynamic characteristics of the neurovascular system in the form of natural frequencies and damping factors, e.g., the changes in the dynamic characteristics from the arterioles as they transition into cerebral capillaries, with smooth muscle cells replaced by pericytes and mural cells embedded into the endothelial basement membrane in the smaller vessels. Mode decomposition approaches [7,10], including dynamic mode decomposition [11], can be applied to reduce the complexity of the multimodal multichannel data to their dominant features and essential components. Here, experimental data from multi-distance fNIRS probes can capture a combination of vessel oscillations from the pial, penetrating, and precapillary arterioles to the capillaries, based on the inter-optode distance and photon migration through the neurovascular tissue determined by the fNIRS optode’s point-spread functions. Moreover, cerebrospinal fluid (CSF) pulsatility in the subarachnoid space due to pial vessels can be picked up by the near-infrared backscattering using shorter-distance probes; however, delineating different “dynamic modes” in the fNIRS signal vis-à-vis a mechanistic understanding of subject-specific dysfunction would require a physiologically realistic computational model parametrized with the inherent dynamic characteristics of the neurovascular system. Various physiologically relevant frequency bands have already been identified in the literature [12], e.g., 0.6–2 Hz and 0.145–0.6 Hz are related to cardiac and respiratory function, 0.052–0.145 Hz is associated with smooth muscle cell activity, and 0.021–0.052 Hz has been proposed to reflect smooth muscle autonomic innervation [12]. Norepinephrine [13] receptors are present on the pial arterial smooth muscle cells [14,15]. In fact, vasomotion can be elicited via a contractile stimulation of single-dose norepinephrine in internal thoracic artery segments [16]. Such evoked responses can provide biomarkers, e.g., vasomotion is associated with endothelial dysfunction [16], while norepinephrine deficiency has been linked to the pathogenesis of Alzheimer’s disease (AD), which can be related to reduced vessel pulsatility and amyloid-beta clearance [17] via the perivascular pathways [18]. Additionally, 0.01–0.02 Hz oscillations are known to be crucial for supporting higher oxygen concentrations distant from the small vessels [19]. Zhao et al. [20] found a drop in the oscillatory power in the 0.01–0.02 Hz frequency band during Mini-Cog assessment for dementia, where this drop was more significant in type 2 diabetes mellitus (T2DM) patients than in age-matched normoglycemic elderly controls. Small-vessel oscillations support nutrient supply, where low-frequency Fahraeus–Lindqvist-driven (not blood-pressure-driven) oscillations in the small vessels are crucial [19]. Mechanistic understanding of the causes of diabetic brain fog may be found in terms of the small-vessel pulsatility in the 0.01–0.02 Hz frequency band and the oxygen diffusion (including oxygen extraction fraction) distant from the small vessels [19]. Here, modal analysis can provide the characteristic dynamics of a detailed, multi-compartmental neurovascular system from its natural frequencies, mode shapes, and damping factors and develop a simpler mathematical model of the system’s behavior for therapeutic intervention with tES. Typically, modal analysis methods are prevalently used in structural and fluid mechanics and can be well applied for biomedical system analysis to derive the modal behavior of the output responses.

**Figure 1:**
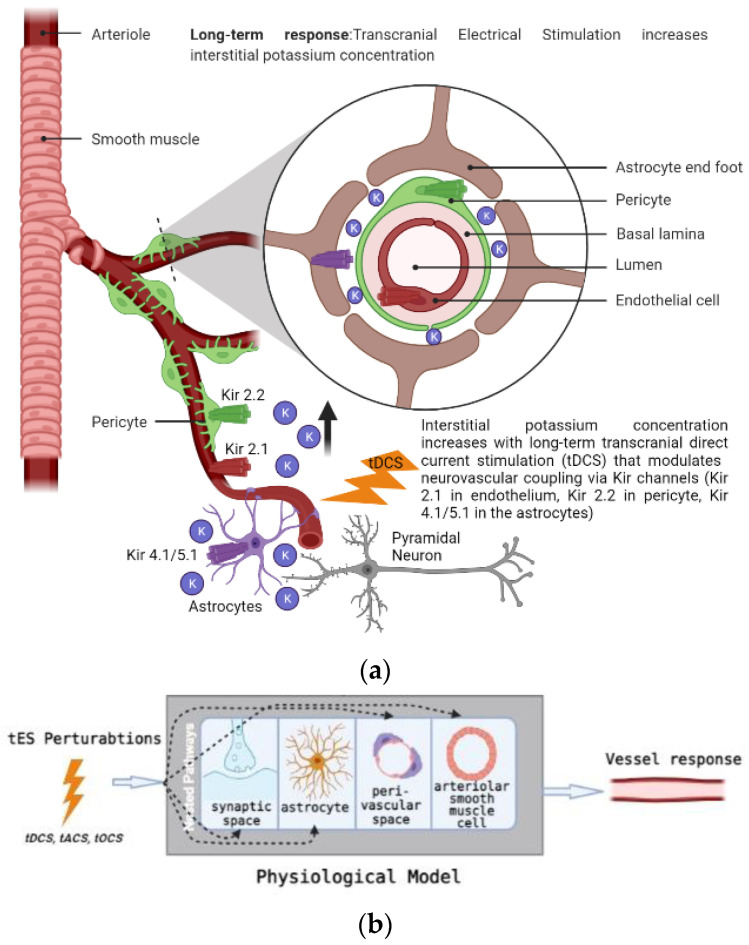
(**a**) Long-term (≥3 min) transcranial electrical stimulation can change the interstitial concentration of potassium, modulating the neurovascular system’s sensitivity via Kir channels. (**b**) Four-compartment lumped physiological model of the neurovascular unit with nested pathways (dashed arrows) that can be perturbed by the tES current density, leading to vessel response in terms of diameter changes.

Investigation of different “dynamic modes” in the vessel oscillations in health and disease is also crucial, since vascular factors are an essential contributor to cerebrovascular disease [21], including a role in mild cognitive impairment and dementia [22], which is predicted to increase to 152 million by 2050 (Alzheimer’s Disease International London, UK, 2019). Here, we postulate that tES-evoked “dynamic modes” [7] may be more informative than the resting-state ones, and that tES-evoked onset vascular response is partially driven by the arousal effect via the locus coeruleus norepinephrine (LC-NE) system. Transcranial direct-current stimulation (tDCS)—a tES modality—can perturb the blood vessels and evoke regional cerebral blood flow (CBF) [23]. We found that the CVR to tDCS following a cerebrovascular accident was significantly less in the lesioned hemisphere [24], which was postulated to be related to neurovascular coupling status. Since stroke is a specific vascular risk factor for dementia [25], the neurovascular coupling (and oxygen extraction fraction)_status may be relevant [26] for dosing tES. Then, anodal tDCS-induced neuronal excitation may cause an energetic depletion that can be quantified and validated by ^31^phosphorus magnetic resonance spectroscopy [27] for tES dosing. Here, tDCS-induced cerebral energy consumption has been shown to promote systemic glucose tolerance in a standardized euglycemic–hyperinsulinemic glucose clamp procedure in healthy male volunteers. In fact, the effects of tDCS may be similar to the cognitive-load-led reduction in blood glucose [28], where stressor-related norepinephrine release and regulation of astrocytic and neuronal metabolism are relevant [29]. The LC-NE network is known to optimize coupling of cerebral blood volume with oxygen demand [30], which can affect the neurovascular coupling [6,8]. Thus, the vasoconstricting perivascular pathway via norepinephrine [13] receptors on the pial arterial smooth muscle cells [14,15] may be relevant at the onset (<150 s after tDCS) of tDCS [9]. Then, longer-duration (>150 s) tDCS can modulate the neurovascular coupling [9], cerebral oxygenation, and synaptic potentiation, likely via postsynaptic signaling, including nitric oxide and interstitial potassium concentration, as shown in Figure 1a.

Smooth muscle autonomic innervation can regulate vascular tone [12]. The autonomic nervous system (ANS) comprises the sympathetic and parasympathetic nervous systems. ANS neurotransmitters include norepinephrine (NE), adenosine triphosphate (ATP), and neuropeptide Y (NPY), which function as vasoconstrictors, whereas acetylcholine (Ach) and calcitonin gene-related peptide (CGRP) can mediate vasodilation. Immediate -onset effects of tDCS on the blood volume changes (e.g., total hemoglobin concentration changes), including the “initial dip” [7], may be subserved by the evoked (im)balance of autonomic effectors, including NE and Ach. Here, direct electrical stimulation of noradrenergic axons is possible [13], in addition to arousal effects via the LC-NE system. Since prior works have found coupling between the alpha-band (8–12 Hz) EEG activity and pupil diameter [31], as well as between log (base-10)-transformed EEG band power (0.5–11.25 Hz) and fNIRS signals [8], we aimed to investigate the coupling between the pupil dilation and fNIRS signals during tDCS. Specifically, the Granger causality test was used to assess whether tDCS waveforms in a 3D vector autoregression (VAR) model would Granger-cause total hemoglobin changes (i.e., blood volume changes) conditioned on the pupil dilation. This arousal effect is important to investigate mechanistically, since the peripheral effects of tDCS on brain circuits involving memory via the ascending fibers of the occipital nerve to the locus coeruleus [32]—e.g., during cerebellar tDCS [33]—can be beneficial in ameliorating cognitive impairment. Adrenergic regulation may also be relevant [29] in longer-duration (20 min) tDCS, which can lead to Ca^2+^ elevations in the astrocytes and a neurometabolic biphasic effect on systemic glucose tolerance [27]. Since pupil dilation is a correlate of Ach and LC-NE activity [34] during arousal, pupillometry with portable brain imaging and “short-duration (<150 s)” tES may elucidate the autonomic effectors vis-à-vis CBF [6].

CBF is known to be regulated primarily by three mechanisms: cerebral autoregulation, which maintains the CBF under changes in systemic blood pressure; cerebral vasoreactivity, which is the response to the arterial CO_2_ partial pressure changes; and neurometabolism, which is the response to the neuronal activity [35]. A recent study [36] showed that the spatial distribution of CBF changes was correlated with the tDCS-induced electric field distribution (<1 V/m) computed using finite element modeling. CBF changes can also be evoked rapidly (<100 ms) via transcranial alternating-current stimulation (tACS) at 10–20 Hz, albeit at a higher electric field strength (5–20 V/m) [37]. Here, tACS can target neural oscillations [38]; however, rapid changes in the CBF indicate a direct effect of the electric field on the vascular neural network [39]. Brain capillaries act as a neural-activity-sensing network that can be perturbed by tES to identify characteristic natural frequencies and damping factors from the resulting dynamics of the metabolic and vascular responses. A multiscale model is needed for mechanistic understanding of the metabolic responses, e.g., the computational model by Jolivet et al. [40], which captured the concentrations of lactate in neuronal, astrocytic, and extracellular compartments that were coupled as a modulatory feedback [41,42] to neuronal membrane voltage. Then, individual hemodynamic effects—including neurovascular network resonant frequencies—of the tES via various neurovascular pathways need to be investigated using a mechanistic-model-based hypothesis testing that is postulated to be subject-specific [43]. Such model-based neurometabolic system analysis is important, since mechanistic models can enable human-in-the-loop optimization of tES to enhance its metabolic effects for therapeutic applications, e.g., in T2DM.

Lumped vessel biomechanics play an important role in vessel oscillations [9], where CBF can be partially modulated by the balance of autonomic effectors on the vascular tone. However, more detailed grey-box modeling and analysis of the neurovascular coupling system need to include multiscale vessel biomechanics, where small vessels will have different characteristic natural frequencies than the pial vessels. Proximal pial arteries and the descending arteries have the fastest onset time, followed by the capillaries (the spatiotemporal characteristics of pial, penetrating, and micro-vessels are summarized in the work of Schmid et al. [44]), which can have several modes of oscillations, with frequencies ranging from 0.005 to 1 Hz. However, the oscillatory responses can be quite complex due to the interdependence of the nested spatiotemporal dynamics of the pial arteries, descending arteries, and capillaries. Moreover, various tES modalities show differences in the temporal profile of the electric current stimulation, which may perturb the vessel oscillations differently. In transcranial direct-current stimulation (tDCS), the current profile has a monophasic, non-oscillating constant value. In contrast, in transcranial alternating-current stimulation (tACS), the oscillating current reverses rhythmically at a specific frequency. Thus, tACS differs from tDCS in that it provides a mechanism for manipulating intrinsic oscillations through the injection of sinusoidal currents. The other methods are transcranial oscillating-current stimulation (tOCS), which uses tDCS to set a baseline to the tACS oscillations, and transcranial random noise stimulation (tRNS), which injects a “noisy” current with bounded stochasticity. Because tES’s modulatory effects on blood vessels can be mediated by the neuronal and non-neuronal cells in the neurovascular tissue, a deeper understanding of the signaling pathways is crucial for human-in-the-loop optimization of the effects of tES, including its effects on the vessel oscillations [45]. In this study, human-in-the-loop optimization was performed using a stochastic, derivative-free covariance matrix adaptation evolution strategy (CMA-ES) [46] that can be used for nonlinear, non-convex optimization problems with noisy measurements (https://cma-es.github.io/, accessed on 17 September 2020).

The following sections of this computational perspective article provide systems analysis of the tES effects that are relevant for model-based human-in-the-loop optimization. Section 2 presents the modal analysis of our published physiologically detailed neurovascular model [9] to elucidate the oscillatory responsiveness of the vasculature, including to cardiac and respiratory rhythms. Then, Section 3 presents grey-box modeling of the fNIRS of tDCS’s effects in a chronic stroke case series to elucidate the role of diseased states on the neurovascular system. Here, an initial dip in blood volume or vasoconstriction following tDCS perturbation was found in chronic stroke, necessitating the study of the role of tDCS-evoked arousal in health and disease. In Section 4, we performed black-box modeling of prefrontal fNIRS–pupillometry of short-duration frontal tDCS effects to elucidate the effects of tDCS-evoked arousal on hemodynamics in healthy individuals. Here, the variability in the effects of tES even in healthy individuals—possibly state–trait variability that can be challenging for clinical translation—motivated feasibility testing of human-in-the-loop optimization for a reduced-dimensions model (eight poles, two zeros [9]) for the predictive control of tES-evoked HbT in a healthy individual, as presented in the Section 5.

**Figure 2:**
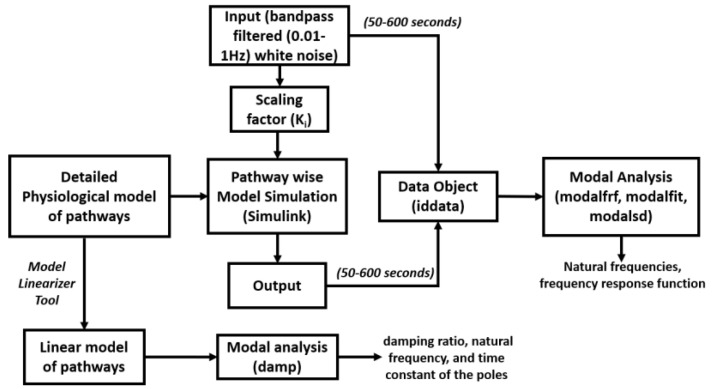
Modal analysis approach used for evaluating the physiological model using MATLAB and Simulink (MathWorks Inc., Natick, MA, USA).

In Section 2, in order to estimate the mechanistic aspects of the effects of tES, we used a mathematical model based on the neurovascular tissue physiology of the vascular response through various pathways that are susceptible to electric fields, as shown in Figure 1. The simulation model included four compartments based on published literature, where the tES current density perturbed synaptic potassium released from active neurons for Pathway 1, astrocytic transmembrane current for Pathway 2, perivascular potassium concentration for Pathway 3, and voltage-gated ion channel current on the SMC for Pathway 4. The implementation of the detailed model is presented in the work of Yashika et al. [9]. The physiologically detailed models were simulated using the “ode23tb” solver in Simulink (MathWorks, Inc., Natick, MA, USA). Prior work showed that the models captured the interactions between the potassium dynamics and the calcium dynamics in the perivascular space [6]. In this computational perspective article, we performed modal analysis based on the workflow shown in Figure 2, using the MATLAB and Simulink packages (MathWorks, Inc., Natick, MA, USA).

## 2. Modal Analysis of the Physiologically Detailed Neurovascular Model

In this study, we used a modal analysis approach to analyze the physiologically detailed neurovascular unit (NVU) model for evaluation of the oscillatory modes that may be perturbed by tACS. The physiological model considered the lumped neurovascular system of vascular smooth muscle (SMC) space, perivascular space, synaptic space, and astrocyte space; and captured the tES-induced direct and indirect vascular responses. The detailed physiological NVU model has been shown to simulate vessel oscillations in the range of 0.05–0.2 Hz governed by the interactions between the Kir 2.1 channels on the endothelium and the Kir 2.2 channels on the pericytes [6,47]. For modal analysis, we applied 10 tES perturbations—which were bandpass-filtered (0.01–1 Hz) white noise inputs of 600 s—to the four physiologically constrained NVU pathways, as shown in Figure 2 (equations are presented in the Supplementary Materials of Yashika et al. [9]). The input and output time series were stored using a time-domain data object (“iddata” in MATLAB, MathWorks, Inc., Natick, MA, USA). We excluded the initial 50 s of the transient response in the time-series data for modal analysis. We used the modal analysis functions “modalfrf” to determine frequency-response functions for modal analysis, “modalfit” to determine modal parameters from the frequency-response functions, and “modalsd” to generate a stabilization diagram for modal analysis of the data object in MATLAB (MathWorks, Inc., Natick, MA, USA). First, the frequency-response functions for the four tES pathways of the NVU system were found using “modalfrf” for a sample rate of 10 samples per second (10 Hz), where the noise was assumed to be uncorrelated with the input signals. Then, the natural frequencies of the four tES pathways for the NVU system were found from the frequency response using the “peak-picking” method (a fast and straightforward procedure for identifying peaks in the frequency-response functions) available in the “modalfit” function in the physiological frequency range of 0.01–0.2 Hz. Then, a single set of modal parameters was generated using the least-squares complex exponential (LSCE) algorithm by analyzing multiple response signals simultaneously in “modalsd”. Here, a stabilization diagram was used to identify the physical modes by examining the stability of the poles as the number of modes increased. Then, the linear model of the four physiologically detailed tES pathways in the NVU was found using the Model Linearizer tool in the Simulink (MathWorks, Inc., Natick, MA, USA) linear analysis package. The damping ratio, the natural frequency, and the time constant of the poles were obtained using the “damp” function from the linear model system.

**Figure 3:**
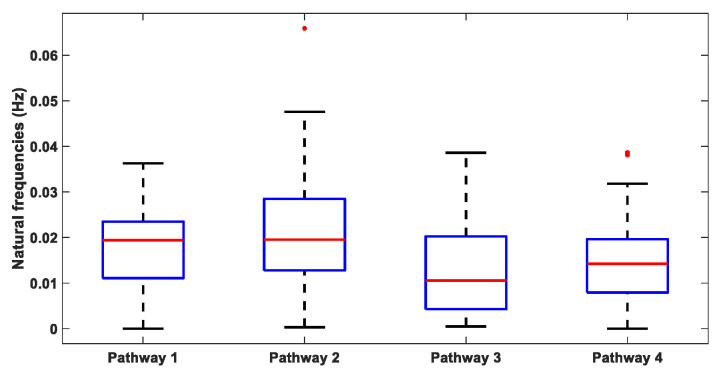
Boxplot of the natural frequencies in the physiological frequency range of 0.01–0.2 Hz obtained through modal analysis for the four tES perturbation model pathways. On each box, the central mark indicates the median, and the bottom and top edges of the box indicate the 25th and 75th percentiles, respectively. The whiskers extend to the most extreme data points not considered outliers, and the outliers are plotted individually using the red “+” symbol.

Table S1 (Supplementary Materials: Modal analysis of the physiologically detailed neurovascular model) lists the natural frequencies in the physiological frequency range of 0.01–0.2 Hz obtained using the “peak-picking” algorithm following the modal analysis of the physiologically detailed nonlinear model of the four tES perturbation pathways, using 10 different seeds for the white Gaussian noise. The “peak-picking” method is a local single-degree-of-freedom method where the peaks for each mode are considered independently. Here, the natural frequencies across all four tES perturbation pathways were found in the physiological frequency range of 0.01–0.2 Hz. Figure 3 depicts the boxplot of these natural frequencies within 0.01–0.2 Hz across 10 different runs (with different seeds; see Table 1) of the modal analysis, which were found to be comparable for the four tES perturbation pathways—Pathway 1: tES perturbing vessel response through the synaptic potassium pathway; Pathway 2: tES perturbing vessel response through the astrocytic pathway; Pathway 3: tES perturbing vessel response through the perivascular potassium pathway; and Pathway 4: tES perturbing vessel response through the SMC pathway.

**Figure 4:**
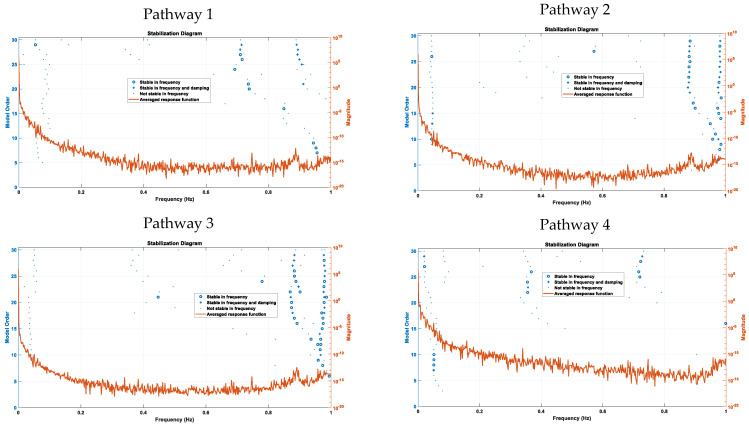
Stabilization diagrams obtained for the four tES perturbation pathways.

We also applied a global multiple-degree-of-freedom method, LSCE, where the parameters for all modes were estimated simultaneously from multiple frequency-response functions. Figure 4 shows the stabilization diagrams and outputs of the natural frequencies of the poles that were stable in frequency, which were found for lower (<0.2 Hz) frequencies mainly for Pathway 4, and for higher (>0.2 Hz) frequencies mainly for Pathways 2 and 3. Here, many stable modes were found near 1 Hz, mainly for tES perturbation Pathways 2 and 3. Then, stable modes in the physiological frequency range of 0.01–0.2 Hz were mainly found for Pathway 4. Since Pathway 4 is the last for the vessel response (see Figure 1b) so this led to comparable natural frequencies from the modal analysis (see Figure 3) for the four nested tES perturbation pathways (see Figure 1b) The poles and the damping parameters associated with the linearized models of the four tES perturbation pathways are listed in Table S2 (Supplementary Materials: Modal analysis of the physiologically detailed neurovascular model).

Our results provide a mechanistic understanding of the four physiologically detailed tES pathways in the NVU in terms of their frequency-response functions [45]. Specifically, stable modes (see Figure 4) were found in the 0–0.05 Hz range in tES Pathway 4, which could be leveraged to develop tES interventions perturbing vessel response via the SMC pathway, including diffusing nitric oxide from postsynaptic signaling. Vascular factors contribute to cerebrovascular disease as well as mild cognitive impairment and dementia [22], which are predicted to affect 152 million people by 2050 (Alzheimer’s Disease International London, UK, 2019). Various physiologically relevant frequency bands have already been identified in the literature: 0.6–2 Hz and 0.145–0.6 Hz are related to cardiac and respiratory function, respectively, 0.052–0.145 Hz is associated with smooth muscle cell activity, and 0.021–0.052 Hz may reflect smooth muscle cell autonomic innervation [12]. Moreover, many stable modes (see Figure 4) were found near 1 Hz for tES Pathways 2 and 3, which could be leveraged to develop tES interventions perturbing vessel response through the astrocytic and perivascular potassium pathways. Here, increases in interstitial potassium concentration can modulate the neurovascular coupling [9], which is likely to change the transfer function model, including its steady-state gain via Kir channels [48]. In a computational modeling study within the frequency range of 0.1–10 Hz, Yashika et al. [45] found that the vessel oscillations were more sensitive to transcranial oscillating-current stimulation than to transcranial alternating-current stimulation, and the entrainment effects were more pronounced for lower frequencies. Here, Kir 2.1 channels on the endothelium and Kir 2.2 channels on the pericytes can modulate [6,47] the neurovascular coupling—as shown in Figure 1a—which may have therapeutic potential in aging and Alzheimer’s disease [49]. Therefore, investigation of the tES modulation of neurovascular coupling and the role of CBF in facilitating neural processing is crucial [50]. Here, prior works [7,8,24] have found that tDCS can change the neurovascular coupling status, which may be mediated by the Kir potassium channels in the mural cells [6], thereby changing the neurovascular system’s sensitivity, leading to aftereffects. Such modulation of the neurovascular system’s sensitivity can change the transfer function (see Figure 1b) from the tES current density (input), leading to vessel response in terms of diameter changes (output), which can be found by tracking the steady-state gain, e.g., using Kalman-filter-based online parameter estimation of an ARX model [8]. Then, short-term (<150 s) tDCS can affect the hemodynamic response [9], including the postulated norepinephrine [32] vasoconstricting perivascular pathway vis-à-vis the “initial dip” [6]. Since we found stable modes in the 0–0.05 Hz range for Pathway 4 (tES perturbing vessel response through the SMC pathway) with the Nyquist frequency at 0.1 Hz, a maximum TR = 10 s is feasible for the functional magnetic resonance imaging (fMRI)–tDCS studies [51].

## 3. Grey-Box Modeling of fNIRS of tDCS’s Effects—A Chronic Stroke Case Series

In this study, we aimed to compare the cerebellar tDCS-evoked fNIRS HbT response with primary motor cortex (M1) tDCS-evoked fNIRS HbT response at the lesional and the non-lesional hemispheres in chronic ischemic stroke. Prior works in healthy subjects have shown that tDCS-induced excitability changes in the left M1 are correlated with the CBF changes in the left sensorimotor region; however, tDCS-induced alterations in CBF could only partially account for cortical excitability changes [36]. Therefore, although CBF changes were likely evoked by the electric field, they could not completely account for the cortical excitability related to neuroplastic changes and their variability [52]. Here, longer plasticity-inducing tDCS is postulated to result in complex bidirectional communication in the neurovascular unit (NVU) [53,54] vis-à-vis cortical excitability changes, which could only be partially accounted for by changes in CBF [52]. Therefore, it is important to investigate the underlying mechanisms of the hemodynamic response to tDCS-induced electric fields in the neurovascular brain tissue—i.e., CVR to tDCS [54]—in health and disease.

In healthy tissue, CVR is a compensatory mechanism where blood vessels respond to the vasoactive stimulus from tES, which can be related to the neurovascular coupling (NVC) [55]. This capacity of the blood vessels to dilate in response to the vasoactive stimulus can be hampered in various cerebrovascular diseases, including chronic stroke [56,57]. In this study, we investigated CVR during tDCS based on grey-box linear systems analysis [9]. Here, completely data-driven black-box systems approaches can provide a correlate of neural and hemodynamic response at an abstract level under the assumption of NVC at the cellular level. However, these black-box system approaches do not aim to explicitly capture the underlying mechanisms of action. Considering the evidence of modulatory consequences of tDCS on blood vessels, which can be via neuronal and non-neuronal cells [58], a deeper understanding of the signaling pathways is important for a mechanistic understanding [6]. Figure 1b shows the four nested pathways that were physiologically modeled in our prior work [9].

Pathway 1: tES-evoked synaptic potassium → vessel circumference

Pathway 2: tES-evoked astrocytic current channel → vessel circumference

Pathway 3: tES-evoked perivascular potassium → vessel circumference

Pathway 4: tES-evoked smooth muscle cell → vessel circumference

Retrospective data [33] from a convenience sample of six male chronic (>6 months’ post-stroke) ischemic stroke subjects who volunteered for the cerebellar tDCS (ctDCS) study are listed in Table 1. T1-weighted MRI was available from All the India Institute of Medical Sciences, New Delhi, India, and we selected chronic stroke survivors with cerebral lesions but with an intact cerebellum. Written informed consent was obtained from each subject, and the multicenter research protocol was approved by the institutional reviews boards of the All India Institute of Medical Sciences, New Delhi, India (IEC-129/07.04.2017) and the Indian Institute of Technology Gandhinagar, India (IEC/2019-20/4/UL/046). Retrospective data [7] from four chronic (>6 months) ischemic stroke survivors (one female) were used for the M1 tDCS analysis, as listed in Table 1.

M1 tDCS was delivered with the anode (SPONSTIM-8, Neuroelectrics, Spain) placed at Cz (international 10–20 system of scalp sites [59]) and the cathode (SPONSTIM-25, Neuroelectrics, Barcelona, Spain) over the left supraorbital notch, and conducted at an anode current density of 0.526 A/m^2^. Cerebellar tDCS (ctDCS) was delivered at PO9h–PO10h (international 10–05 system of scalp sites [59]) with a 3.14 cm^2^ (PISTIM, Neuroelectrics, Barcelona, Spain) circular anode placed at the contralesional side, while the cathode (PISTIM, Neuroelectrics, Barcelona, Spain) was placed at the ipsilesional side at a higher current density of 6.4 A/m^2^. With 0.526 A/m^2^, M1 tDCS was postulated to affect the neurovascular tissue mainly at the surface of the cortex while, with 6.4 A/m^2^, focal ctDCS was postulated to reach the deep dentate nuclei of the cerebellum [60] and affect neurovascular tissue at the M1 region via cerebrocerebellar connections. Therefore, the fNIRS signal was sampled at 10 Hz and analyzed from the M1 region with the source (760/850 nm) placed at Cz and the detectors placed at FC1, FC2, CP1, and CP2 (~2.5 cm inter-optode distance) in the M1 tDCS study. In the ctDCS study, the detector was placed at Cz and the sources (760/850 nm) were placed at FC3, FC4, CP3, and CP4 (~3.5 cm inter-optode distance). Computational head modeling for tES current density distribution was performed using ROAST [61], and the fNIRS sensitivity analysis was performed using AtlasViewer [62]. Motion artifact detection and correction were performed using combined spline interpolation and Savitzky–Golay filtering [63] in HOMER3 (https://github.com/BUNPC/Homer3, accessed on 17 September 2020), which is an open-source software package for MATLAB (MathWorks Inc., Natick, MA, USA). Then, a modified Beer–Lambert law was used to convert the detectors’ raw optical data into optical density, and the conversion of optical density to changes in HbT was performed, followed by downsampling to 1 Hz.

The study used a physiologically constrained linear model [9] to track the HbT changes due to acute (0–60 s: 0–30 s ramp-up and 30–60 s steady-state tDCS) effects of the tDCS current density on the neurovascular tissue. Induced current density in the ohmic head model was assumed to have a vasoactive influence via a linear transfer function mapping to the vasoactive signal. The process of transforming tDCS current density to a vasoactive signal was represented by a first-order linear filter, $v\left(s\right)=\frac{K}{s/\tau +1}J_{tdcs}$, where K is the gain mapping the current density at the electrode–skin interface ($J_{tdcs}$) to that at the neurovascular tissue, and τ is the time constant to the vasoactive (v(s)) signal (in the s-domain) [64]. Physiologically detailed healthy NVC models from published literature were simulated using the ODE23TB solver in Simulink (MathWorks, Inc., Natick, MA, USA), as detailed by Arora et al. [9]. Then, model reduction of the four pathways from physiologically detailed healthy NVC models was performed using the Simulink (MathWorks, Inc., Natick, MA, USA) linear analysis tool. This tool allowed linearization of nonlinear models at the baseline operating point of the physiologically detailed NVC models. Therefore, the linearized grey-box model was constrained by the individual physiology of the four pathways from physiologically detailed healthy NVC models published previously [9]. Then, the values of the free parameters (i.e., poles and zeros) of the grey-box linear model were updated using the “Refine Existing Model” approach in the System Identification toolbox (MathWorks, Inc., Natick, MA, USA) to fit to the experimental pathological fNIRS HbT changes (0–60 s) in response to tDCS in chronic stroke survivors.

After minimizing the mean squared error E(N^2^[n]) of the parametric grey-box linear model to fit to the initial 60 s (30 s ramp-up and 30 s steady-state tDCS) fNIRS HbT changes in response to tDCS, the fNIRS HbT signal without that pathway influence can be written as $Y\left[n\right]=X\left[n\right]-TF\left[n\right]*J_{tdcs}\left[n\right]$, where TF[n] is the grey-box transfer function, X[n] is the original fNIRS time series, and Y[n] is the fNIRS time series without the corresponding pathway influence [65]. Here, the initial 60 s of fNIRS HbT response was assumed to be unaffected by the cortical excitability changes, since prior works showed that the aftereffects of neuroplastic excitability (mainly calcium-dependent) start after 3 min following the onset of tDCS [66]. We also computed the M1 tDCS and ctDCS effect size on HbT before and after removing the smooth muscle cells’ effects (norepinephrine [13] receptors are present on the pial arterial smooth muscle cells [14,15]). The CVR to tDCS at the ipsilesional and contralesional hemispheres was compared based on Cohen’s $d=\frac{\bar{contralesional}-\bar{ipsilesional}}{\sqrt{\frac{s_{contralesional}^{3}-s_{ipsilesional}^{3}}{2}}}$, where $\bar{contralesional}$ *and* $\bar{ipsilesional}$ are the means of the two hemisphere HbT responses, and $s_{contralesional\;}^{}and\;s_{ipsilesional}^{}$ are their standard deviations, respectively. Paired-samples *t*-tests were used to measure significant differences in the HbT response between the hemispheres.

Grey-box model transfer functions from physiologically detailed healthy model: The linearized grey-box model transfer functions obtained from the physiologically detailed model [9] are given in the Supplementary Materials (Grey-box modeling of fNIRS of tDCS effects—a chronic stroke case series), as is the model response to the tDCS pulse. The response function of Pathway 1 to the known synaptic potassium → vessel circumference hemodynamic responses peaked ~5 s [67]. Here, nitric oxide as a postsynaptic signaling molecule is postulated to be involved later, likely following facilitation of glutamate transmission by longer-duration tDCS. Pathway 4 peaked ~2 s, which may be related to the cerebral autoregulation time constant [68], and can raise safety concerns vis-à-vis the effects of tDCS [69]. Here, the dynamic system model is postulated to capture the autonomic ability of cerebral arterioles to change blood volume (HbT changes) following a vasoactive tDCS stimulus [69].

**Figure 5:**
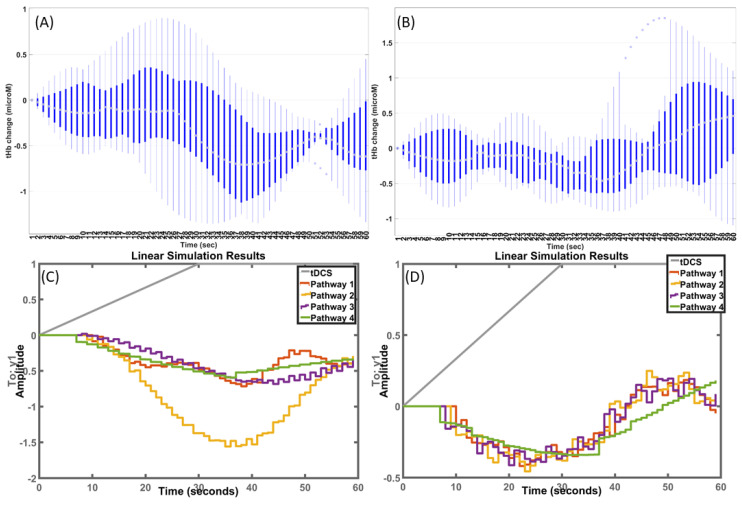
Box-plots of HbT (μM) time series for 0–60 s of M1 tDCS (0–30 s ramp-up and 30–60 s steady-state) are shown at the (**A**) ipsilesional and (**B**) contralesional hemispheres. Four pathways fitted to fNIRS HbT time-series data at the (**C**) ipsilesional and (**D**) contralesional hemispheres are also shown.

Grey-box model transfer functions fitted to the post-stroke HbT—M1 tDCS data: A Levenberg–Marquardt least-squares search was used for iterative parameter estimation in MATLAB(“tfest”) to find a stable model where the regularization pulled the parameters towards the parameter values of the initial grey-box model (from the physiologically detailed healthy model [9]). Boxplots of fNIRS HbT time series from the ipsilesional and contralesional hemispheres are shown in Figure 5A,B, respectively, with simulated results from fitted models of the four pathways shown in Figure 5C,D, respectively.

**Figure 6:**
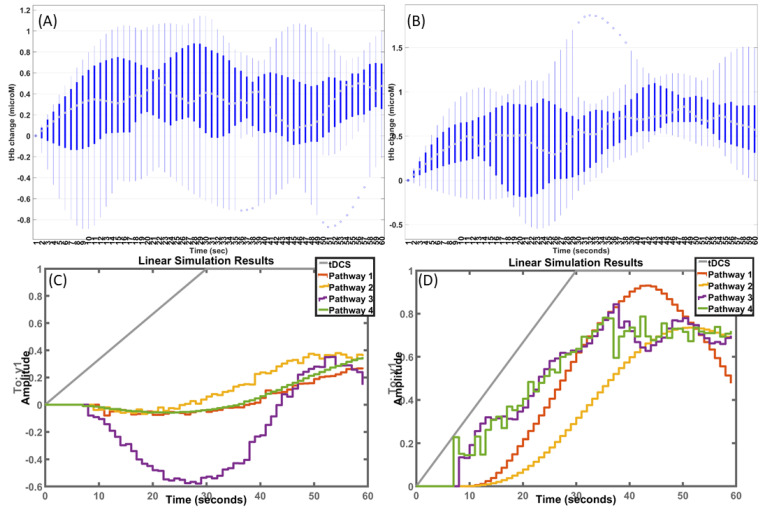
Boxplots of HbT (µM) time series for 0–60 s of ctDCS (0–30 s ramp-up and 30–60 s steady-state) are shown at the (**A**) ipsilesional and (**B**) contralesional hemispheres. Four pathways fitted to fNIRS HbT time-series data at the (**C**) ipsilesional and (**D**) contralesional hemispheres are also shown.

Grey-box model transfer functions fitted to the post-stroke HbT—ctDCS data: Boxplots of fNIRS HbT time series from the ipsilesional and contralesional hemispheres are shown in Figure 6A,B, respectively, with simulated results from fitted models of the four pathways shown in Figure 6C,D, respectively. Iterative parameter estimation was performed for the fNIRS HbT time series for 0–60 s of ctDCS (0–30 s ramp-up and 30–60 s steady-state) at the ipsilesional and contralesional hemispheres. 

**Figure 7:**
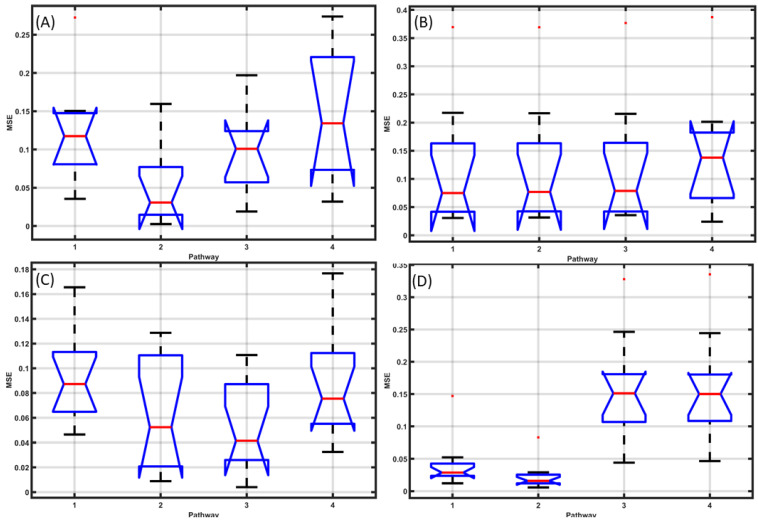
Mean squared error (MSE) with M1 tDCS for HbT at the (**A**) ipsilesional and (**B**) contralesional hemispheres. MSE with ctDCS for HbT at the (**C**) ipsilesional and (**D**) contralesional hemispheres.

**Figure 8:**
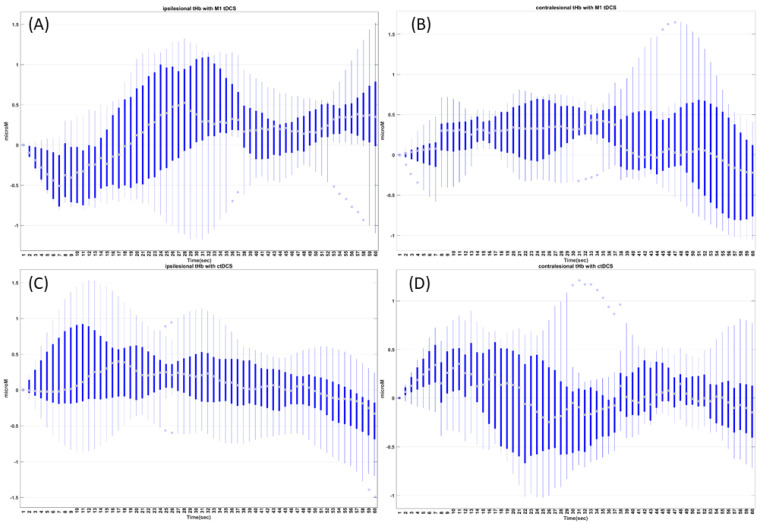
Boxplots of filtered HbT (µM) time series for 0–60 s of M1 tDCS at the (**A**) ipsilesional and (**B**) contralesional hemispheres, and for ctDCS at the (**C**) ipsilesional and (**D**) contralesional hemispheres.

In case of the grey-box model transfer functions fitted to the post-stroke HbT—M1 tDCS data, the mean squared error (MSE) for different pathways are shown in Figure 7A,B, where Pathway 2 performed the best (lowest MSE) for ipsilesional HbT (Figure 7A), while Pathways 1–3 performed well for contralesional HbT (Figure 7B). Then, in case of grey-box model transfer functions fitted to the post-stroke HbT—ctDCS data, the MSE are shown in Figure 7C,D, where Pathways 2–3 performed well for ipsilesional HbT (Figure 7C), while Pathways 1–2 performed well for contralesional HbT (Figure 7D). The HbT response was significantly different (paired-samples *t*-test, *p* < 0.05) at the ipsilesional hemisphere compared to the contralesional hemisphere in the case of M1 tDCS (as shown in Figure 5A and Figure 7B), and the effect size based on Cohen’s d was found to be very large (=2.49). Moreover, the HbT response was significantly different (paired-samples *t*-test, *p* < 0.01) at the ipsilesional hemisphere compared to the contralesional hemisphere in the case of ctDCS with at a very large Cohen’s d (=2.33). Then, the Pathway 4 transfer function fitted to the post-stroke HbT data (shown in Figure 5C,D) was used for removing the Pathway 4 effects, i.e., the tDCS effects from smooth muscle cells → vessel circumference. The fNIRS HbT responses without the Pathway 4 effects are shown as boxplots in Figure 8 across all conditions: M1 tDCS, ctDCS, ipsilesional hemisphere, and contralesional hemisphere. Here, the removal of the Pathway 4 effects reduced Cohen’s d (=0.36) and the interhemispheric difference (*p* = 0.71) in the case of M1 tDCS. Furthermore, the removal of the Pathway 4 effects reduced Cohen’s d (=−0.266) and the interhemispheric difference (*p* = 0.72) in the case of ctDCS.

**Figure 9:**
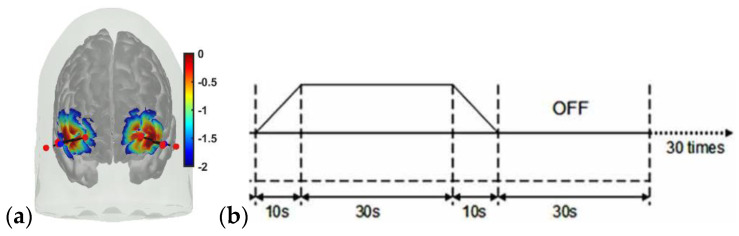
(**a**) Sensitivity profile of the optode montage (red dots are sources at long separation and short separation from detectors; blue dots are detectors). The sensitivity values are displayed logarithmically, with a default range of 0.01 to 1, or −2 to 0 in log10 units; (**b**) 30 s ON–30 s OFF tDCS paradigm with 10 s ramp-up/10 s ramp-down—repeated 30 times in a block design.

Pathway 4 effects can postulated via norepinephrine [13] receptors on the pial arterial smooth muscle cells [14,15], which may be relevant at the onset (<150 s after tDCS) of tDCS [9]. Moreover, tES-evoked LC-NE activity via projections innervating the cerebral vasculature can have therapeutic effects, e.g., cerebellar tDCS (ctDCS) electrodes [33] may stimulate the ascending fibers of the occipital nerve [70]. Here, tES can be optimized to stimulate the peripheral nerves [32] and the LC-NE system. Activation of the LC-NE system lead to a psychosensory pupil response in the dilation of the pupil. Significantly, short-duration (<3 min) tDCS can have physiological effects, where the biological effects can extend beyond the intended transient sensations [71]. In fact, the onset response in the case of short-duration (considered sham) tDCS may explain the hidden source of variability in its effects [71]. In principal accordance, pupil dilation was investigated along with prefrontal fNIRS (see Figure 9a) under 2 mA tDCS, with the anode at FC5 and the cathode at FP2 (electrode montage from [72])—as presented below.

## 4. Black-Box Modeling of Prefrontal fNIRS–Pupillometry of the Effects of Short-Duration Frontal tDCS—A Healthy Case Series

Our prior works [6] have presented grey-box modeling results for tDCS-evoked hemodynamic response; however, tDCS-evoked arousal effects were not explicitly modeled physiologically [9]. In the absence of a grey-box model of tDCS-evoked arousal effects, we tested the feasibility of a black-box modeling approach in this study. After providing written informed consent, five young (aged between 20 and 30 years) and healthy male subjects were recruited. Case study procedures were performed according to the local regulations for research on human subjects at the University at Buffalo.

Portable brain imaging was performed using four sources and two detectors of a portable fNIRS (Octamon+, Artinis Medical Systems, Gelderland, The Netherlands) placed at the forehead (see the optode montage from [20]). Figure 9a shows the sensitivity profile of the optode montage that covered the middle frontal gyrus (orbital part) and superior frontal gyrus (dorsolateral part) bilaterally [62]. The subjects were seated comfortably indoors in a windowless room while they looked at an evenly painted non-glossy white wall with fluorescent lighting (intensity ~4000 lux). The eyes were monitored using the open-source platform Pupil Core (Pupil Labs, Berlin, Germany). Then, 2 mA tDCS was applied in a 30 s ON–30 s OFF temporal sequence with 10 s ramp-up/10 s ramp-down, which was repeated 30 times in a block design [54] (see Figure 9b). Stimulation and multimodal data acquisition were synchronized using Lab Streaming Layer (https://labstreaminglayer.readthedocs.io/info/supported_devices.html; accessed on 4 August 2022).

Preprocessing of the fNIRS data was performed using the standard open-source package HOMER3 (https://github.com/BUNPC/Homer3; accessed on 4 August 2022). The fNIRS preprocessing pipeline was as follows: First, intensity was converted to optical density, and then motion artifacts were detected and filtered with the help of the Savitzky–Golay filtering method with default parameters. Then, the optical density was bandpass-filtered in the neurovascular coupling band at 0.01–0.1 Hz, and then converted to chromophore concentration (oxyhemoglobin: HbO, deoxyhemoglobin: HHb) changes with the unit partial pathlength factor. Here, vasoconstriction effects (e.g., due to tDCS-evoked norepinephrine) on the pial arteries can lead to an initial dip in the blood volume (and total hemoglobin: HbT) [9] in the long-separation (LS) fNIRS channels, since pial arteries start the pressure-driven blood pathway to the cortex (reviewed in Schmid et al. [44]). Moreover, hemodynamics in the short-separation (SS) fNIRS channels can be affected by tDCS, and skin blood flow artefacts need to be removed from the LS HbT data for NVU modeling. Pupil Capture (Pupil Labs, Berlin, Germany) recorded videos of the left and the right eyes at 120 fps in 400 × 400 pixel resolution. Pupil Core reports pupil diameter in mm, provided by the pye3d model [73]. The time series from the left and right eyes were averaged in the young and healthy subjects.

**Figure 10:**
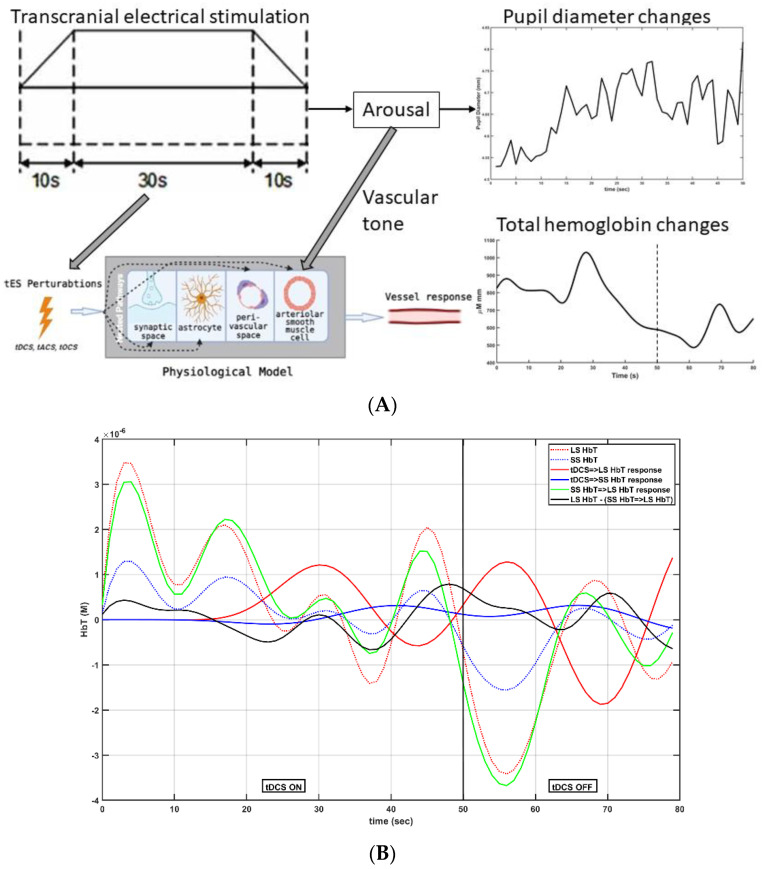
(**A**) Transcranial electrical stimulation (tES)--evoked arousal leads to changes in the pupil diameter as well as the vascular tone, affecting the evoked hemodynamic response. (**B**) An illustrative example of HbT responses in long-separation (LS) and short-separation (SS) fNIRS channels. LS HbT: long-separation total hemoglobin changes, SS HbT: short-separation total hemoglobin changes, tDCS => LS HbT response: transfer function response with tDCS waveform input and LS HbT output, tDCS => SS HbT response: transfer function response with tDCS waveform input and SS HbT output, SS HbT => LS HbT response: transfer function response with SS HbT input and LS HbT output, LS HbT--(SS HbT => LS HbT): SS HbT => LS HbT response subtracted from LS HbT.

Figure 10A shows the postulated relationship between the pupil diameter changes and the total hemoglobin concentration changes (HbT = HbO + HHb) due to tDCS-evoked arousal. A state-space model for arousal via reduction of a regularized ARX model (”ssregest” in MATLAB) was found between the tDCS trapezoidal waveform input and the pupil diameter changes output [74], which could account for the carryover effect. Moreover, transfer functions were estimated (“tfest” with the EnforceStability estimation option as true in MATLAB) for LS and SS HbT responses, and the estimated model responses (“lsim” in MATLAB) for the tDCS ON–OFF period are shown in Figure 10B. Figure 10B shows an illustrative example of averaged LS and SS HbT signals across the tDCS ON–OFF period (total 80 s). The model from tDCS waveform input to LS HbT output (tDCS => LS in Figure 10B) was initialized with a reduced-dimensions model (eight poles, two zeros [9]) for Pathway 3: perivascular potassium → vessel circumference. The model from tDCS waveform input to SS HbT output (tDCS => SS in Figure 10B) was initialized with a reduced-dimensions model (six poles, one zero [9]) for Pathway 4: smooth muscle cell → vessel circumference. The model from SS HbT input to LS HbT output (SS => LS in Figure 10B) was a static gain model. All of the transfer functions are presented in the Supplementary Materials (Black-box modeling of prefrontal fNIRS–pupillometry of the effects of short-duration frontal tDCS—a healthy case series).

The transfer function from SS HbT input to LS HbT output provided the estimation of the skin blood flow artefact that was removed from the LS HbT signal. Then, the transfer function between the tDCS trapezoidal waveform input and the skin-artefact-free LS fNIRS HbT output was computed. The state-space model via reduction of a regularized ARX model between the pupil diameter changes input and the skin-artefact-free LS fNIRS HbT output provided some insights. For example, an anti-correlation relationship between tDCS-evoked pupil diameter changes (pupil dilation) and the blood volume changes (HbT) was found. The impulse response (“impulseplot” in MATLAB) and power spectrum (“spectrum” in MATLAB) of all of the state-space models of each subject are presented in the Supplementary Materials (Black-box modeling of prefrontal fNIRS–pupillometry of the effects of short-duration frontal tDCS—a healthy case series). Then, the step-response characteristics (“stepinfo” in MATLAB) for the dynamic system model between the tDCS trapezoidal waveform input and the prefrontal average HbT changes (across all fNIRS channels) output is shown in Table 2a, between the tDCS trapezoidal waveform input and the pupil diameter changes output are shown in Table 2b, while those between the pupil diameter changes input and the average HbT changes (across all fNIRS channels) output are shown in Table 2c. Here, the rise time—i.e., the time it takes for the response to go from 10% to 90%—was fastest (2.54 ± 0.44 s) for the model between the tDCS trapezoidal waveform input and the prefrontal average HbT changes output (Table 2a), followed by the dynamic system model between the tDCS trapezoidal waveform input and the pupil diameter changes output (7.16 ± 5.01 s) and the dynamic system model between the pupil diameter changes input and the average HbT changes output (10.02 ± 3.01 s). Therefore, it can be postulated that tDCS-evoked hemodynamic response has an immediate onset that is faster than the effect on the pupil diameter. The immediate onset has the fastest rise time (2.54 ± 0.44 s)—i.e., ~2 s, comparable to the cerebral autoregulation time constant [68]—for the dynamic system model between the tDCS trapezoidal waveform input and the prefrontal average HbT changes output.

Granger causality and block exogeneity tests for vector autoregression (VAR) models were conducted using MATLAB (MathWorks, Inc., Natick, MA, USA) to check whether the tDCS waveform was block exogenous, i.e., the tDCS waveform does not Granger-cause changes in pupil diameter (pupil dilation) and blood volume (HbT) in the multivariate system. First, the time-series analysis of the tDCS-evoked changes in pupil diameter (pupil dilation) and blood volume (HbT) showed that the autocorrelation function decreased slowly, while the partial autocorrelation function converged to zero after about eight lags. An augmented Dickey–Fuller test (“adftest” in MATLAB) confirmed that all signals were stationary (*p* < 0.025). The innovation errors were investigated for normality (*p* < 0.025), and the remaining autocorrelation structure of the innovation error was found to be negligible. Then, a VAR model for the tDCS waveform, HbT time series, and pupil dilation time series was fitted, and the block exogeneity test rejected the claim that the tDCS waveform is not a one-step Granger-cause of the HbT time series and pupil dilation time series (*p* < 0.05). The results of the VAR model analysis are presented for each subject in the Supplementary Materials (Black-box modeling of prefrontal fNIRS–pupillometry of the effects of short-duration frontal tDCS—a healthy case series).

It is postulated that VAR models may be leveraged for immediate control of hemodynamics using model predictive control (MPC). MPC uses an internal model for making predictions of the system behavior, considering the dynamics over a predefined prediction horizon, for optimizing the control actions. For online operations, MPC operates in a receding-horizon fashion, i.e., new system measurements and new predictions into the future are made at each time step. Here, MPC can be based on a minimal realization of transfer functions for the four nested pathways for NVU [9], where tES current density (input) can perturb a state variable at each of the four NVU compartments to perturb the vessel volume response (output).

## 5. Human-in-the-Loop Optimization for Model Predictive Control of tES-Evoked HbT

**Figure 11:**
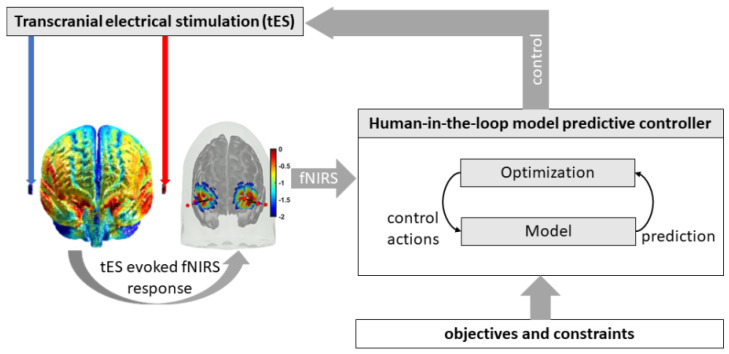
An illustrative model predictive control scheme.

Yashika et al. [45] found that the vessel oscillations were more sensitive to transcranial oscillating-current stimulation (tOCS) than to transcranial alternating-current stimulation (tACS), and the effects were more pronounced for lower frequencies within the frequency range of 0.1–10 Hz. Here, increases in interstitial potassium concentration can modulate the neurovascular coupling [9], i.e., Pathway 3: tES perturbing vessel response through the perivascular potassium pathway. Then, a change in the transfer function model from tDCS waveform (input) to the HbT response (output) can change the steady-state gain of the system that is postulated via the Kir channels [48]. Therefore, in a healthy case study, we aimed to test the feasibility of human-in-the-loop optimization of tOCS and tACS with the electrodes placed at the “FT7” and “FT8” EEG locations (10-10 system) to maximize the steady-state gain (i.e., moles of HbT change per mA of tES current intensity) of Pathway 3 (reduced-dimensions model [9]), which was fitted (“tfest” with the EnforceStability estimation option as true in MATLAB) to the tES-evoked skin-artefact-free LS HbT data from frontal fNIRS optodes (sensitivity profile shown in Figure 9a). The electric fields computed with the ROAST package [61] are presented in the Supplementary Materials (Human-in-the-loop optimization for model predictive control of tES-evoked HbT). The model predictive control scheme is shown in Figure 11, where the skin-artefact-free LS HbT response was used to optimize tOCS control actions, i.e., the tES waveform was parametrized by a direct-current (DC) intensity, an alternating-current (AC) amplitude, and an AC frequency. The amplitude of the AC waveform had a fixed ramp-up time (=10 s), ramp-down time (=10 s), and duration (=10 s). Repeated measures of the 30 s epochs of tOCS-evoked skin-artefact-free LS HbT response were used by the CMA-ES algorithm [46] to optimize the DC intensity, AC amplitude, and AC frequency for the subsequent iteration of the 30 s tOCS ON period to maximize the steady-state gain of the system (i.e., the Pathway 3 model fitted to tOCS ON HbT data). Here, the mean of the set of current intensity perturbations at each iteration—which were evaluated independently—is the optimal current intensity at that stage of the optimization.

**Figure 12:**
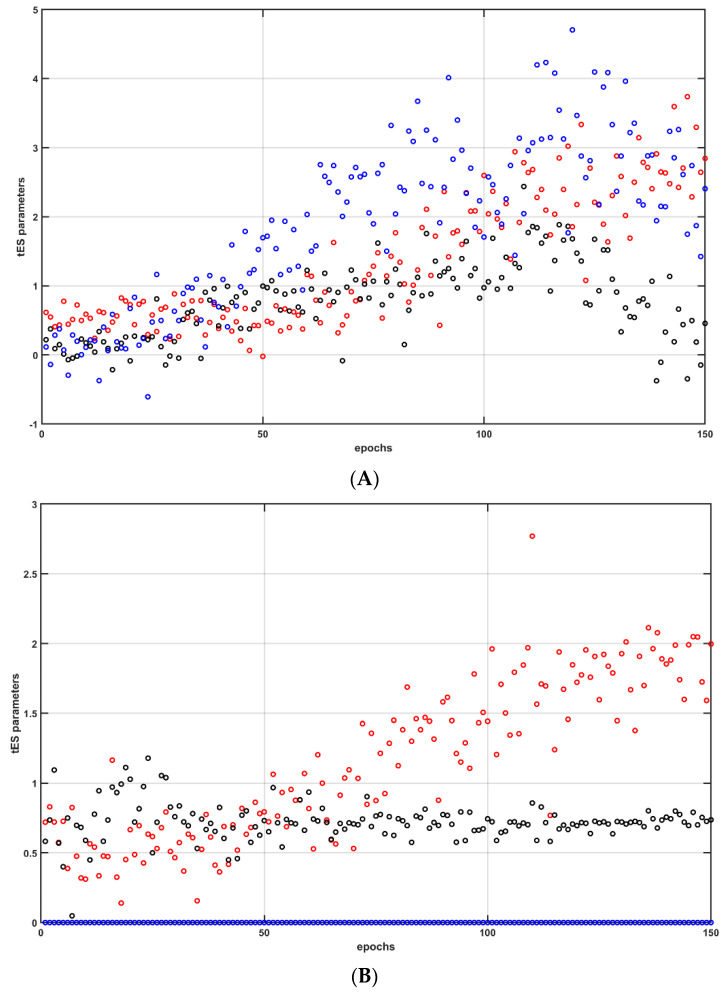

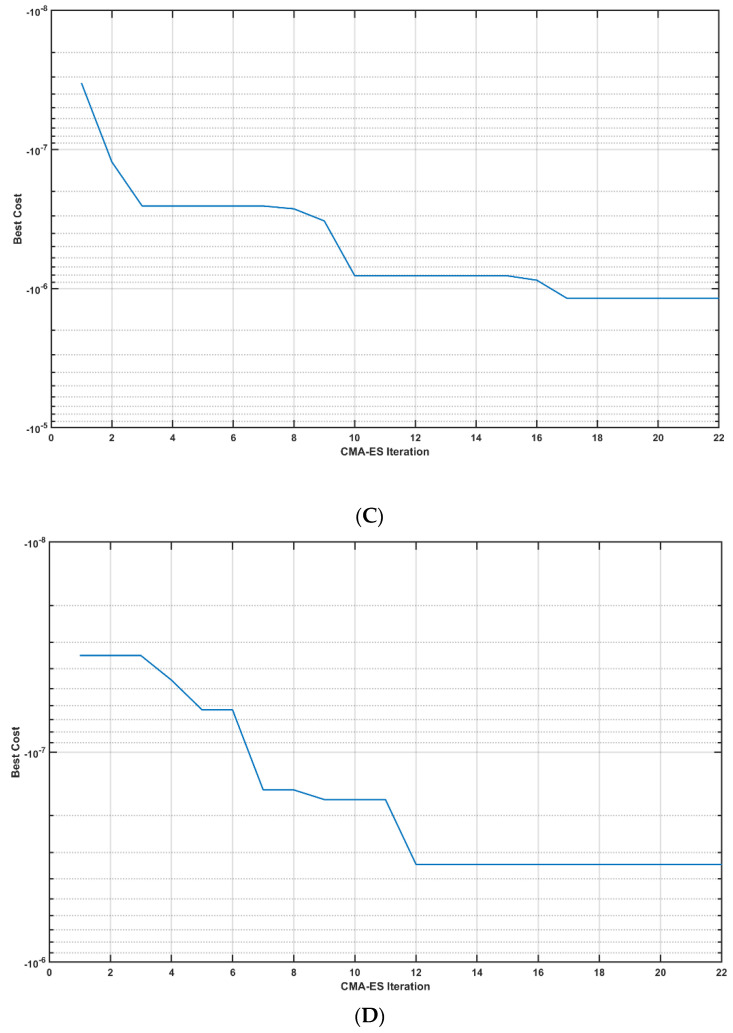
Human-in-the-loop optimization using a covariance matrix adaptation evolution strategy (CMA--ES): (**A**) tOCS parameters: DC intensity in mA (blue), AC amplitude in mA (red), and AC frequency in Hz (black). (**B**) tACS parameters: DC intensity = 0 mA (blue), AC amplitude in mA (red), and AC frequency in Hz (black). (**C**) Best cost (i.e., negative steady-state gain of HbT in M) for tOCS over 22 iterations of CMA--ES. (**D**) Best cost (HbT in M) for tACS over 22 iterations of CMA--ES.

A case study (male, 44 years old) was performed after the provision of written informed consent according to the local regulations for research on human subjects. Case report procedures were performed according to the local regulations for research on human subjects at the University at Buffalo. Portable brain imaging was performed using four sources and two detectors of a portable fNIRS (Octamon+, Artinis Medical Systems, Gelderland, the Netherlands) placed at the forehead (see the optode montage from [20]). Figure 9a shows the sensitivity profile of the optode montage that covered the middle frontal gyrus (orbital part) and superior frontal gyrus (dorsolateral part) bilaterally [62]. Stimulation and multimodal data acquisition were synchronized using Lab Streaming Layer (https://labstreaminglayer.readthedocs.io/info/supported_devices.html; accessed on 4 August 2022). Online preprocessing of the 30 s fNIRS data epoch after the end of the tOCS ON period was performed in MATLAB using functions from the open-source HOMER3 package (https://github.com/BUNPC/Homer3; accessed on 4 August 2022). The fNIRS preprocessing pipeline was as follows: First, intensity was converted to optical density, and then motion artifacts were detected and filtered with the help of the Savitzky–Golay filtering method with default parameters. Then, the optical density was bandpass-filtered in the neurovascular coupling band at 0.01–0.1 Hz, and then converted to chromophore concentration (oxyhemoglobin: HbO, deoxyhemoglobin: HHb) changes with the unit partial pathlength factor. We averaged all of the long-separation (LS) HbT channels and short-separation (SS) HbT channels for online model estimation following the 30 s tDCS ON perturbation, using the MATLAB toolkit MatNIC2 (Neuroelectrics, Barcelona, Spain). First, the static gain transfer function from SS HbT input to LS HbT output provided the estimation of the skin blood flow artefact during the tOCS ON epoch, which was removed from the LS HbT signal. Then, the steady-state gain (“dcgain” in MATLAB) of Pathway 3 fitted (“tfest” with the EnforceStability estimation option as true in MATLAB) to the 30 s epoch of the tOCS-evoked skin-artefact-free LS HbT data was estimated. Then, the CMA-ES algorithm [46] computed the DC intensity, AC amplitude, and AC frequency for the next perturbation, and the iterations continued for 150 epochs, as shown in Figure 12A. Figure 12B shows the case where the DC intensity was set to zero and only the AC amplitude and AC frequency were optimized (i.e., tACS condition). CMA-ES took seven epochs of tES perturbation to deliver one iteration of optimization. The change in the best cost (i.e., the negative of steady-state gain, HbT in µM) from seven epochs for each iteration of the CMA-ES optimization is shown in Figure 12C,D, for tOCS and tACS, respectively. Here, tOCS reached a higher steady-state gain than tACS for Pathway 3 over 22 CMA-ES iterations (took ~75 min), which was consistent with our computational modeling results [45]; however, optimal tOCS parameters likely require more epochs of human-in-the-loop optimization when compared to the lesser parameters for tACS. Notably, optimal frequency settled around >0.5 Hz for tACS, which aligned well with the stable modes (see Figure 4) near 1 Hz for tES Pathway 3. Future works should test the feasibility of human-in-the-loop optimization for model predictive control of other pathways, including tES-evoked oxidative metabolic substrates that can provide therapeutic intervention in mild cognitive impairment and T2DM. However, such human-in-the-loop optimization of metabolic states would require the development of computational models to estimate the metabolic state of the tissue.

## 6. Discussion

Our computational perspective paper focused on systems analysis using our published physiologically detailed grey-box [9] and black-box models [8] that provided insights into tDCS-evoked responses in fNIRS data and pupillometry data. We also showed the single-subject feasibility of human-in-the-loop optimization for model predictive control of tES-evoked HbT with a case study. Notably, the AC frequency was found to settle around 1 Hz (~0.8 Hz for tACS) after 22 CMA-ES iterations, while the AC amplitude reached around 2 mA in the tACS condition (see Figure 12B). This “optimal” AC frequency value from CMA-ES was consistent with the results from modal analysis of the tES pathways (see Figure 4), where stable modes were found near 1 Hz, especially for Pathways 2 and 3, meriting mechanistic (vis-à-vis astrocytes, interstitial potassium, etc.) investigation using animal models [75,76]. Moreover, the relationship between fNIRS and pupillometry data merits further investigation vis-à-vis arousal mechanisms. Arousal mechanisms have broad implications for the bedside neuromonitoring of disorders of consciousness [77], including Alzheimer’s disease [78], where monitoring of the neurovascular coupling [3] and pupil dilation [1,2] may be feasible. For bedside neuromonitoring, an important aspect is the neurometabolic state of the brain, which is partially regulated by tonic and phasic norepinephrine activity [79]. In this computational review paper, we showed that tDCS-evoked hemodynamic response and pupil dilation can be related in healthy individuals, postulated to be subserved by arousal mechanisms, and future studies should investigate this relationship in disorders of consciousness, including Alzheimer’s disease. Moreover, subject-specific interactions [80] between the tDCS-evoked LC-NE activity and interstitial potassium concentration, which can modulate neurovascular coupling [9], could provide insights into the interindividual variability in the effects of tES [6]. Furthermore, tES-evoked increase in energy demand can uncover abnormalities [7] of cerebral metabolism that can be elucidated through system analysis of the neuroimaging data. For example, a decreased availability of oxidative metabolic substrates in the NE-depleted cortex can be partially responsible for mild cognitive impairment and “brain fog” [81,82]. Thus, human-in-the-loop optimization for model predictive control of tES-evoked LC-NE activity (measured by pupil dilation) and metabolic substrates (measured by fNIRS) can provide therapeutic intervention in mild cognitive impairment and T2DM.

Increases in CBF and oxyhemoglobin concentration during tES need careful investigation to delineate the two main neurovascular coupling hypotheses—metabolic and neurogenic. The indirect “metabolic hypothesis” states that an increase in neuronal synaptic activity causes additional energy and oxygen demand, causing various vasodilation agents to send signals to the cerebral vasculature for vasodilation, resulting in an increase in CBF and oxyhemoglobin. The increase in oxyhemoglobin can also be explained through the direct “neurogenic hypothesis”, whereby the direct electric field modulation of neuropeptides and neurotransmitters causes a discharge of various vasoactive agents for vasodilation. Hence, the “neurogenic hypothesis” can also be applied to the effects of tDCS on the perivascular nerves, e.g., in the pial vasculature, which will have a compounding effect in changing oxyhemoglobin levels. Therefore, early and late hemodynamic responses to plasticity-inducing tDCS need to be carefully investigated in future works, where the “metabolic hypothesis” vis-à-vis cortical excitability alterations (i.e., polarity-dependent effects of M1 tDCS [66]) should be delineated from the “neurogenic hypothesis” vis-à-vis the effects of tDCS on the perivascular nerves. Here, complex bidirectional interactions can lead to nonlinear dose effects [83], where lower current intensity at the scalp will primarily affect the perivascular nerves in the pial vasculature, while higher current intensity at the scalp may reach deeper in the cortex—a dose–response effect.

We conclude this perspective article with a vision for the future works that need to investigate human-in-the-loop optimization for model predictive control of non-invasive brain stimulation that can be based on the hemo-neural hypothesis [50]. Animal studies have provided some insights into the application of tES, e.g., a study by Han et al. [84] found that the concentration of oxyhemoglobin increased almost linearly during tDCS and then decreased linearly immediately after the termination of tDCS, with a similar rate of change that differed from rat to rat. Han et al. [84] suggested that individual differences in the neuronal aftereffects of anodal tDCS may be related to individual variability in the rate of change of hemodynamic response to tDCS. In the study of Han et al. [84], the concentration of deoxygenated hemoglobin did not show a significant difference during and after tDCS [84]. Here, direct effects of tDCS on cortical astrocytes with astrocytic regulation of blood flow [85] can be possible without changes in the local field potential [86], which can also lead to dilation or constriction of the arterioles [87]. Wachter et al. [88] showed sustained polarity-specific changes in CBF using laser Doppler blood perfusion imaging (LDI), where the duration and the degree of CBF changes depended on the intensity of the current applied. Moreover, Mielke et al. [89], using LDI, showed a regionally limited, long-lasting, and reversible decrease in hemispheric CBF due to cathodal tDCS, which depended on the current intensity as well as the size of the stimulation electrode. Our human-in-the-loop optimization for model predictive control of non-invasive brain stimulation can also be applied to other modalities, e.g., photobiomodulation [90]. Moreover, patient-derived cerebral organoids can facilitate the individualization of non-invasive brain stimulation applications by identifying state–trait differences [91]. Here, patient-derived cerebral organoids can reveal trait differences and gene expression patterns subserving dysregulation of mitochondrial function [92] and metabolic state that may subserve neurometabolic reactivity to non-invasive brain stimulation. For example, a “phase zero” cerebral organoid platform [90] can use dual-polymer sensors in the Matrigel matrix to provide real-time monitoring of glucose and oxygen [93] during stimulation to capture dose–response relationship based on systems analysis. Then, organoid-in-the-loop optimization of certain non-invasive brain stimulation modalities, e.g., photobiomodulation, may be feasible for subsequent model predictive control of non-invasive stimulation in resource-intensive human clinical studies. Future invasive animal studies should investigate the plasticity of the modulation of the mural cells [94] by long-term tES for the mechanistic understanding of the effects of tES on neurovascular and neurometabolic coupling.

Limitations of the current work include the unavailability of fMRI–EEG data [51] to demonstrate the long-term effects of tES on the neurovascular coupling in the whole brain. Here, it is postulated that tES-evoked arousal effects should be more widespread when compared to the direct electric field related effects on the vasculature (and perivascular space) that could not be investigated and delineated with the limited sensitivity of fNIRS. Also, prior works on fNIRS–EEG have shown the feasibility of computational modeling of the effects of tES via neurovascular coupling [8,9]; however, fNIRS technology cannot image subcortical areas. Furthermore, the trade-off between bias (in canonical HRF) and variance (in FIR HRF) that can be achieved by applying mechanistic grey-box modeling of the NVU pathways [51] was not demonstrated.

## Figures and Tables

**Table 1:** Post-stroke subject characteristics and the tDCS target.

Name	Age (years)	Gender	Post-Stroke Period (years)	Affected Hemisphere (Middle Cerebral Artery Stroke)	tDCS Target
P1	44	Male	2	Left	Cerebellar
P2	53	Male	3	Left	Cerebellar
P3	40	Male	1	Right	Cerebellar
P4	38	Male	1	Left	Cerebellar
P5	32	Male	1	Left	Cerebellar
P6	50	Male	2	Right	Cerebellar
P7	31	Male	6	Right	M1
P8	63	Male	5	Left	M1
P9	73	Male	4	Left	M1
P10	76	Female	5	Right	M1

**Table 2:** (**a**) Step-response characteristics for the dynamic system model between the tDCS trapezoidal waveform input and the prefrontal average HbT changes (across all fNIRS channels) output—tDCS2HbT. (**b**) Step-response characteristics for the dynamic system model between the tDCS trapezoidal waveform input and the pupil diameter changes output—tDCS2PD. (**c**) Step-response characteristics for the dynamic system model between the pupil diameter changes input and the average HbT changes (across all fNIRS channels) output—PD2HbT.

(a) tDCS2HbT	Subject 1	Subject 2	Subject 3	Subject 4	Subject 5	Mean	stdev
**RiseTime**	2.4	2.3	2.2	2.5	3.3	2.54	0.439318
**SettlingTime**	9.6	8.7	9.9	10.4	9.4	9.6	0.62849
**SettlingMin**	0.2187	0.2544	0.2882	0.1615	0.1351	0.21158	0.063467
**SettlingMax**	0.5878	0.6019	0.5944	0.587	0.5628	0.58678	0.014687
**Overshoot**	0.7288	12.9567	7.6098	0.118	0.2432	4.3313	5.757355
**Undershoot**	0	0	0	0	0	0	0
**Peak**	0.5878	0.6019	0.5944	0.587	0.5628	0.58678	0.014687
**PeakTime**	4	4.6	4.1	14.6	14.2	8.3	5.574944
**(b) tDCS2PD**	**Subject 1**	**Subject 2**	**Subject 3**	**Subject 4**	**Subject 5**	**Mean**	**stdev**
**RiseTime**	14.2	3.5	8.3	8.5	1.3	7.16	5.00979
**SettlingTime**	16.1	11.3	19.2	12	13.9	14.5	3.221025
**SettlingMin**	0.6813	0.5992	0.7907	0.6583	0.2203	0.58996	0.21797
**SettlingMax**	0.7559	0.665	0.8782	0.7285	0.7287	0.75126	0.078427
**Overshoot**	0	0.665	0	0	0.1329	0.15958	0.288339
**Undershoot**	225.1942	127.3642	14.2983	0	0	73.37134	100.2768
**Peak**	1.7037	0.8472	0.8782	0.7285	0.7287	0.97726	0.411736
**PeakTime**	4.5	4.2	53.9	18.6	20.7	20.38	20.25357
**(c) PD2HbT**	**Subject 1**	**Subject 2**	**Subject 3**	**Subject 4**	**Subject 5**	**Mean**	**stdev**
**RiseTime**	11.7	7.5	6.5	10.6	13.8	10.02	3.007823
**SettlingTime**	20.6	14.1	11	19.3	25.8	18.16	5.77434
**SettlingMin**	0.6935	0.738	0.568	0.7148	0.6868	0.68022	0.065855
**SettlingMax**	0.769	0.819	0.629	0.7922	0.762	0.75424	0.073481
**Overshoot**	0	0	0	0	0	0	0
**Undershoot**	0.5904	0.4861	0	4.4144	0	1.09818	1.873619
**Peak**	0.769	0.819	0.629	0.7922	0.762	0.75424	0.073481
**PeakTime**	33.9	25.6	17.9	50.7	47.9	35.2	14.09503

## Data Availability

Raw data is available on request from Anirban Dutta (adutta@case.edu). Processed data of all subjects are included in the Supplementary materials (Supplementary Materials: Black box modeling—processed subject data).

## Supplementary Materials

The following supporting information can be downloaded at: https://www.mdpi.com/article/10.3390/brainsci12101294/s1.
