# Supplementary material for: Human-in-the-Loop Optimization of Transcranial Electrical Stimulation at the Point of Care: A Computational Perspective
Source: Brain Sci. 2022 Sep 26;12(10):1294. doi: 10.3390/brainsci12101294 (PMC9599464; doi:10.3390/brainsci12101294)
Supplement: Supplementary file 1 [file brainsci-12-01294-s001.zip › brainsci-1876073-supplementary-BrainSciencesFinal-suppl09252022-A.pdf]

## Supplementary Materials: Modal analysis of the physiologically detailed neurovascular model

Table S1: Natural frequencies in the physiological frequency range of 0.01-0.2 Hz obtained from the modal analysis of the four physiologically detailed tES perturbation pathways

| Pathways in columns and different runs with different seeds for the bandpass filtered white noise input in the rows | Pathway 1 | Pathway 2 | Pathway 3 | Pathway 4 |
|---|---|---|---|---|
| Run 1 | 0.0000, 0.0193 | 0.0247 | 0.0052, 0.0157, 0.0265 | 0.0000, 0.0069, 0.0201 |
| Run 2 | 0.0189, 0.0201 | 0.0003, 0.0156, 0.0167 | 0.0025, 0.0231, 0.0235 | 0.0128, 0.0141, |
| Run 3 | 0.0000, 0.0153, 0.0195 | 0.0109, 0.0191, 0.0288, 0.0476 | 0.0027, 0.0081, 0.0196, 0.0236 | 0.0067, 0.0090, 0.0144 |
| Run 4 | 0.0115, 0.0245 | NaN | 0.0050 | 0.0020, 0.0168 |
| Run 5 | 0.0007, 0.0077, 0.0200, 0.0276 | 0.0131 | 0.0005, 0.0128, 0.0176 | 0.0192, 0.0318 |
| Run 6 | 0.0179, 0.0363 | 0.0237, 0.0418 | 0.0055 | 0.0104, 0.0171 |
| Run 7 | 0.0107, 0.0131 | 0.0128, 0.0200, 0.0380, 0.0659 | 0.0036<br>0.0386 | 0.0381 |
| Run 8 | 0.0225, 0.0298 | 0.0048, 0.0100 | 0.0011, 0.0209 | 0.0043, 0.0223 |
| Run 9 | 0.0000, 0.0215 | 0.0038, 0.0285 | 0.0033, 0.0083, 0.0184 | 0.0099, 0.0164 |
| Run 10 | 0.0198, 0.0319, 0.0360 | 0.0189, 0.0229, 0.0242 | 0.0058, | 0.0387 |

| | | | | |
|---|---|---|---|---|
| | | | 0.0188 | |

Table S2: List of the poles damping parameters associated with linearized model for the four tES perturbation pathways

| Pathway | Poles | Damping | Frequency (rad/seconds) | Time constant (seconds) |
|---|---|---|---|---|
| 1 | -2.45e-01,<br>-4.00e-01,<br>-1.00e+00,<br>-1.97e+00,<br>-3.30e+00,<br>-4.90e+00 + 8.44e+00i,<br>-4.90e+00 - 8.44e+00i,<br>-1.51e+01,<br>-2.07e+01,<br>-2.97e+04,<br>-9.59e+06 | 1.00e+00,<br>1.00e+00,<br>1.00e+00,<br>1.00e+00,<br>1.00e+00,<br>5.02e-01,<br>5.02e-01,<br>1.00e+00,<br>1.00e+00,<br>1.00e+00,<br>1.00e+00, | 2.45e-01,<br>4.00e-01,<br>1.00e+00,<br>1.97e+00,<br>3.30e+00,<br>9.76e+00,<br>9.76e+00,<br>1.51e+01,<br>2.07e+01,<br>2.97e+04,<br>9.59e+06 | 4.09e+00,<br>2.50e+00,<br>1.00e+00,<br>5.09e-01,<br>3.03e-01,<br>2.04e-01,<br>2.04e-01,<br>6.63e-02,<br>4.83e-02,<br>3.36e-05,<br>1.04e-07 |
| 2 | -2.45e-01,<br>-1.00e+00,<br>-1.97e+00,<br>-3.30e+00,<br>-4.90e+00 + 8.44e+00i,<br>-4.90e+00 - 8.44e+00i,<br>-1.51e+01,<br>-2.07e+01,<br>-2.97e+04,<br>-9.59e+06 | 1.00e+00,<br>1.00e+00,<br>1.00e+00,<br>1.00e+00,<br>5.02e-01,<br>5.02e-01,<br>1.00e+00,<br>1.00e+00,<br>1.00e+00,<br>1.00e+00, | 2.45e-01,<br>1.00e+00,<br>1.97e+00,<br>3.30e+00,<br>9.76e+00,<br>9.76e+00,<br>1.51e+01,<br>2.07e+01,<br>2.97e+04,<br>9.59e+06 | 4.09e+00,<br>1.00e+00,<br>5.09e-01,<br>3.03e-01,<br>2.04e-01,<br>2.04e-01,<br>6.63e-02,<br>4.83e-02,<br>3.36e-05,<br>1.04e-07 |

| | | | | |
|---|---|---|---|---|
| 3 | -2.45e-01,<br>-1.00e+00,<br>-3.30e+00,<br>-4.90e+00 + 8.44e+00i,<br>-4.90e+00 - 8.44e+00i,<br>-2.07e+01,<br>-2.97e+04,<br>-9.59e+06 | 1.00e+00,<br>1.00e+00,<br>1.00e+00,<br>5.02e-01,<br>5.02e-01,<br>1.00e+00,<br>1.00e+00,<br>1.00e+00, | 2.45e-01,<br>1.00e+00,<br>3.30e+00,<br>9.76e+00,<br>9.76e+00,<br>2.07e+01,<br>2.97e+04,<br>9.59e+06 | 4.09e+00,<br>1.00e+00,<br>3.03e-01,<br>2.04e-01,<br>2.04e-01,<br>4.83e-02,<br>3.36e-05,<br>1.04e-07 |
| 4 | -2.45e-01,<br>-3.30e+00,<br>-4.90e+00 + 8.44e+00i,<br>-4.90e+00 - 8.44e+00i,<br>-2.07e+01,<br>-9.59e+06 | 1.00e+00,<br>1.00e+00,<br>5.02e-01,<br>5.02e-01,<br>1.00e+00,<br>1.00e+00, | 2.45e-01,<br>3.30e+00,<br>9.76e+00,<br>9.76e+00,<br>2.07e+01,<br>9.59e+06 | 4.09e+00,<br>3.03e-01,<br>2.04e-01,<br>2.04e-01,<br>4.83e-02,<br>1.04e-07 |
