# Supplementary material for: Human-in-the-Loop Optimization of Transcranial Electrical Stimulation at the Point of Care: A Computational Perspective
Source: Brain Sci. 2022 Sep 26;12(10):1294. doi: 10.3390/brainsci12101294 (PMC9599464; doi:10.3390/brainsci12101294)
Supplement: Supplementary file 1 [file brainsci-12-01294-s001.zip › brainsci-1876073-supplementary-BrainSciencesFinal-suppl09252022-B.pdf]

Supplementary Materials: Grey-box modeling of fNIRS of tDCS effects – a chronic stroke case series

Pathway Equations (from (Arora et al., 2021))

(a) Pathway 1: Synaptic Potassium → vessel circumference

In equation (1):

$$\frac{d[K^+]_s}{dt} = J_{K_s} - J_{\Sigma Kmax} k_{Na} \frac{[K^+]_s}{[K^+]_s + KKO_a}$$

J_{K_s} which is the potassium release from active neurons is considered to be modulated by transcranial direct current stimulation (tDCS) and given as input to the model and equations were solved.

(b) Pathway 2: Astrocytic Current channel → vessel circumference

In equation (21): additional current term corresponding to tDCS (I_T) has been assumed which is a function of applied tDCS pulse

$$\frac{dV_k}{dt} = \frac{1}{C_{astr}} \left(- I_{BK} - I_{leak} - I_{TRPV} - I_{\Sigma K} + I_T\right)$$

(c) Pathway 3: Perivascular Potassium → vessel circumference

In equation (26): additional potassium concentration corresponding to tDCS ($[K^+]_T$) has been assumed which is a function of applied tDCS pulse

$$\frac{d[K^+]_P}{dt} = \frac{J_{BK}}{VR_{pa}} + \frac{J_{KIR}}{VR_{ps}} - R_{decay}\left([K^+]_P - [K^+]_{P,min}\right) + [K^+]_T$$

(d) Pathway 4: Voltage gated ion channel on SMC → vessel circumference

In equation (41): additional voltage gated current channel (I_{KV}) that gets affected by tDCS is taken in consideration for equation for SMC's membrane potential

$$\frac{dV_m}{dt} = \frac{1}{C_{SMC}} \left(-I_L - I_K - I_{Ca} - I_{KIR} - I_{KV}\right)$$

While solving the model, for four pathways, J_{K_s}, I_T, $[K^+]_T$ and, I_{KV} were taken as first order transfer function of tDCS input pulse.

Table S1. Transfer functions were obtained through linear analysis tool (Mathworks, Inc., USA) for the four NVU model pathways.

NVU pathways	Transfer function from vasoactive signal to fNIRS HbT changes	Parametrization
1. Synaptic Potassium → vessel circumference	(s+2.371e07)(s+46.5)(s+2.962)/ (s+9.594e06)(s+2.974e04)(s+20.69) (s+15.08)(s+3.3)(s+1.966)(s+1) (s+0.4)(s+0.2446)(s^2 + 9.804s + 95.24)	11 poles, 3 zeros
2. Astrocytic membrane potential → vessel circumference	(s+2.371e07)(s+46.5)(s+2.962)/ (s+2.974e04)(s+20.69)(s+15.08) (s+3.3) (s+1.966)(s+1)(s+0.2446)(s+9.594e06) (s^2 + 9.804s + 95.24)	10 poles, 3 zeros
3. Perivascular Potassium → vessel circumference	(s+2.371e07)(s+2.962)/ (s+9.594e06)(s+2.974e04)(s+20.69) (s+3.3) (s+1)(s+0.2446)(s^2 + 9.804s + 95.24)	8 poles, 2 zeros
4. Voltage gated ion channel on SMC → vessel circumference	(s+2.962)/ (s+9.594e06)(s+20.69) (s+3.3) (s+0.2446)(s^2 + 9.804s + 95.24)	6 poles, 1 zero

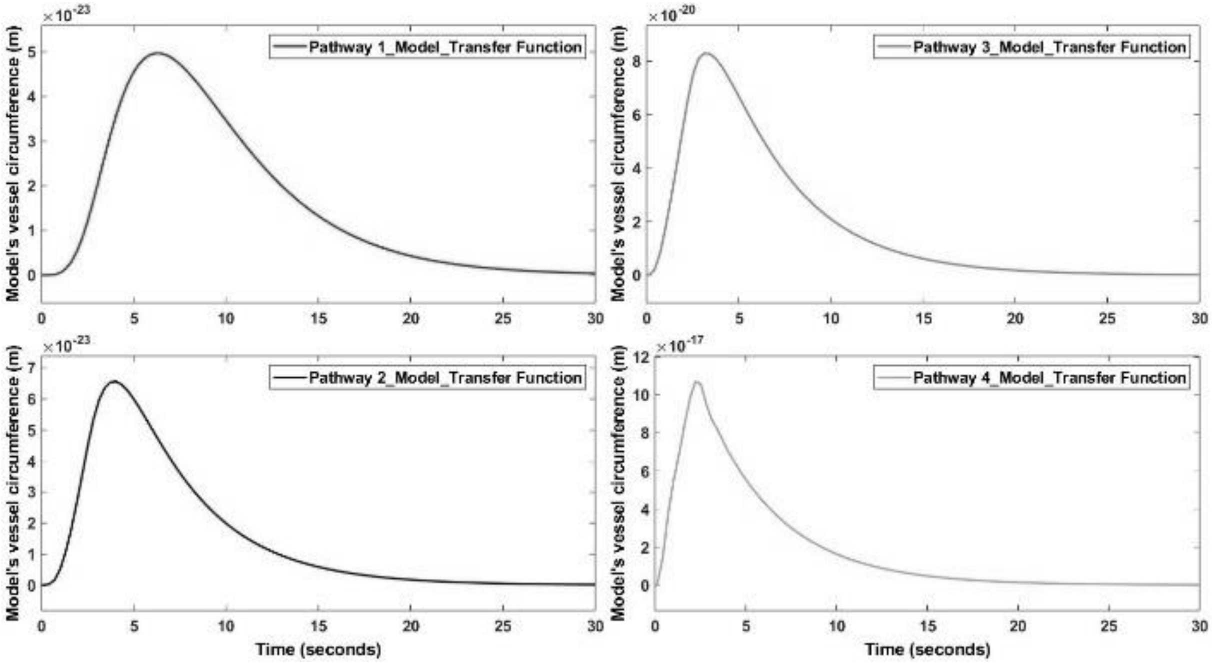

Figure S1: Model output vessel response (vessel circumference) to the input square tDCS test pulse (2s duration, amplitude 2mA, starting at time zero) is shown for the four pathways (transger functions of the pathways listed in Table S1 above)
