# Supplementary material for: Human-in-the-Loop Optimization of Transcranial Electrical Stimulation at the Point of Care: A Computational Perspective
Source: Brain Sci. 2022 Sep 26;12(10):1294. doi: 10.3390/brainsci12101294 (PMC9599464; doi:10.3390/brainsci12101294)
Supplement: Supplementary file 1 [file brainsci-12-01294-s001.zip › brainsci-1876073-supplementary-BrainSciencesFinal-suppl09252022-C.pdf]

Supplementary Materials: Black box modeling of prefrontal fNIRS-pupillometry of short-duration frontal tDCS effects – a healthy case series

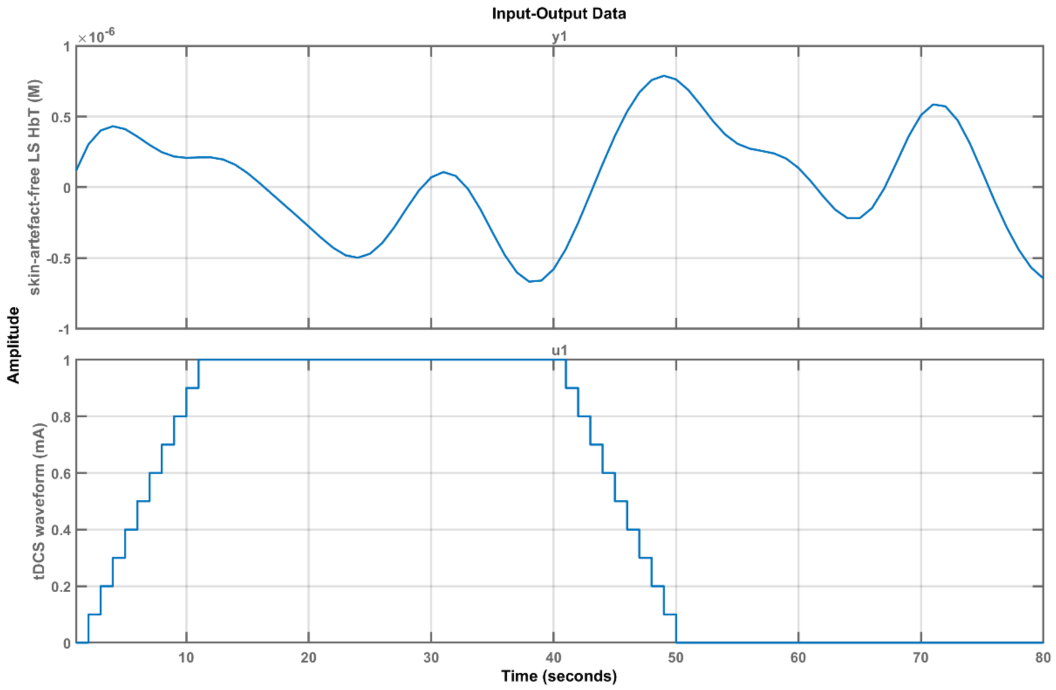

Figure S1: An illustrative plot of the skin-artefact-free long separation (LS) total hemoglobin changes (HbT) evoked by the tDCS ON waveform (mA).

1. The model from tDCS waveform input to LS HbT output (tDCS=>LS) was initialized with reduced-dimension model (8 poles, 2 zeros (Arora et al., 2021c)) for Pathway 3: Perivascular Potassium → vessel circumference.

tDCS=>LS HbT

-8.334e-11 s^2 + 2.995e-11 s + 4.422e-12

----

s^8 + 0.2545 s^7 + 0.3353 s^6 + 0.07136 s^5 + 0.03394 s^4 + 0.005443 s^3 + 0.001251 s^2 + 0.000122 s + 1.216e-05

Continuous-time identified transfer function.

Parameterization:

Number of poles: 8   Number of zeros: 2

Number of free coefficients: 11

Status:

Estimated using TFEST on time domain data.

Fit to estimation data: 54.55% (stability enforced)

FPE: 8.843e-13, MSE: 5.449e-13

2. The model from tDCS waveform input to SS HbT output (tDCS=>SS) was initialized with reduced-dimension model (6 poles, 1 zeros (Arora et al., 2021c)) for Pathway 4: Smooth muscle cell → vessel circumference.

tDCS=>SS HbT

-8.334e-11 s^2 + 2.995e-11 s + 4.422e-12

----

s^8 + 0.2545 s^7 + 0.3353 s^6 + 0.07136 s^5 + 0.03394 s^4 + 0.005443 s^3 + 0.001251 s^2 + 0.000122 s + 1.216e-05

Continuous-time identified transfer function.

Parameterization:

Number of poles: 8   Number of zeros: 2

Number of free coefficients: 11

Status:

Estimated using TFEST on time domain data.

Fit to estimation data: 54.55% (stability enforced)

FPE: 8.843e-13, MSE: 5.449e-13

3. The model from SS HbT input to LS HbT output (SS=>LS) was a static gain model.

SS HbT=>LS HbT

2.361

Static gain.

Parameterization:

Number of poles: 0   Number of zeros: 0

Number of free coefficients: 1

Status:

Estimated using TFEST on time domain data.

Fit to estimation data: 76.61% (stability enforced)

FPE: 1.479e-13, MSE: 1.443e-13

4. The model from tDCS waveform input to skin-artefact-free LS HbT output (LS – (SS=>LS)) was initialized with reduced-dimension model (8 poles, 2 zeros (Arora et al., 2021c)) for Pathway 3: Perivascular Potassium → vessel circumference.

LS HbT – (SS HbT=>LS HbT)

2.022e-10 s^2 + 7.678e-12 s + 1.374e-13

----

s^8 + 0.4073 s^7 + 0.4445 s^6 + 0.128 s^5 + 0.05998 s^4 + 0.01001 s^3 + 0.002776 s^2 + 0.000161 s + 2.749e-05

Continuous-time identified transfer function.

Parameterization:

Number of poles: 8   Number of zeros: 2

Number of free coefficients: 11

Status:

Estimated using TFEST on time domain data "dataLSminusSS".

Fit to estimation data: 71.14% (stability enforced)

FPE: 1.915e-14, MSE: 1.18e-14
