# Supplementary material for: Human-in-the-Loop Optimization of Transcranial Electrical Stimulation at the Point of Care: A Computational Perspective
Source: Brain Sci. 2022 Sep 26;12(10):1294. doi: 10.3390/brainsci12101294 (PMC9599464; doi:10.3390/brainsci12101294)
Supplement: Supplementary file 1 [file brainsci-12-01294-s001.zip › brainsci-1876073-supplementary-BrainSciencesFinal-suppl09252022-D.pdf]

Supplementary Materials: Human-in-the-loop optimization for model predictive control of tES evoked HbT

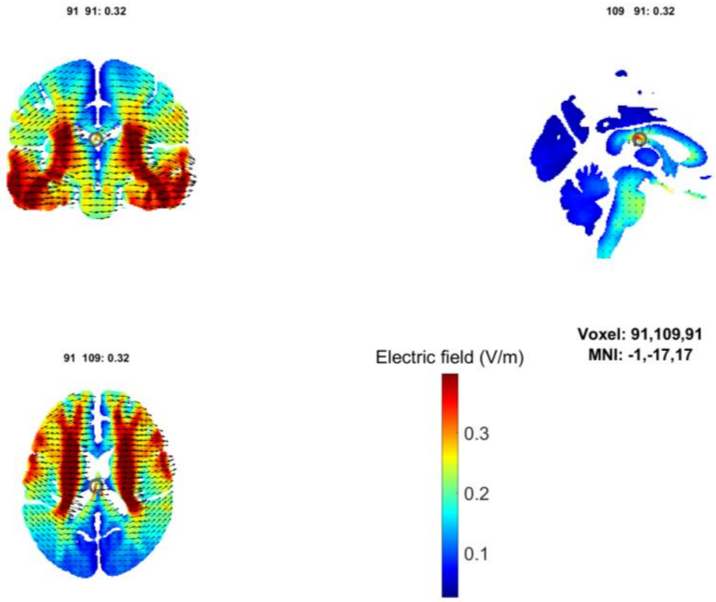

Figure S1: Electrical Field distribution for 2mA tDCS with FT7 (anodal electrode) and FT8 (cathodal electrode).

It can be assumed that the tDCS-applied electric field will linearly modify the average membrane potential (V) of the different neuronal subpopulations (within a certain range of intensity)(Molaee-Ardekani et al., 2013), i.e. $\Delta V = \lambda \cdot E$, where the electric field (E) can be modeled using anatomically realistic full-head model. Then, membrane polarization will lead to vessel response as presented in our prior work (Arora et al., 2021b). Here, tDCS current density at the scalp (I_{tdcs}) is assumed to be proportional to the current density (J_{tDCS}) in the neurovascular brain tissue based on a leadfield matrix leading to the vasoactive signal via first order transfer function, $v_i = \frac{\lambda}{s/\tau+1} I_{tdcs}$ *(equation i)*, where λ is arbitrary gain from lead field matrix and τ is the time constant (Arora et al., 2021).
