# Supplementary material for: Human-in-the-Loop Optimization of Transcranial Electrical Stimulation at the Point of Care: A Computational Perspective
Source: Brain Sci. 2022 Sep 26;12(10):1294. doi: 10.3390/brainsci12101294 (PMC9599464; doi:10.3390/brainsci12101294)
Supplement: Supplementary file 1 [file brainsci-12-01294-s001.zip › brainsci-1876073-supplementary-BrainSciencesFinal-suppl09252022-processedData.pdf]

Supplementary Materials: Black box modeling – processed subject data

SUBJECT 1:

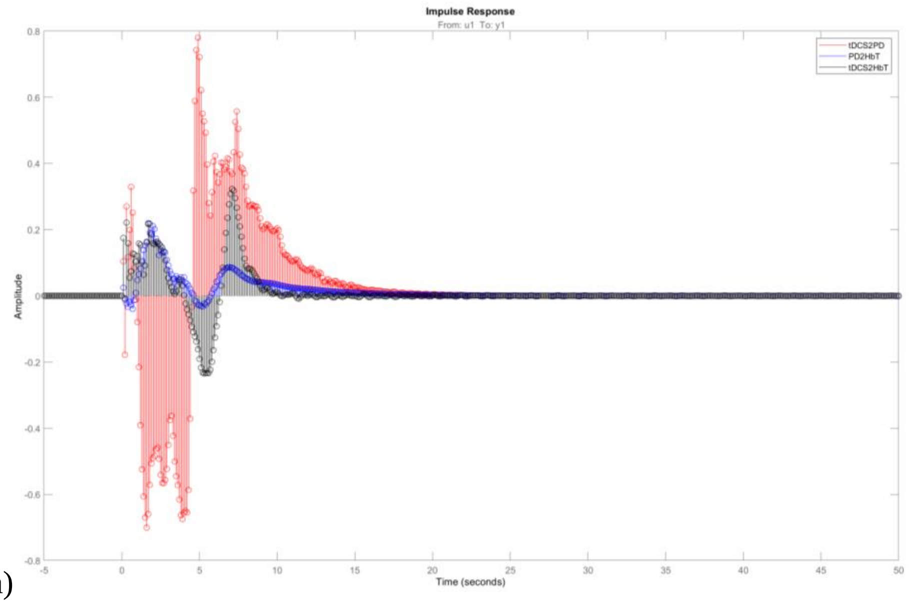

(a)

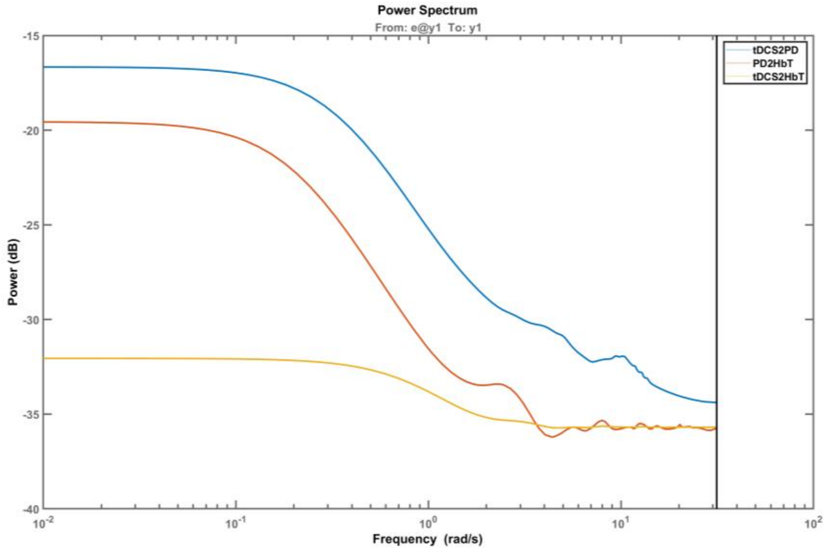

(b)

Figure S1: Subject 1 (a) impulse responses of these state-space models using regularized reduction of an ARX model. At tDCS onset (near t=0), an anti-phase relationship between tDCS-evoked pupil diameter changes (tDCS2PD line in red) and tDCS-evoked blood volume changes (tDCS2HbT line in black) can be seen. Then, the pupil diameter changes as input and the blood volume changes as output (PD2HbT line in blue) has an initial dip at tDCS onset (near t=0). (b) Power spectrum of the black box models with tDCS2PD line in blue, PD2HbT line in red, and tDCS2HbT line in yellow.

HEMODYNAMIC RESPONSE ANALYSIS

0.1. Sample Autocorrelation Function

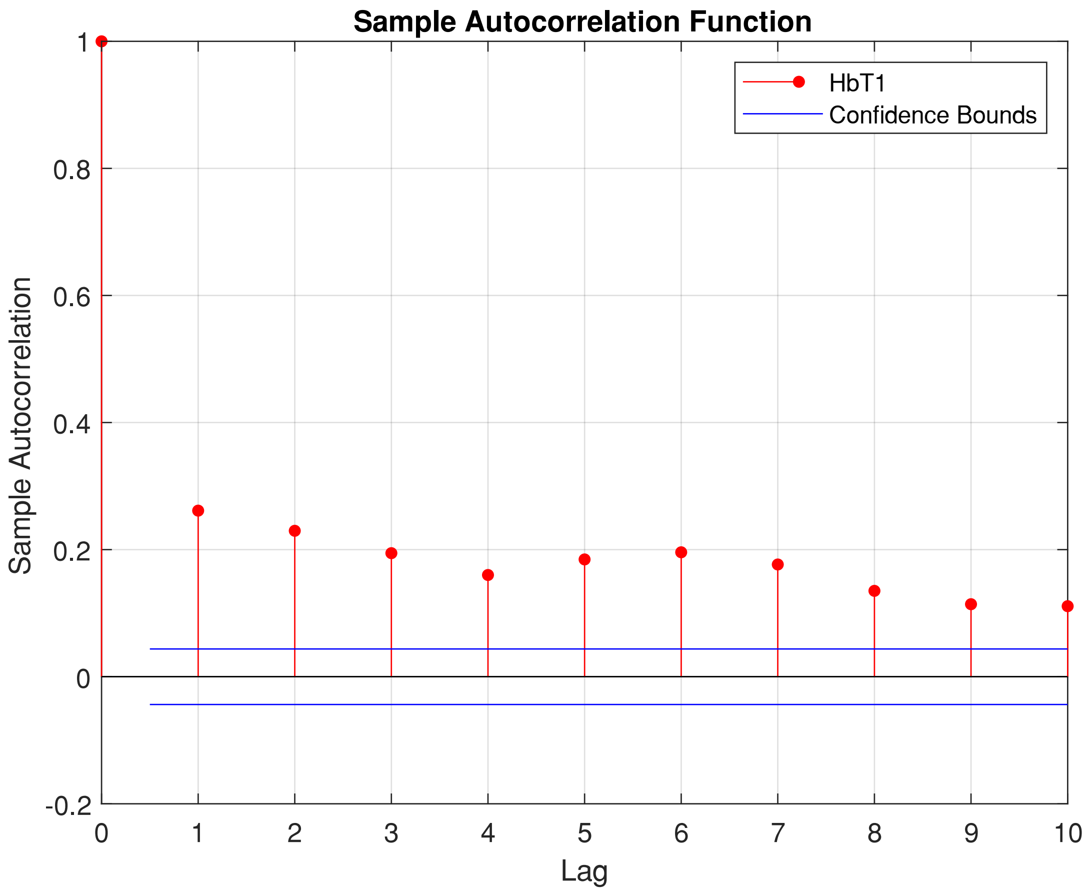

Figure S0.1. Sample autocorrelation function of HbT1

0.2. Sample Partial Autocorrelation Function

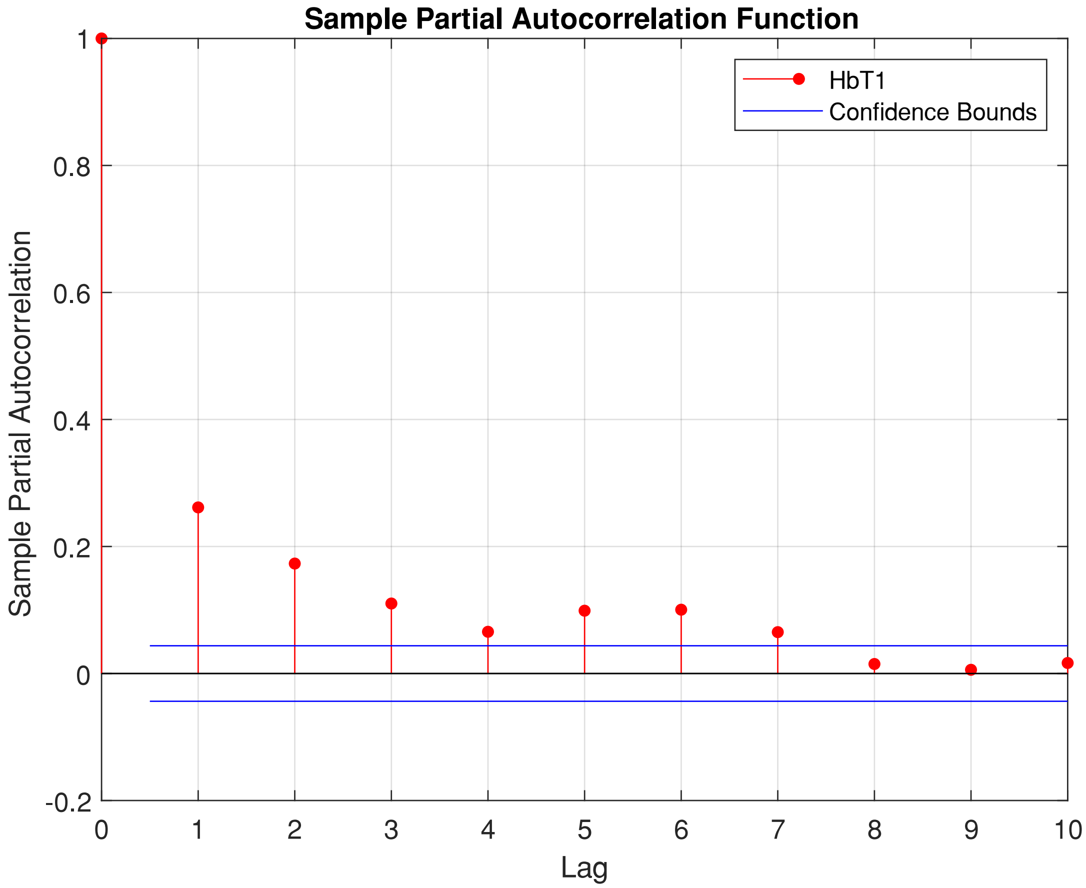

Figure S0.2. Sample partial autocorrelation function of HbT1

0.3. Augmented Dickey-Fuller Test

Null Hypothesis: HbT1 contains a unit root

$$y_t = c + \delta t + \phi y_{t-1} + \beta_1 \Delta y_{t-1} + \ldots + \beta_p \Delta y_{t-p} + \varepsilon_t$$

$$H_0 : \phi = 1$$

$$H_a : \phi < 1$$

Table S0.1. Test Parameters

	Lags	Model	Test Statistic	Significance Level
1	0	AR	t1	0.025
2	1	AR	t1	0.025

Table S0.2. Test Results

	Null Rejected	P-Value	Test Statistic	Critical Value
1	true	0.0041435	-2.9069	-2.2297
2	false	0.083913	-1.7034	-2.2297

0.4. Residual Quantile-Quantile plot

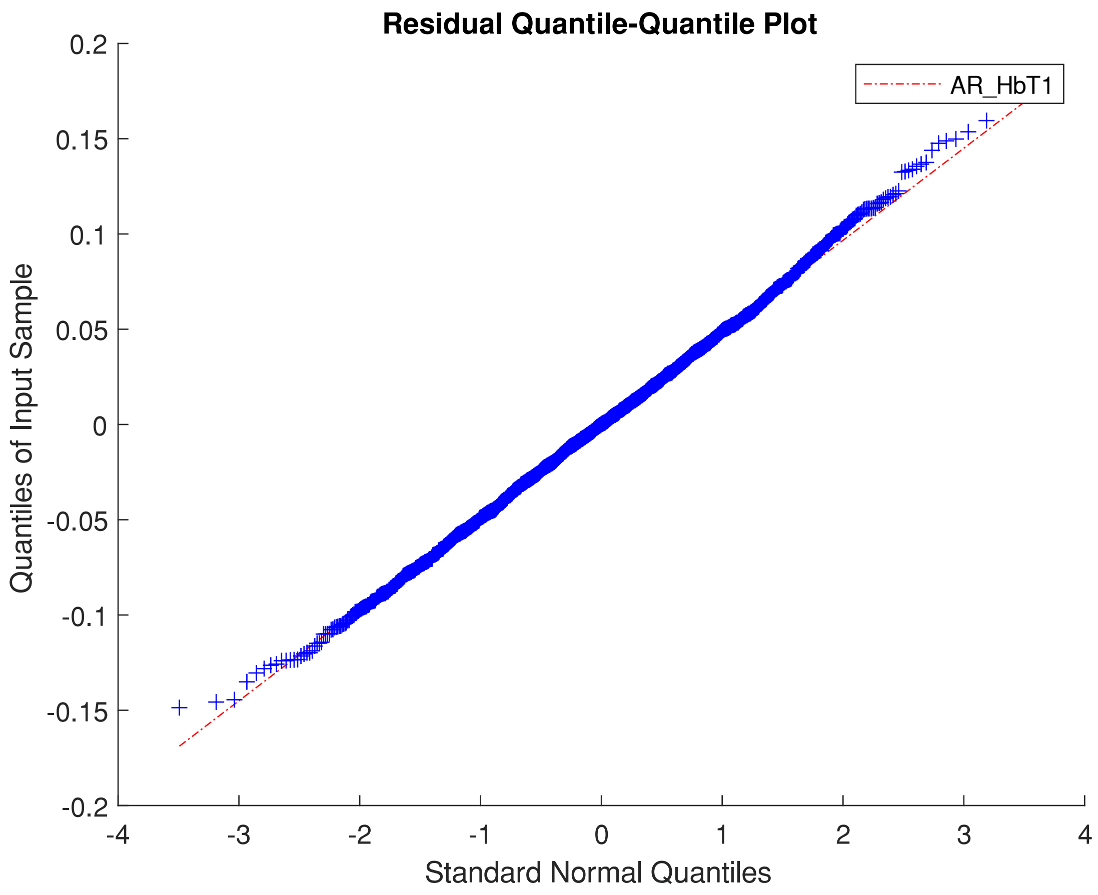

Figure S0.3. Quantile-quantile plot of the residuals of model AR_HbT1.

0.5. Residual Sample Autocorrelation Function

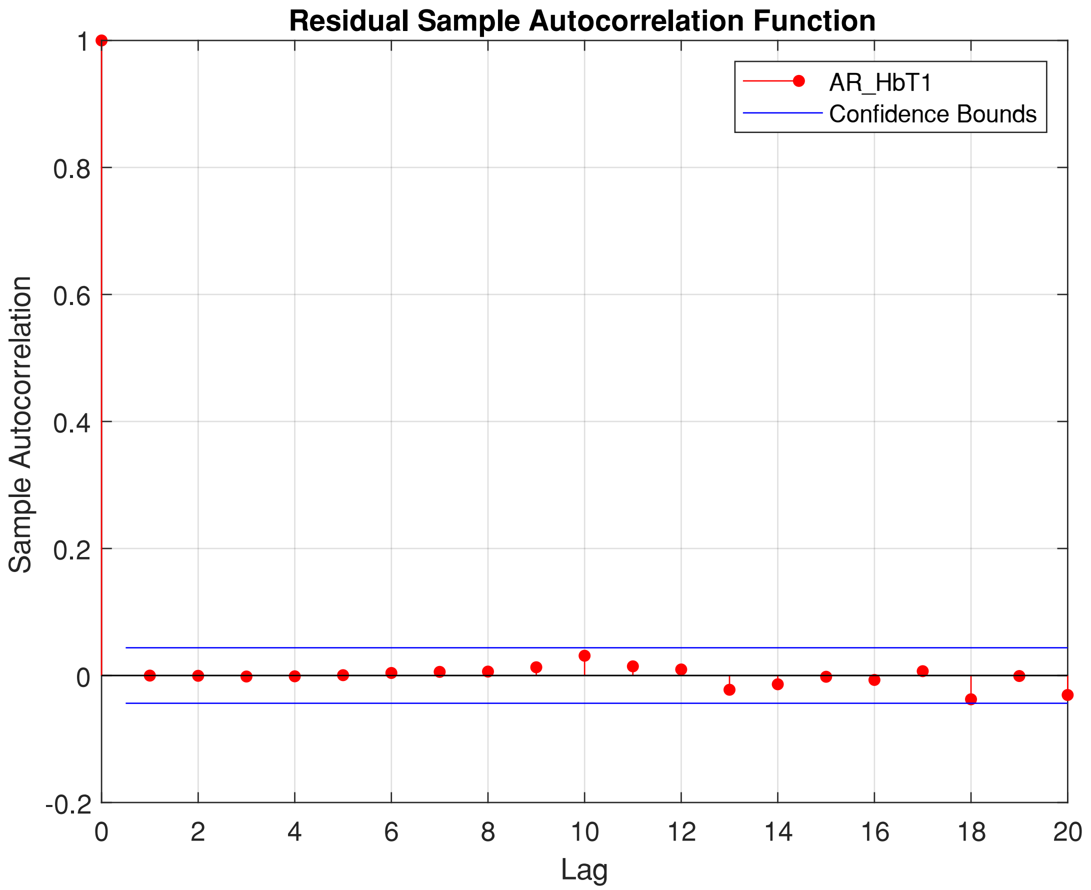

Figure S0.4. Sample autocorrelation function of the residuals of AR_HbT1

0.6. Ljung-Box Q-Test

Null Hypothesis: The first m autocorrelations of the residuals of AR_HbT1 are jointly 0

$$H_0 : \rho_1 = \rho_2 = \ldots = \rho_m = 0$$

$$H_a : \rho_j \neq 0, j \in 1, \ldots, m$$

Table S0.3. Test Parameters

	Lags	DOF	Significance Level
1	10	10	0.025

Table S0.4. Test Results

	Null Rejected	P-Value	Test Statistic	Critical Value
1	false	0.98949	2.5904	20.4832

1.ARIMA(8,0,0) Model (Gaussian Distribution) (AR_HbT1)

Autoregressive model of time series HbT1 with the following equation:

$$(1 - \phi_1 L - \ldots - \phi_8 L^8) y_t = c + \varepsilon_t$$

1.1. Model Estimation

Table S1.1. Estimation Results

Parameter	Value	Standard Error	t Statistic	P-Value
Constant	0.19233	0.018495	10.399	2.5047e-25
AR{1}	0.16617	0.021338	7.7873	6.8434e-15
AR{2}	0.11833	0.021775	5.434	5.5095e-08
AR{3}	0.072029	0.022372	3.2197	0.0012834
AR{4}	0.028312	0.022964	1.2329	0.21762
AR{5}	0.071318	0.023155	3.0801	0.0020695
AR{6}	0.087432	0.022378	3.907	9.3432e-05
AR{7}	0.062832	0.022398	2.8052	0.0050286
AR{8}	0.015075	0.022561	0.66818	0.50402
Variance	0.0024178	7.4309e-05	32.5374	3.1559e-232

Table S1.2. Goodness of Fit

AIC	-6653.6037
BIC	-6597.1737

1.2. Sample Autocorrelation Function

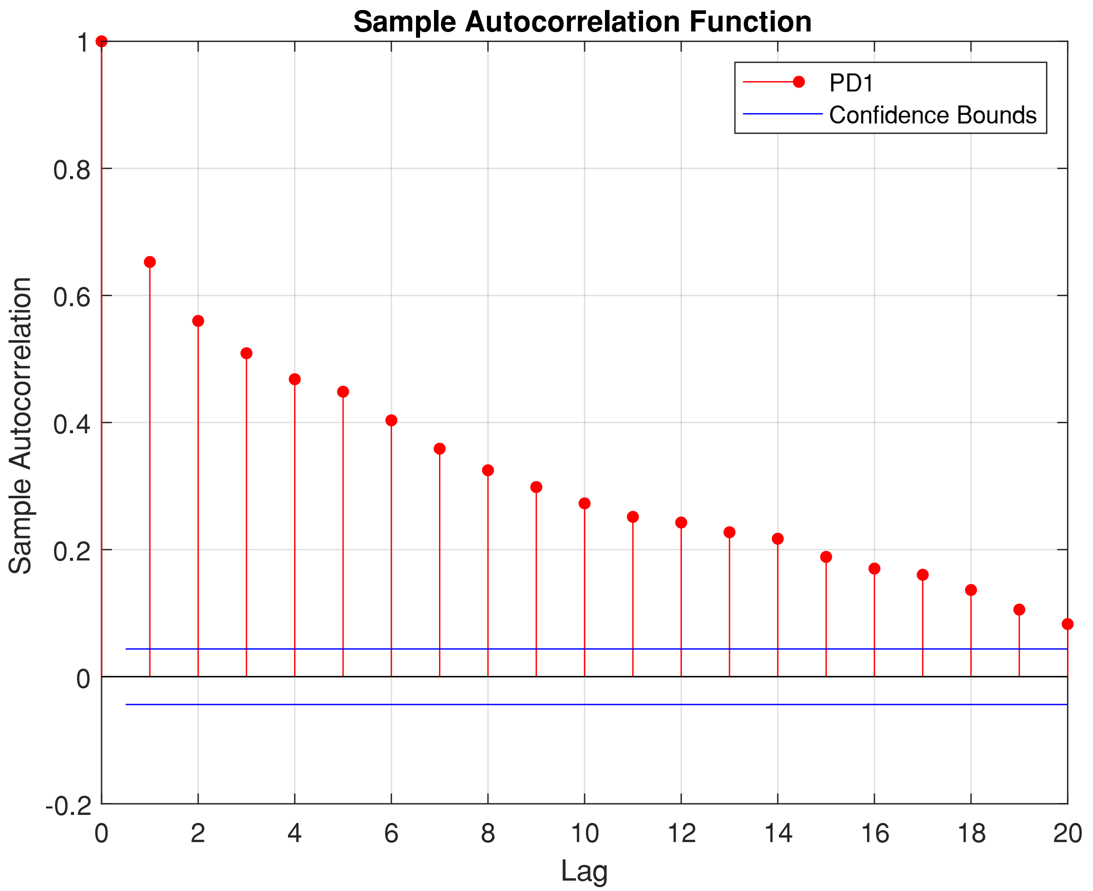

Figure S1.1. Sample autocorrelation function of PD1

1.3. Sample Partial Autocorrelation Function

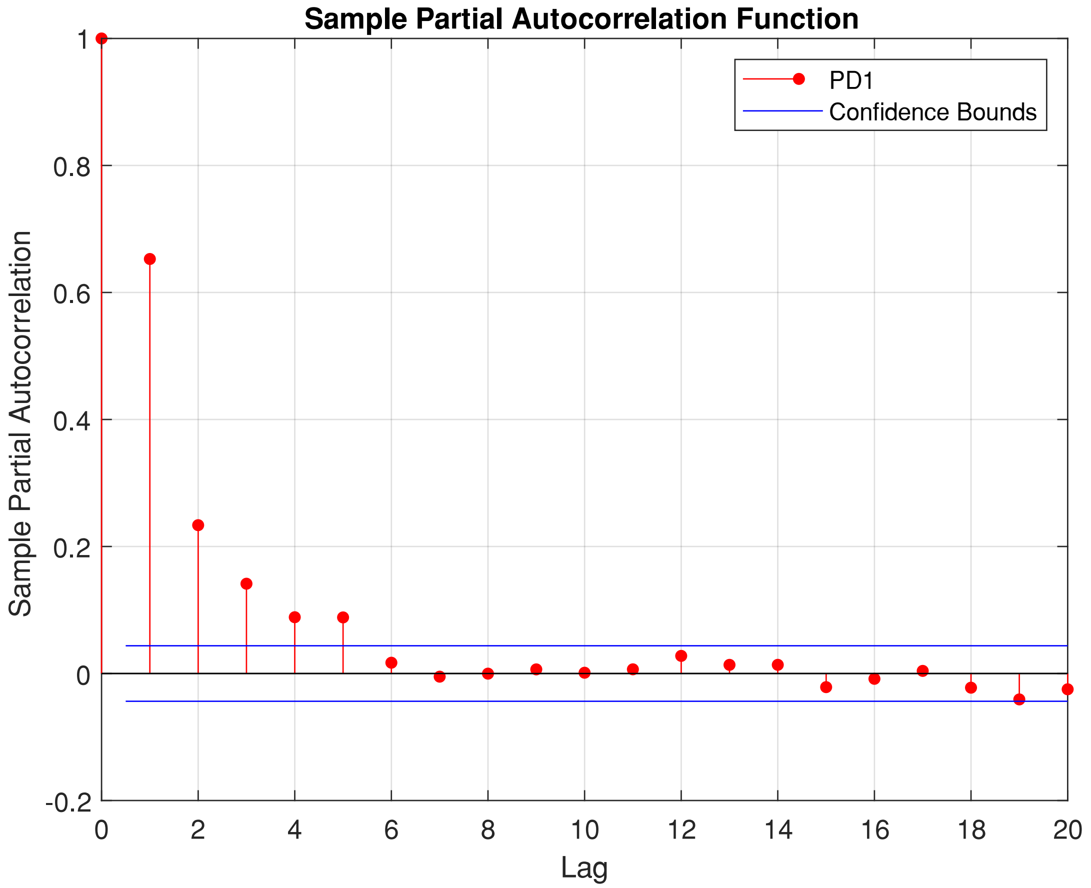

Figure S1.2. Sample partial autocorrelation function of PD1

1.4. Augmented Dickey-Fuller Test

Null Hypothesis: PD1 contains a unit root

$$y_t = c + \delta t + \phi y_{t-1} + \beta_1 \Delta y_{t-1} + \dots + \beta_p \Delta y_{t-p} + \varepsilon_t$$

$$H_0 : \phi = 1$$

$$H_a : \phi < 1$$

Table S1.3. Test Parameters

	Lags	Model	Test Statistic	Significance Level
1	0	AR	t1	0.025
2	1	AR	t1	0.025

Table S1.4. Test Results

	Null Rejected	P-Value	Test Statistic	Critical Value
1	true	0.0091633	-2.6081	-2.2297
2	false	0.069677	-1.7917	-2.2297

2.ARIMA(6,0,0) Model (Gaussian Distribution) (AR_PD1)

Autoregressive model of time series PD1 with the following equation:

$$(1 - \phi_1 L - \ldots - \phi_6 L^6) y_t = c + \varepsilon_t$$

2.1. Model Estimation

Table S2.1. Estimation Results

Parameter	Value	Standard Error	t Statistic	P-Value
Constant	0.13812	0.017161	8.0486	8.3779e-16
AR{1}	0.4453	0.010902	40.8451	0
AR{2}	0.13881	0.015567	8.9168	4.8005e-19
AR{3}	0.085505	0.023947	3.5706	0.00035622
AR{4}	0.046212	0.029329	1.5757	0.1151
AR{5}	0.080899	0.020683	3.9114	9.1764e-05
AR{6}	0.016995	0.031016	0.54795	0.58373
Variance	0.005413	7.7163e-05	70.1494	0

Table S2.2. Goodness of Fit

AIC	-4969.981
BIC	-4924.8293

2.2. Residual Quantile-Quantile plot

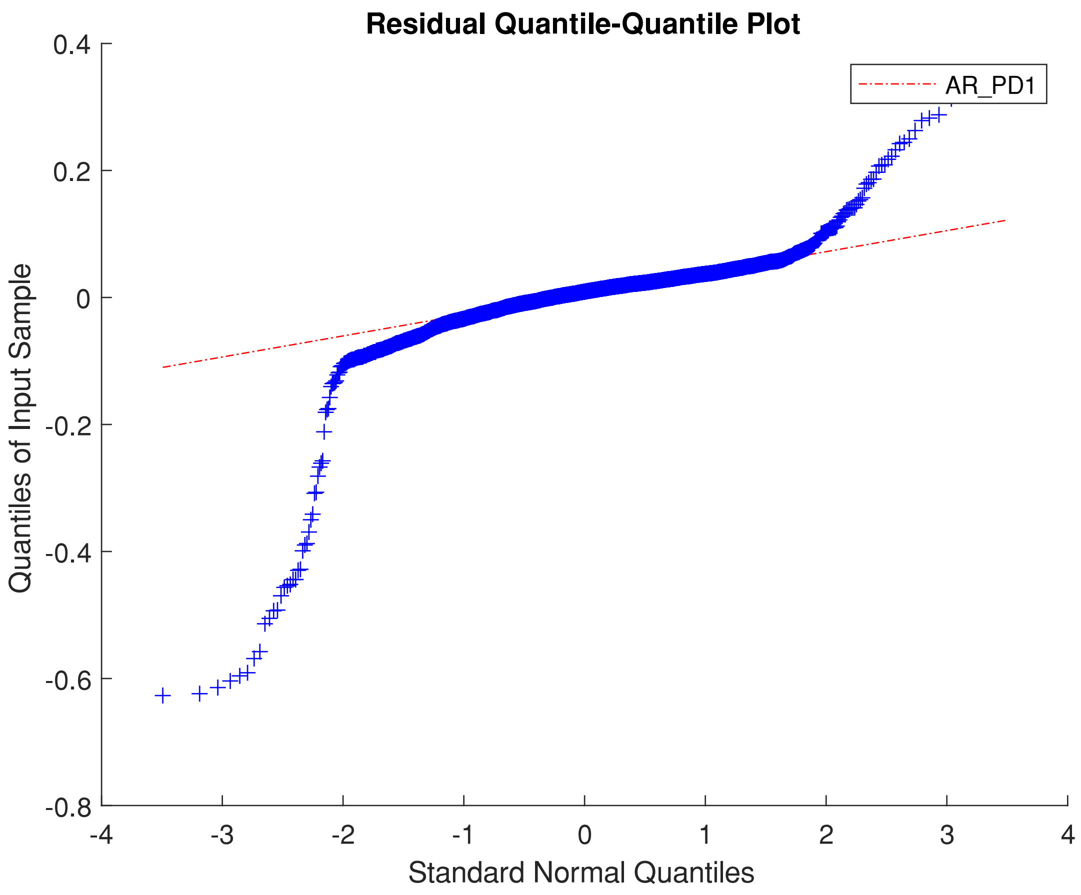

Figure S2.1. Quantile-quantile plot of the residuals of model AR_PD1.

2.3. Residual Sample Autocorrelation Function

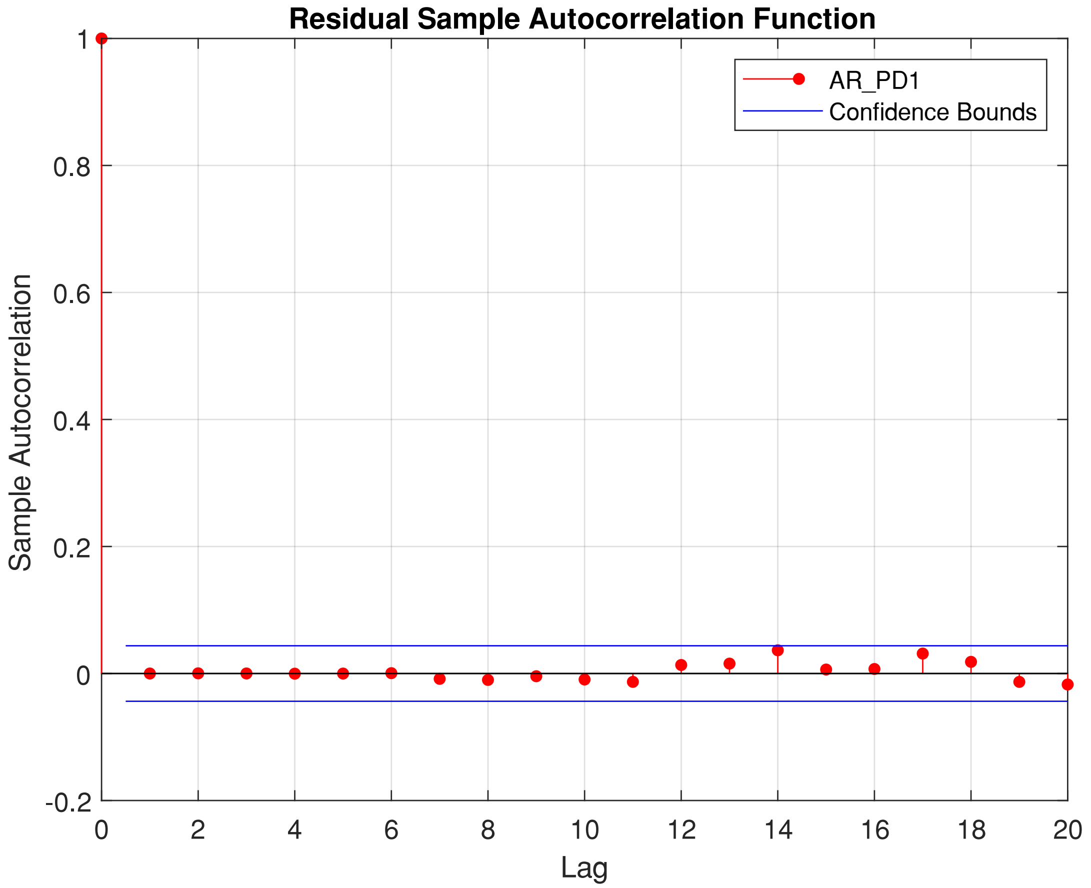

Figure S2.2. Sample autocorrelation function of the residuals of AR_PD1

2.4. Ljung-Box Q-Test

Null Hypothesis: The first m autocorrelations of the residuals of AR_PD1 are jointly 0

$$H_0 : \rho_1 = \rho_2 = \ldots = \rho_m = 0$$

$$H_a : \rho_j \neq 0, j \in 1, \ldots, m$$

Table S2.3. Test Parameters

	Lags	DOF	Significance Level
1	10	10	0.025

Table S2.4. Test Results

	Null Rejected	P-Value	Test Statistic	Critical Value
1	false	0.99999	0.59043	20.4832

3. GCtest 'blockwise'

H0	Decision	Distribution	Statistic	PValue	CriticalValue
"Exclude lagged tDCS in HbT,PD equations"	"Reject H0"	"F(50,1988)"	1.3635	0.047238	1.3567

H0	Decision	Distribution	Statistic	PValue	CriticalValue
"Exclude lagged tDCS in HbT equations"	"Cannot reject H0"	"F(25,1988)"	1.1612	0.26444	1.5116

H0	Decision	Distribution	Statistic	PValue	CriticalValue
"Exclude lagged tDCS in PD equations"	"Reject H0"	"F(25,1988)"	1.5654	0.037029	1.5116

SUBJECT 2:

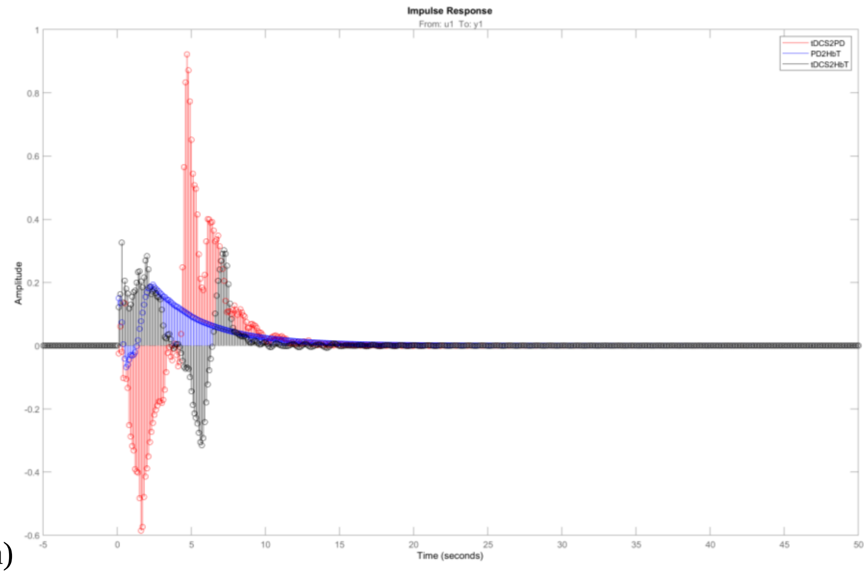

(a)

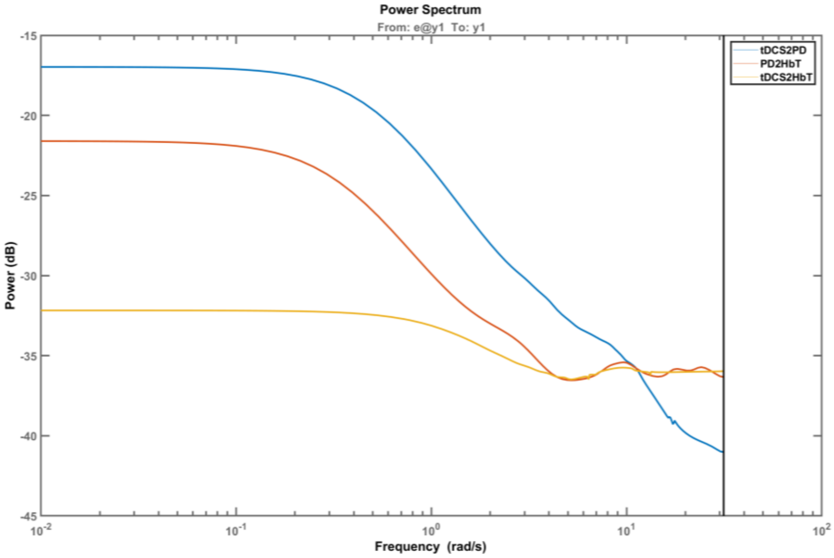

(b)

Figure S2: Subject 2 (a) impulse responses of these state-space models using regularized reduction of an ARX model. At tDCS onset (near t=0), an anti-phase relationship between tDCS-evoked pupil diameter changes (tDCS2PD line in red) and tDCS-evoked blood volume changes (tDCS2HbT line in black) can be seen. Then, the pupil diameter changes as input and the blood volume changes as output (PD2HbT line in blue) has an initial dip at tDCS onset (near t=0). (b) Spectrum of the black box models with tDCS2PD line in blue, PD2HbT line in red, and tDCS2HbT line in yellow.

2.5. Sample Autocorrelation Function

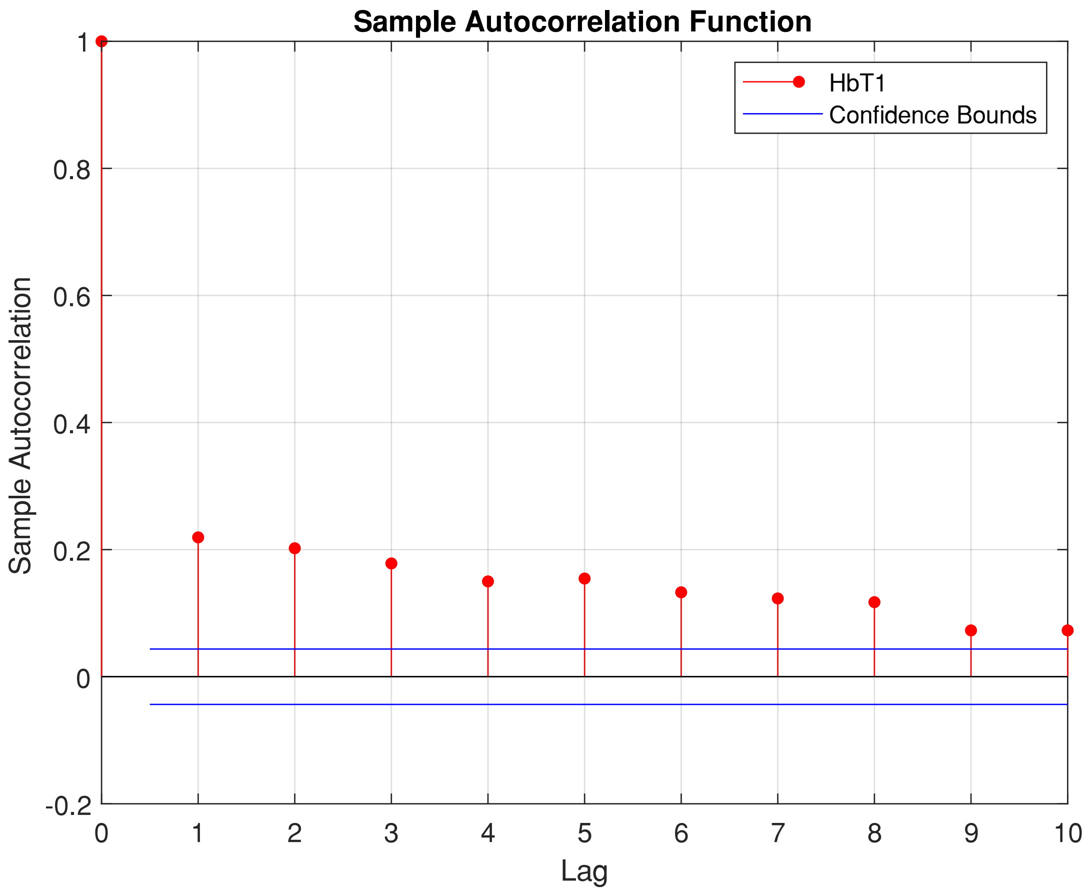

Figure S2.3. Sample autocorrelation function of HbT1

2.6. Sample Partial Autocorrelation Function

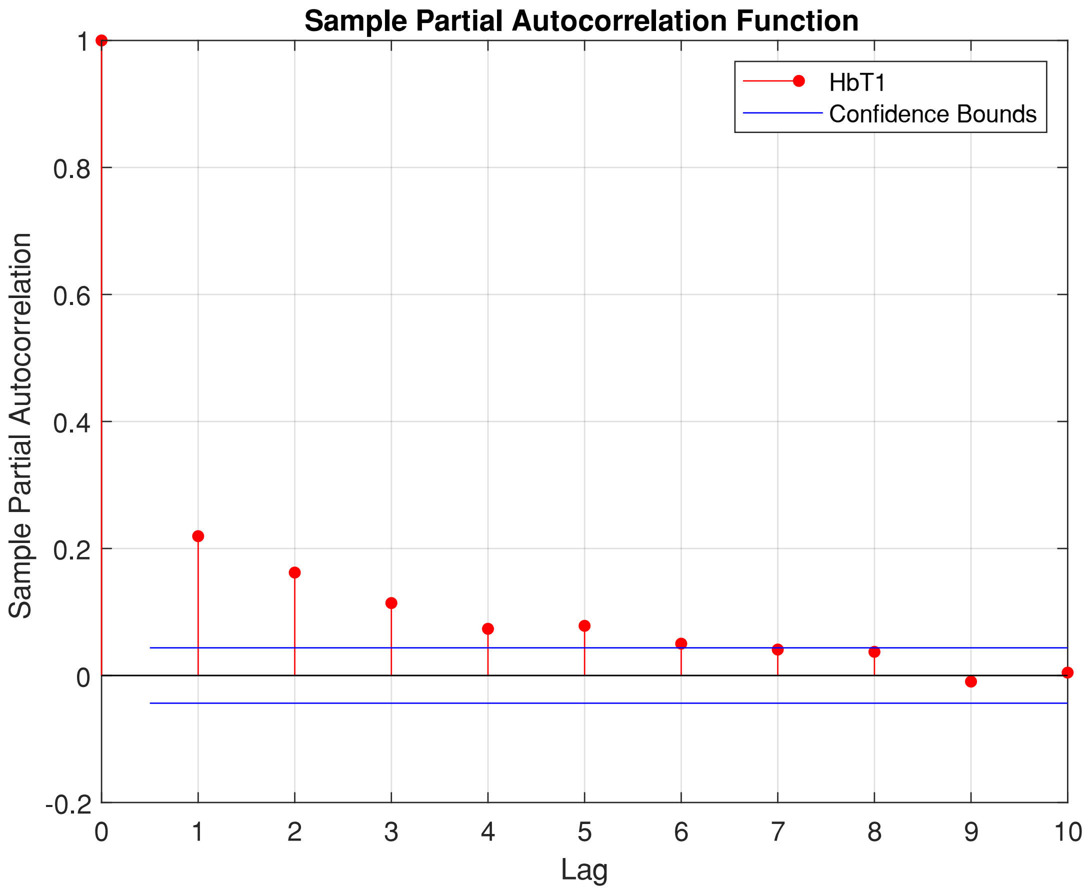

Figure S2.4. Sample partial autocorrelation function of HbT1

2.7. Augmented Dickey-Fuller Test

Null Hypothesis: HbT1 contains a unit root

$$y_t = c + \delta t + \phi y_{t-1} + \beta_1 \Delta y_{t-1} + \ldots + \beta_p \Delta y_{t-p} + \varepsilon_t$$

$$H_0 : \phi = 1$$

$$H_a : \phi < 1$$

Table S2.5. Test Parameters

	Lags	Model	Test Statistic	Significance Level
1	0	AR	t1	0.025
2	1	AR	t1	0.025

Table S2.6. Test Results

	Null Rejected	P-Value	Test Statistic	Critical Value
1	true	0.0037126	-2.9593	-2.2297
2	false	0.076516	-1.7476	-2.2297

3.ARIMA(8,0,0) Model (Gaussian Distribution) (AR_HbT1)

Autoregressive model of time series HbT1 with the following equation:

$$(1 - \phi_1 L - \ldots - \phi_8 L^8) y_t = c + \varepsilon_t$$

3.1. Model Estimation

Table S3.1. Estimation Results

Parameter	Value	Standard Error	t Statistic	P-Value
Constant	0.23456	0.020836	11.2573	2.1328e-29
AR{1}	0.14382	0.021526	6.6814	2.3661e-11
AR{2}	0.11524	0.022208	5.1891	2.1136e-07
AR{3}	0.082407	0.021828	3.7753	0.00015984
AR{4}	0.049584	0.022605	2.1935	0.028269
AR{5}	0.062688	0.022536	2.7817	0.0054075
AR{6}	0.039797	0.021485	1.8523	0.063981
AR{7}	0.035496	0.021959	1.6164	0.106
AR{8}	0.037428	0.022535	1.6609	0.096735
Variance	0.0027903	9.0416e-05	30.8607	4.0249e-209

Table S3.2. Goodness of Fit

AIC	-6390.0801
BIC	-6333.5927

3.2. Residual Quantile-Quantile plot

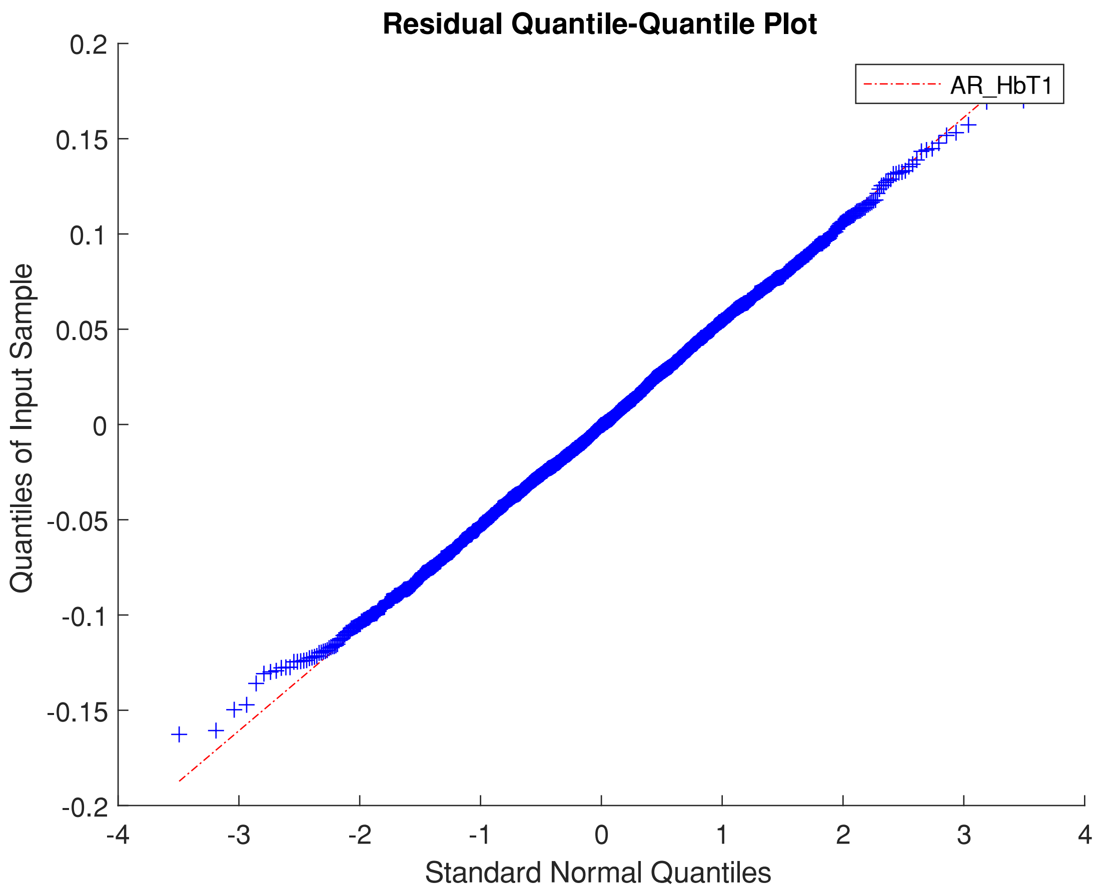

Figure S3.1. Quantile-quantile plot of the residuals of model AR_HbT1.

3.3. Residual Sample Autocorrelation Function

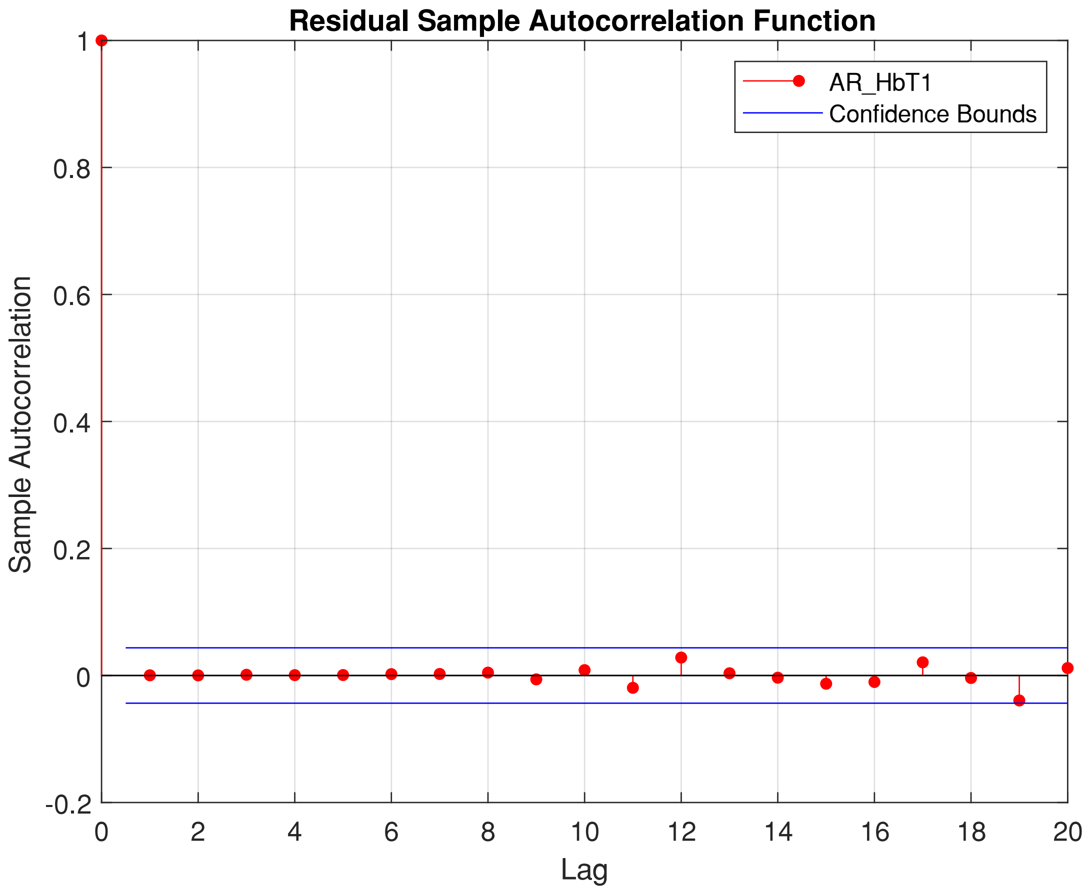

Figure S3.2. Sample autocorrelation function of the residuals of AR_HbT1

3.4. Ljung-Box Q-Test

Null Hypothesis: The first m autocorrelations of the residuals of AR_HbT1 are jointly 0

$$H_0 : \rho_1 = \rho_2 = \ldots = \rho_m = 0$$

$$H_a : \rho_j \neq 0,\ j \in 1, \ldots, m$$

Table S3.3. Test Parameters

	Lags	DOF	Significance Level
1	10	10	0.025

Table S3.4. Test Results

	Null Rejected	P-Value	Test Statistic	Critical Value
1	false	1	0.30188	20.4832

3.5. Sample Autocorrelation Function

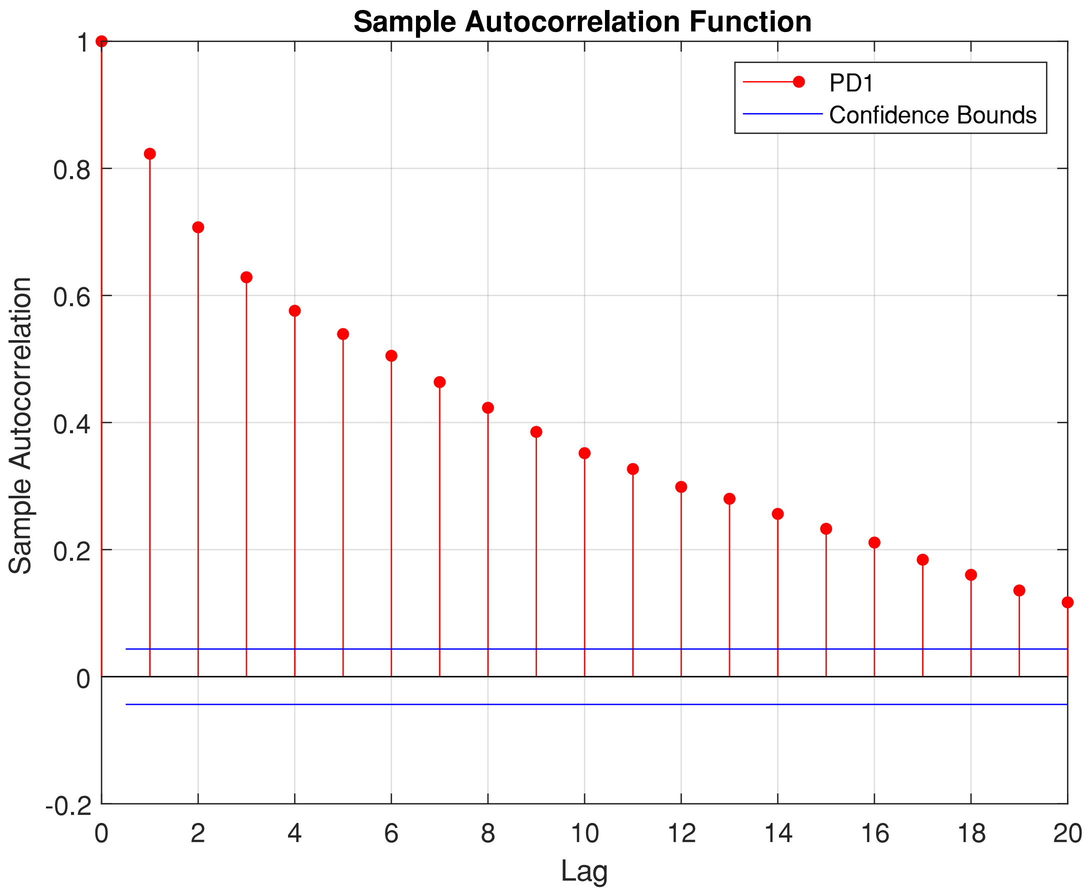

Figure S3.3. Sample autocorrelation function of PD1

3.6. Sample Partial Autocorrelation Function

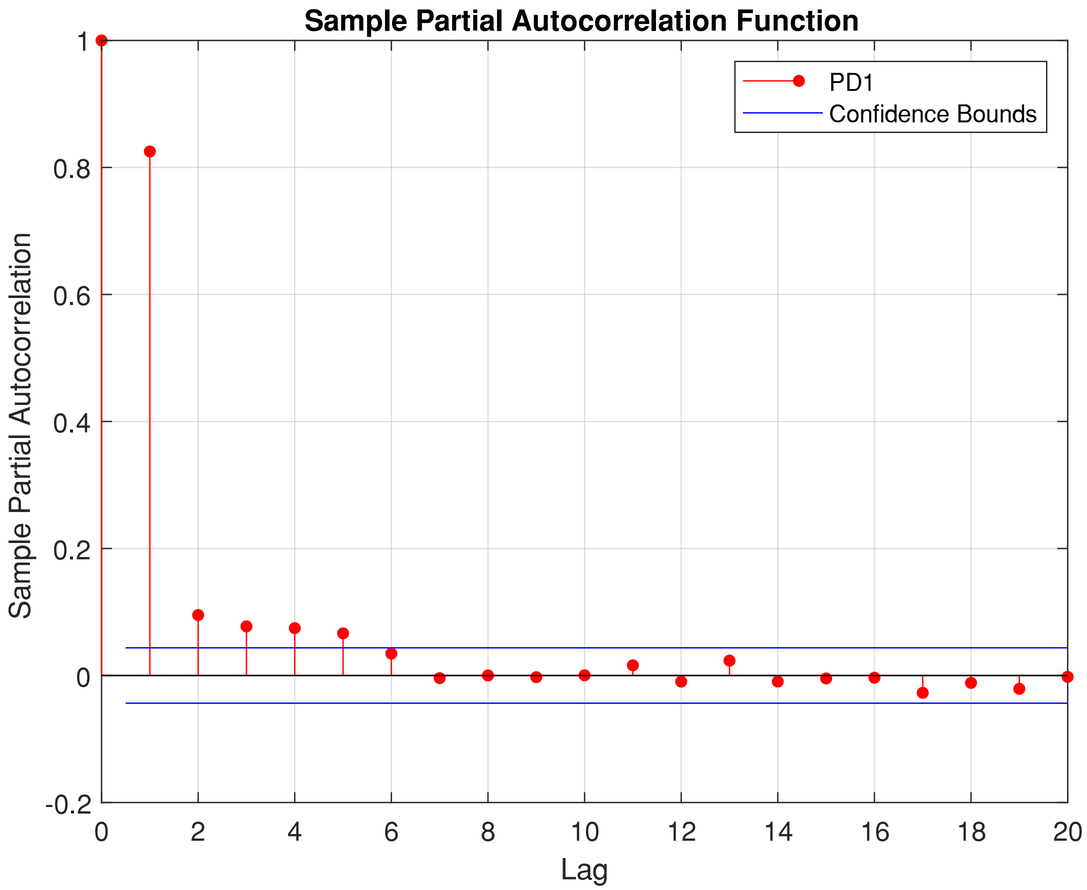

Figure S3.4. Sample partial autocorrelation function of PD1

3.7. Augmented Dickey-Fuller Test

Null Hypothesis: PD1 contains a unit root

$$y_t = c + \delta t + \phi y_{t-1} + \beta_1 \Delta y_{t-1} + \dots + \beta_p \Delta y_{t-p} + \varepsilon_t$$

$$H_0 : \phi = 1$$

$$H_a : \phi < 1$$

Table S3.5. Test Parameters

	Lags	Model	Test Statistic	Significance Level
1	0	AR	t1	0.025
2	1	AR	t1	0.025

Table S3.6. Test Results

	Null Rejected	P-Value	Test Statistic	Critical Value
1	false	0.089199	-1.6736	-2.2297
2	false	0.1573	-1.3762	-2.2297

4.ARIMA(6,0,0) Model (Gaussian Distribution) (AR_PD1)

Autoregressive model of time series PD1 with the following equation:

$$(1 - \phi_1 L - \ldots - \phi_6 L^6) y_t = c + \varepsilon_t$$

4.1. Model Estimation

Table S4.1. Estimation Results

Parameter	Value	Standard Error	t Statistic	P-Value
Constant	0.077606	0.010461	7.4189	1.181e-13
AR{1}	0.7266	0.0097309	74.6692	0
AR{2}	0.032565	0.0172	1.8933	0.058321
AR{3}	0.019539	0.020517	0.95233	0.34093
AR{4}	0.024956	0.039448	0.63263	0.52698
AR{5}	0.04137	0.046724	0.8854	0.37594
AR{6}	0.034521	0.035417	0.97471	0.3297
Variance	0.0022127	2.4091e-05	91.8441	0

Table S4.2. Goodness of Fit

AIC	-6882.5811
BIC	-6837.3835

4.2. Residual Quantile-Quantile plot

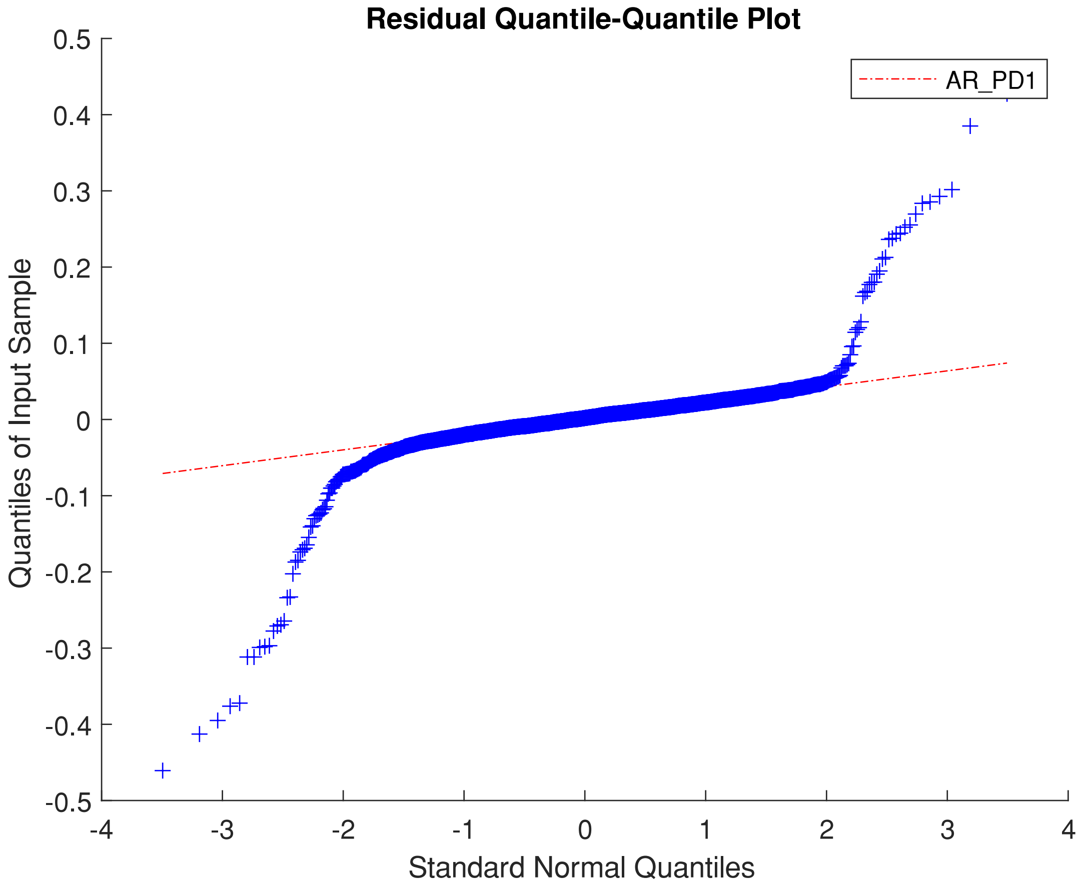

Figure S4.1. Quantile-quantile plot of the residuals of model AR_PD1.

4.3. Residual Sample Autocorrelation Function

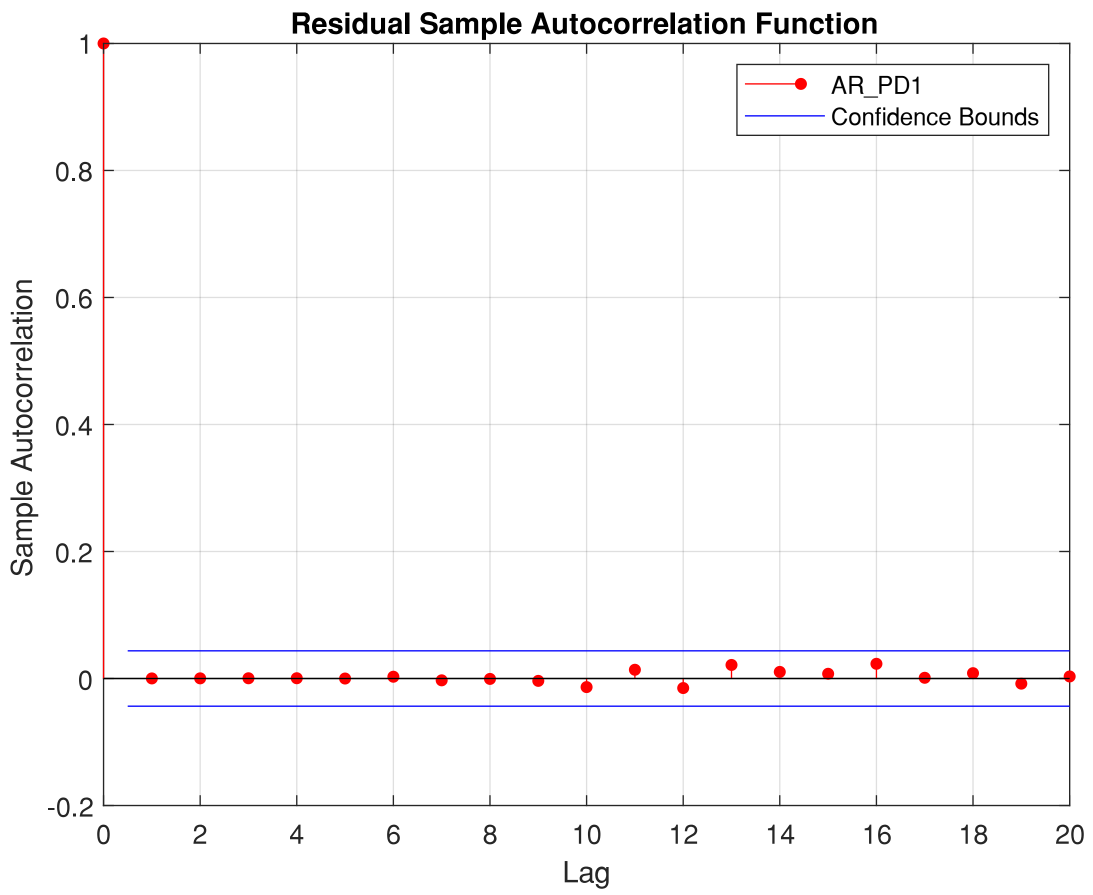

Figure S4.2. Sample autocorrelation function of the residuals of AR_PD1

4.4. Ljung-Box Q-Test

Null Hypothesis: The first m autocorrelations of the residuals of AR_PD1 are jointly 0

$$H_0 : \rho_1 = \rho_2 = \ldots = \rho_m = 0$$

$$H_a : \rho_j \neq 0, \ j \in 1, \ldots, m$$

Table S4.3. Test Parameters

	Lags	DOF	Significance Level
1	10	10	0.025

Table S4.4. Test Results

	Null Rejected	P-Value	Test Statistic	Critical Value
1	false	1	0.45188	20.4832

3. GCtest 'blockwise'

H0	Decision	Distribution	Statistic	PValue	CriticalValue
"Exclude lagged tDCS in HbT,PD equations"	"Reject H0"	"F(52,1997)"	1.6876	0.0017003	1.3496

H0	Decision	Distribution	Statistic	PValue	CriticalValue
"Exclude lagged tDCS in HbT equations"	"Reject H0"	"F(26,1997)"	1.9044	0.0039087	1.5012

H0	Decision	Distribution	Statistic	PValue	CriticalValue
"Exclude lagged tDCS in PD equations"	"Cannot reject H0"	"F(26,1997)"	1.4773	0.057114	1.5012

SUBJECT 3:

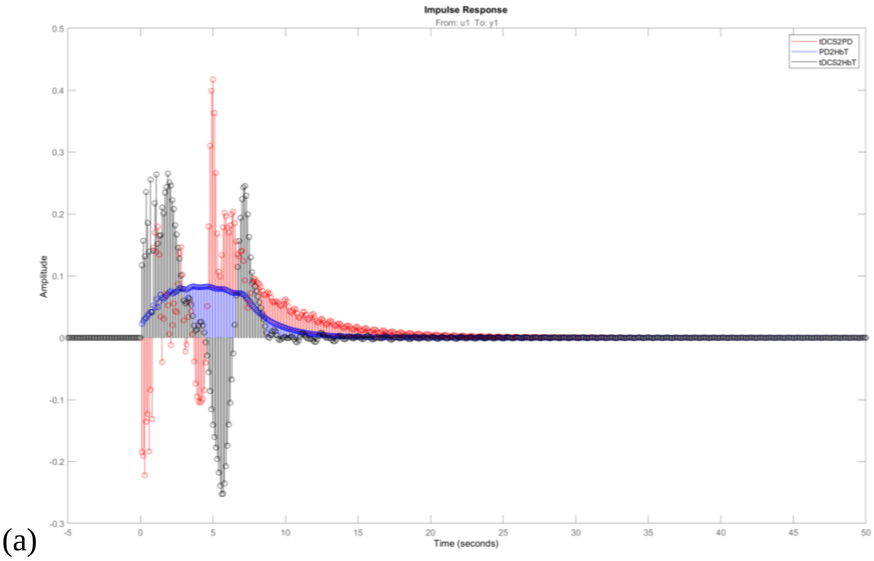

(a)

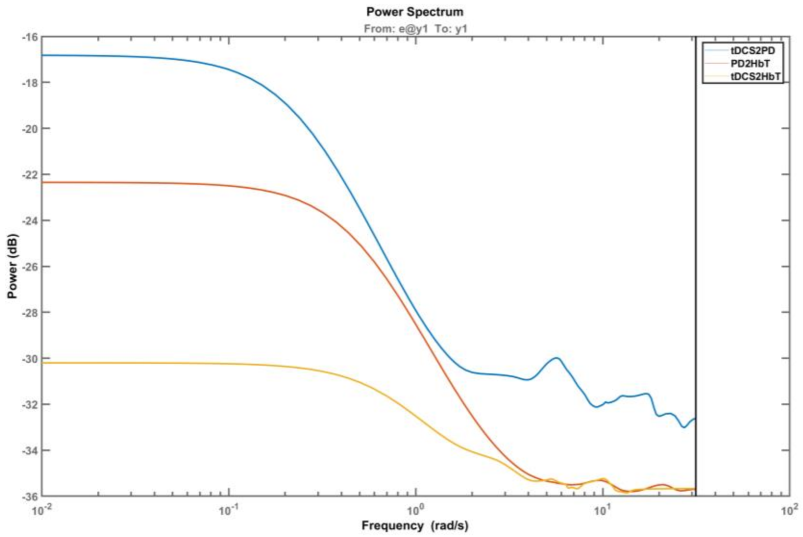

(b)

Figure S3: Subject 3 (a) impulse responses of these state-space models using regularized reduction of an ARX model. Partly, an anti-phase relationship between tDCS-evoked pupil diameter changes (tDCS2PD line in red) and tDCS-evoked blood volume changes (tDCS2HbT line in black) can be seen. Then, the pupil diameter changes as input and the blood volume changes as output (PD2HbT line in blue) has no initial dip at tDCS onset (near t=0). (b) Spectrum of the black box models with tDCS2PD line in blue, PD2HbT line in red, and tDCS2HbT line in yellow.

4.5. Sample Autocorrelation Function

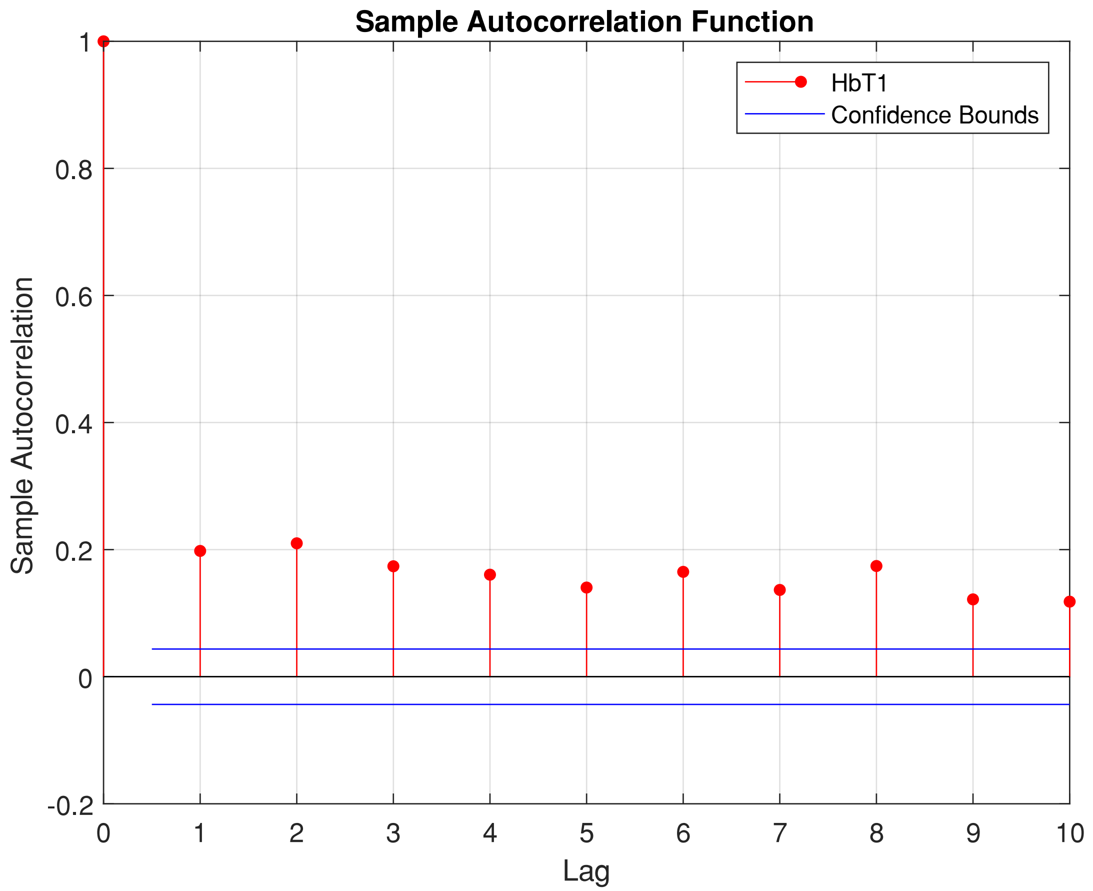

Figure S4.3. Sample autocorrelation function of HbT1

4.6. Sample Partial Autocorrelation Function

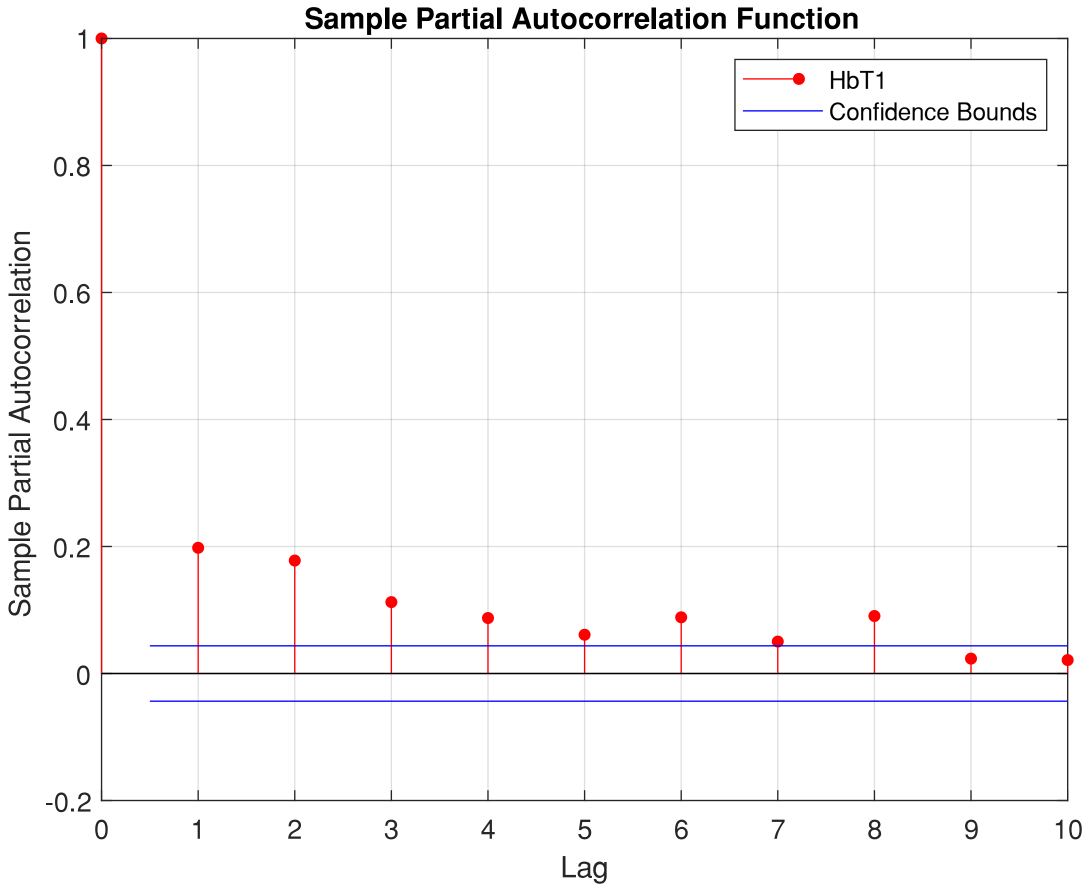

Figure S4.4. Sample partial autocorrelation function of HbT1

4.7. Augmented Dickey-Fuller Test

Null Hypothesis: HbT1 contains a unit root

$$y_t = c + \delta t + \phi y_{t-1} + \beta_1 \Delta y_{t-1} + \dots + \beta_p \Delta y_{t-p} + \varepsilon_t$$

$$H_0 : \phi = 1$$

$$H_a : \phi < 1$$

Table S4.5. Test Parameters

	Lags	Model	Test Statistic	Significance Level
1	0	AR	t1	0.025
2	1	AR	t1	0.025

Table S4.6. Test Results

	Null Rejected	P-Value	Test Statistic	Critical Value
1	true	0.0029907	-3.0472	-2.2297
2	false	0.076229	-1.7494	-2.2297

5.ARIMA(8,0,0) Model (Gaussian Distribution) (AR_HbT1)

Autoregressive model of time series HbT1 with the following equation:

$$(1 - \phi_1 L - \dots - \phi_8 L^8) y_t = c + \varepsilon_t$$

5.1. Model Estimation

Table S5.1. Estimation Results

Parameter	Value	Standard Error	t Statistic	P-Value
Constant	0.21416	0.021149	10.1261	4.2299e-24
AR{1}	0.11361	0.022018	5.1598	2.4723e-07
AR{2}	0.12308	0.021069	5.8417	5.1674e-09
AR{3}	0.075612	0.022939	3.2963	0.00097978
AR{4}	0.057032	0.021642	2.6352	0.0084095
AR{5}	0.03634	0.022088	1.6453	0.099918
AR{6}	0.070459	0.021567	3.267	0.001087
AR{7}	0.039795	0.021849	1.8214	0.06855
AR{8}	0.09124	0.021533	4.2372	2.2633e-05
Variance	0.0028686	9.1648e-05	31.3008	4.5548e-215

Table S5.2. Goodness of Fit

AIC	-6331.7778
BIC	-6275.2904

5.2. Residual Quantile-Quantile plot

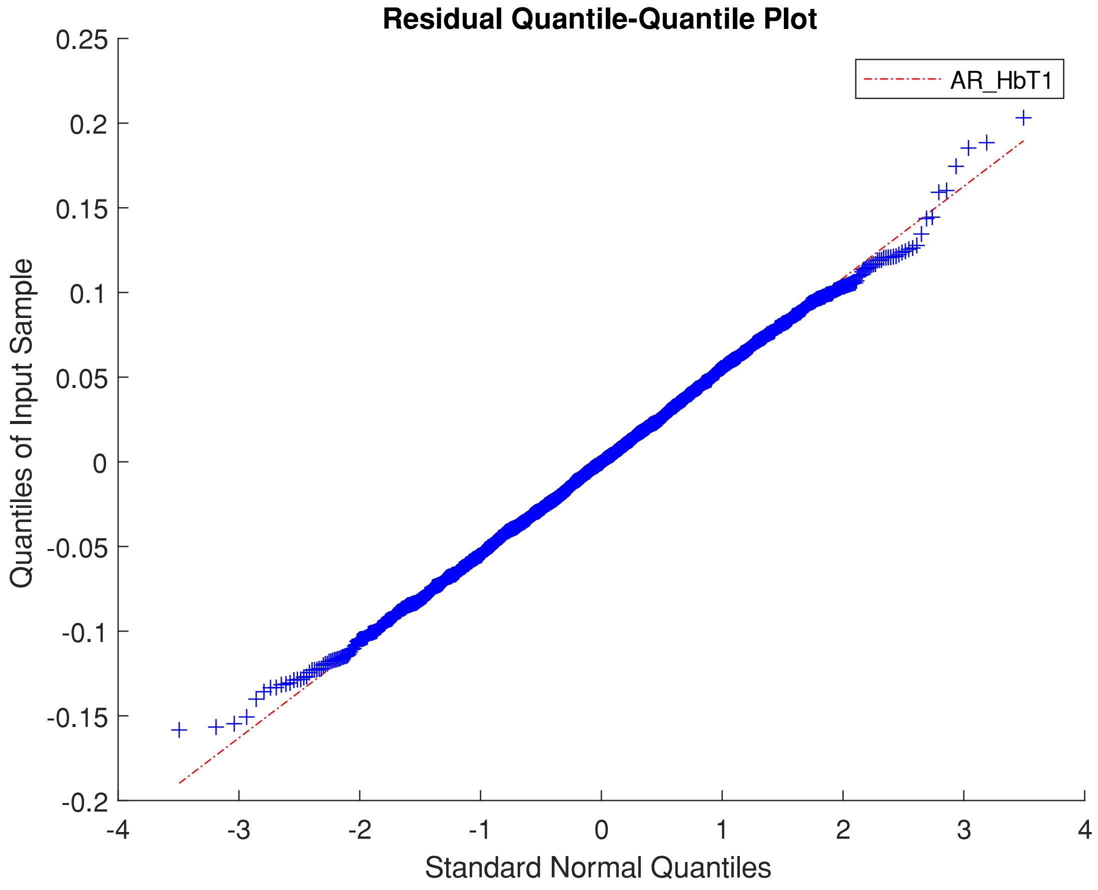

Figure S5.1. Quantile-quantile plot of the residuals of model AR_HbT1.

5.3. Residual Sample Autocorrelation Function

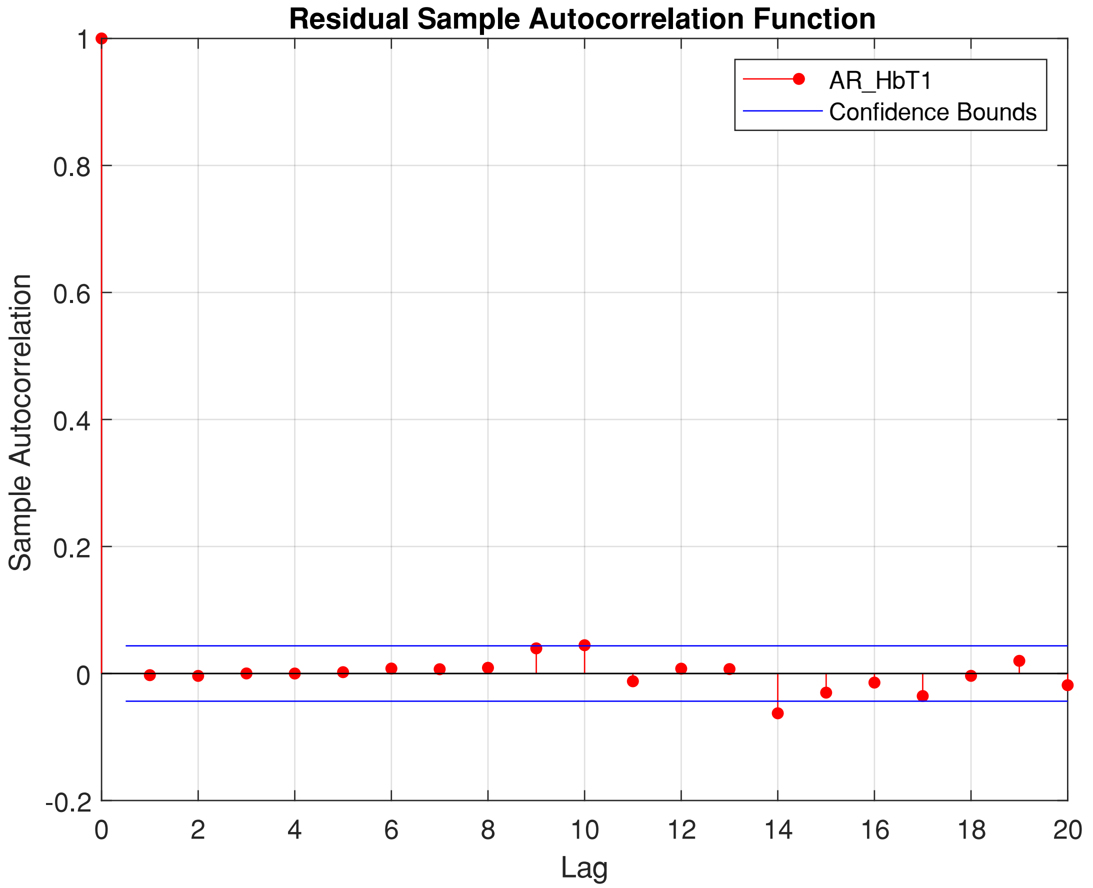

Figure S5.2. Sample autocorrelation function of the residuals of AR_HbT1

5.4. Ljung-Box Q-Test

Null Hypothesis: The first m autocorrelations of the residuals of AR_HbT1 are jointly 0

$$H_0 : \rho_1 = \rho_2 = \ldots = \rho_m = 0$$

$$H_a : \rho_j \neq 0, \ j \in 1, \ldots, m$$

Table S5.3. Test Parameters

	Lags	DOF	Significance Level
1	10	10	0.025

Table S5.4. Test Results

	Null Rejected	P-Value	Test Statistic	Critical Value
1	false	0.63155	7.9723	20.4832

5.5. Sample Autocorrelation Function

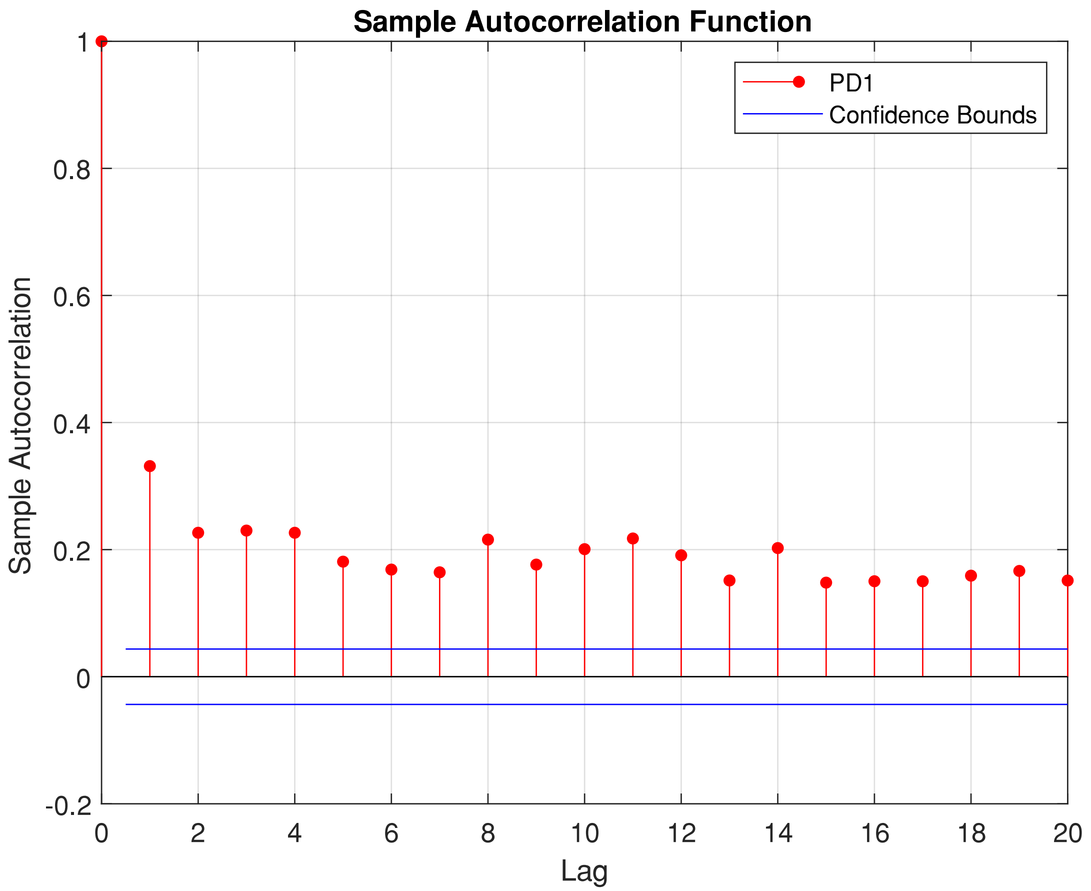

Figure S5.3. Sample autocorrelation function of PD1

5.6. Sample Partial Autocorrelation Function

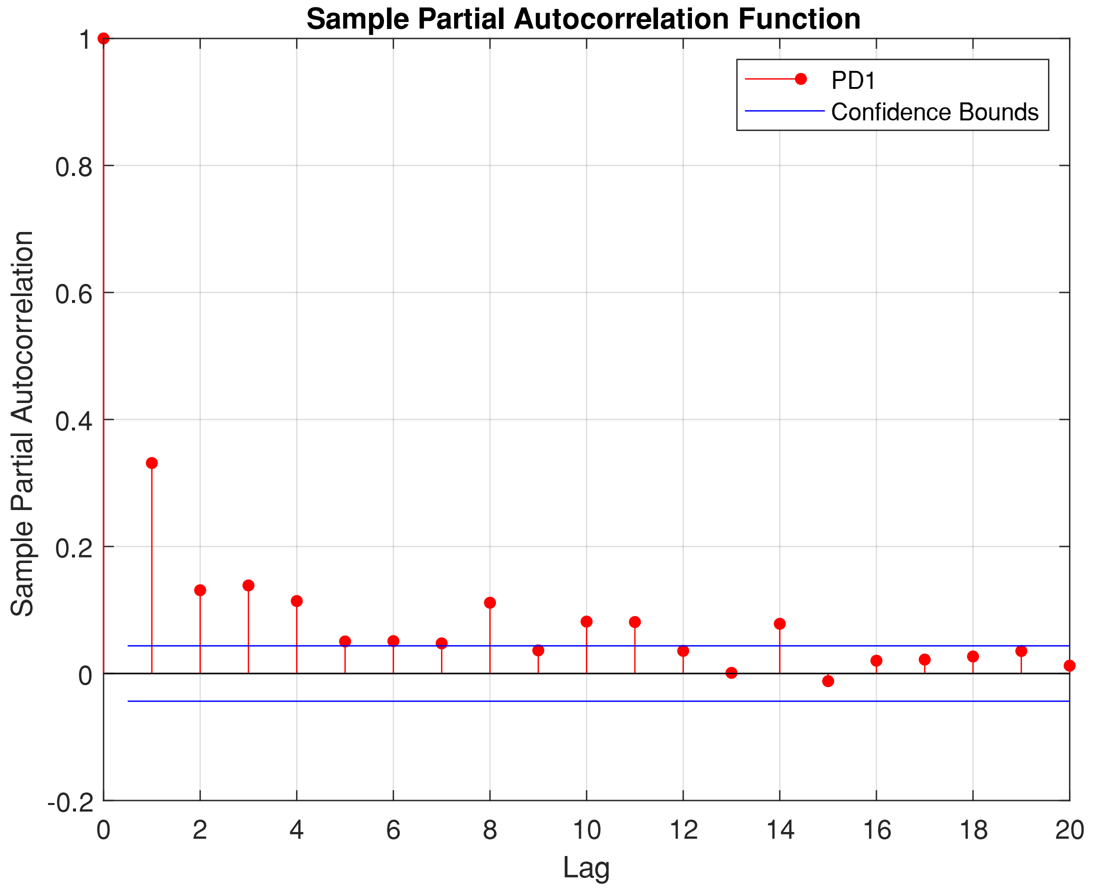

Figure S5.4. Sample partial autocorrelation function of PD1

5.7. Augmented Dickey-Fuller Test

Null Hypothesis: PD1 contains a unit root

$$y_t = c + \delta t + \phi y_{t-1} + \beta_1 \Delta y_{t-1} + \ldots + \beta_p \Delta y_{t-p} + \varepsilon_t$$

$$H_0 : \phi = 1$$

$$H_a : \phi < 1$$

Table S5.5. Test Parameters

	Lags	Model	Test Statistic	Significance Level
1	0	AR	t1	0.025
2	1	AR	t1	0.025

Table S5.6. Test Results

	Null Rejected	P-Value	Test Statistic	Critical Value
1	true	0.0039226	-2.9338	-2.2297
2	false	0.057636	-1.8788	-2.2297

6.ARIMA(7,0,0) Model (Gaussian Distribution) (AR_PD1)

Autoregressive model of time series PD1 with the following equation:

$$(1 - \phi_1 L - \dots - \phi_7 L^7) y_t = c + \varepsilon_t$$

6.1. Model Estimation

Table S6.1. Estimation Results

Parameter	Value	Standard Error	t Statistic	P-Value
Constant	0.32607	0.031601	10.3185	5.8131e-25
AR{1}	0.24325	0.021655	11.2329	2.8098e-29
AR{2}	0.068397	0.027074	2.5263	0.011528
AR{3}	0.093924	0.026064	3.6036	0.00031389
AR{4}	0.092795	0.020917	4.4363	9.1506e-06
AR{5}	0.034481	0.031067	1.1099	0.26705
AR{6}	0.039357	0.031843	1.236	0.21647
AR{7}	0.047477	0.03203	1.4823	0.13827
Variance	0.0075954	0.00013306	57.0816	0

Table S6.2. Goodness of Fit

AIC	-4283.1631
BIC	-4232.3202

6.2. Residual Quantile-Quantile plot

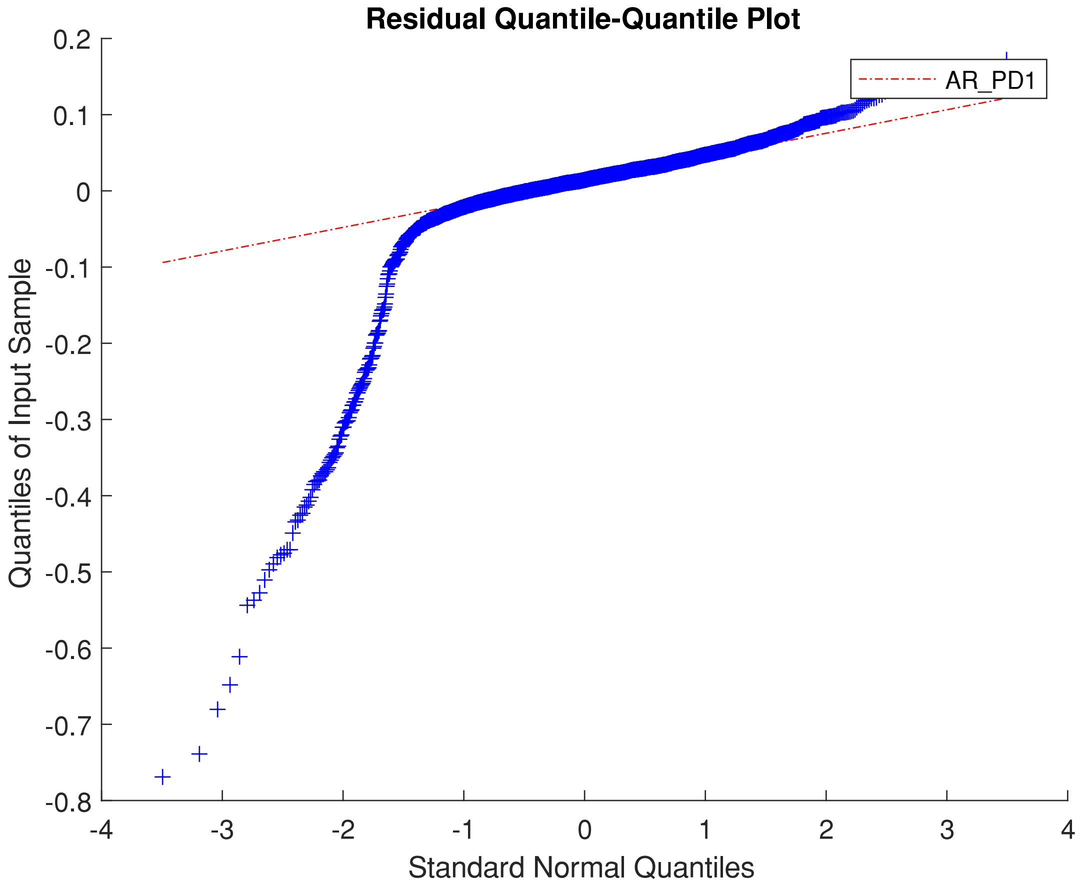

Figure S6.1. Quantile-quantile plot of the residuals of model AR_PD1.

6.3. Residual Sample Autocorrelation Function

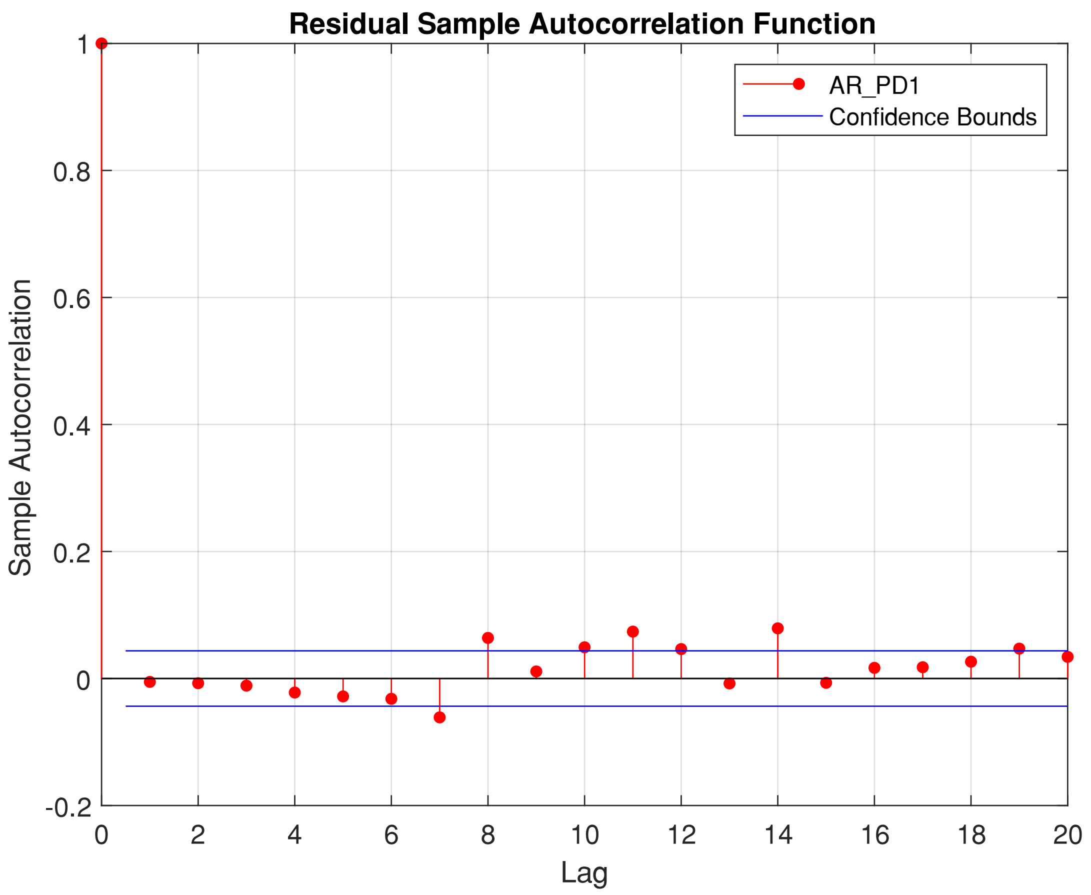

Figure S6.2. Sample autocorrelation function of the residuals of AR_PD1

6.4. Ljung-Box Q-Test

Null Hypothesis: The first m autocorrelations of the residuals of AR_PD1 are jointly 0

$$H_0 : \rho_1 = \rho_2 = \ldots = \rho_m = 0$$

$$H_a : \rho_j \neq 0, j \in 1, \ldots, m$$

Table S6.3. Test Parameters

	Lags	DOF	Significance Level
1	10	10	0.025

Table S6.4. Test Results

	Null Rejected	P-Value	Test Statistic	Critical Value
1	true	0.002372	27.2561	20.4832

3. GCtest 'blockwise'

H0	Decision	Distribution	Statistic	PValue	CriticalValue
"Exclude lagged tDCS in HbT,PD equations"	"Reject H0"	"F(34,2024)"	2.2734	4.1574e-05	1.4353

H0	Decision	Distribution	Statistic	PValue	CriticalValue
"Exclude lagged tDCS in HbT equations"	"Reject H0"	"F(17,2024)"	2.3424	0.0014826	1.6278

H0	Decision	Distribution	Statistic	PValue	CriticalValue
"Exclude lagged tDCS in PD equations"	"Reject H0"	"F(17,2024)"	2.1752	0.0036032	1.6278

SUBJECT 4:

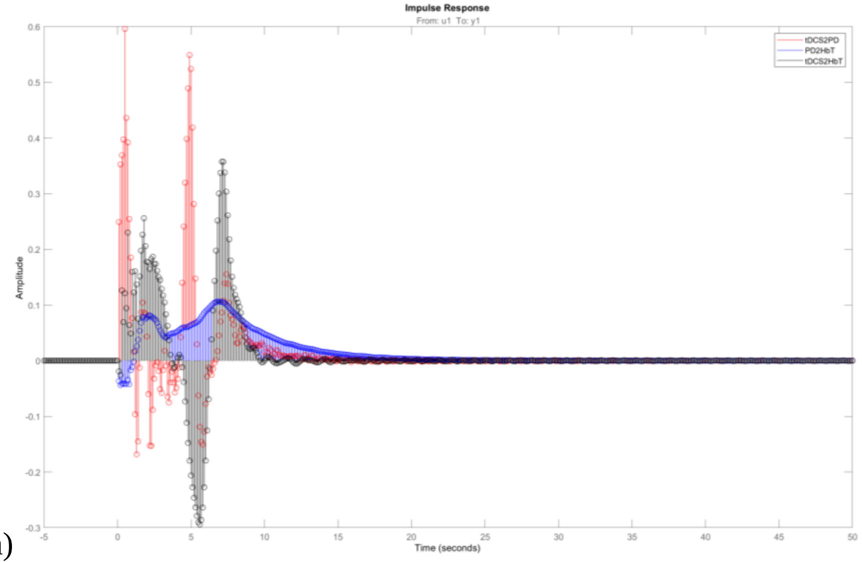

(a)

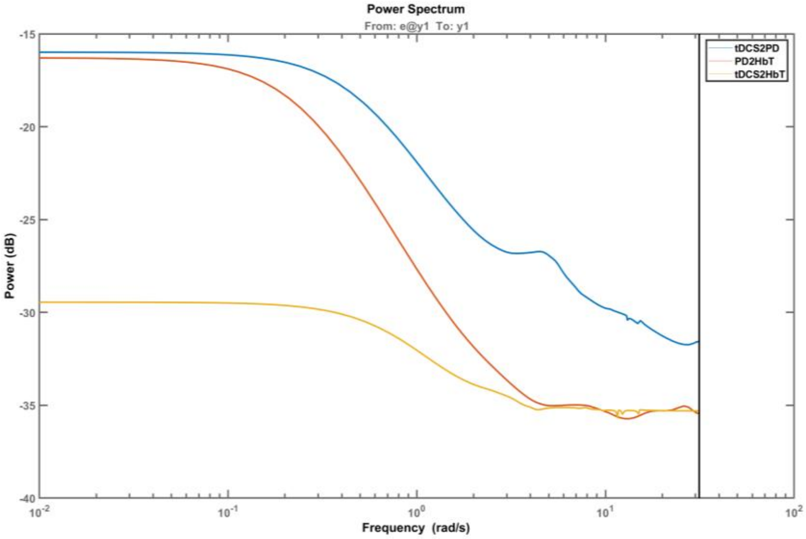

(b)

Figure S4: Subject 4 (a) impulse responses of these state-space models using regularized reduction of an ARX model. Partly, an anti-phase relationship between tDCS-evoked pupil diameter changes (tDCS2PD line in red) and tDCS-evoked blood volume changes (tDCS2HbT line in black) can be seen. Then, the pupil diameter changes as input and the blood volume changes as output (PD2HbT line in blue) has an initial dip at tDCS onset (near t=0). (b) Spectrum of the black box models with tDCS2PD line in blue, PD2HbT line in red, and tDCS2HbT line in yellow.

6.5. Sample Autocorrelation Function

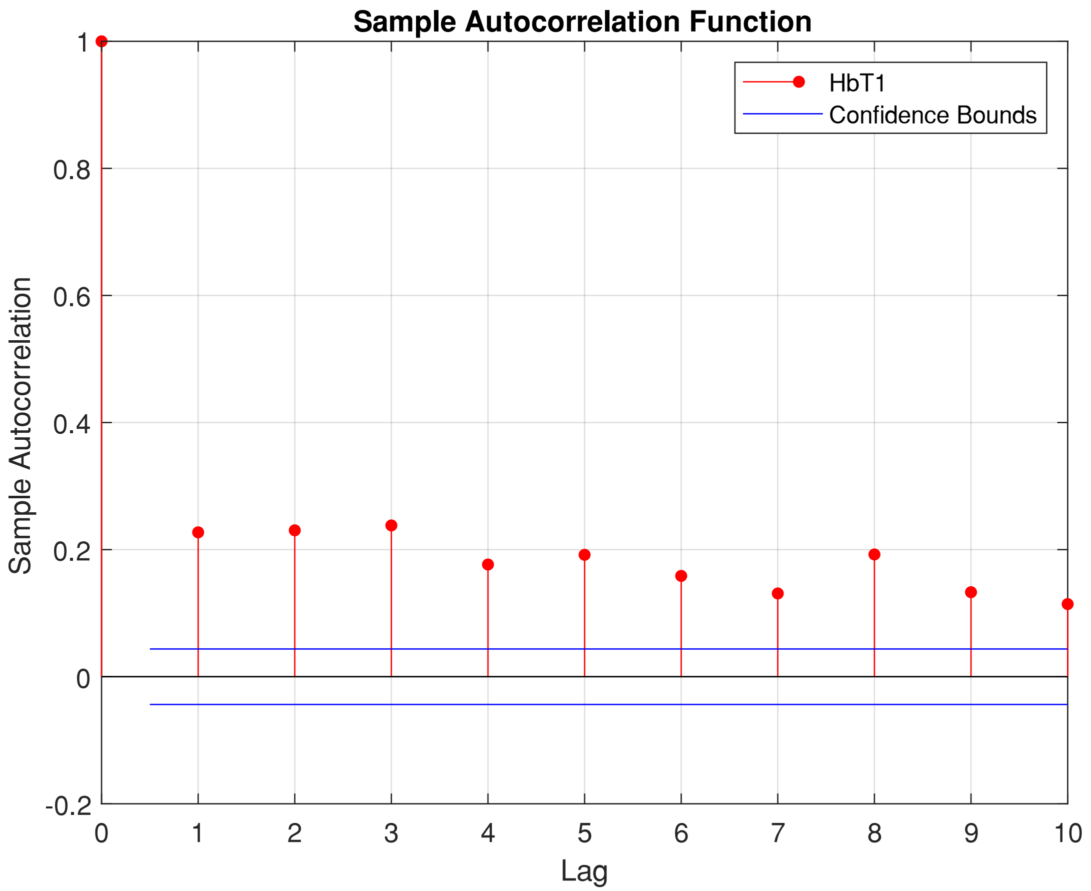

Figure S6.3. Sample autocorrelation function of HbT1

6.6. Sample Partial Autocorrelation Function

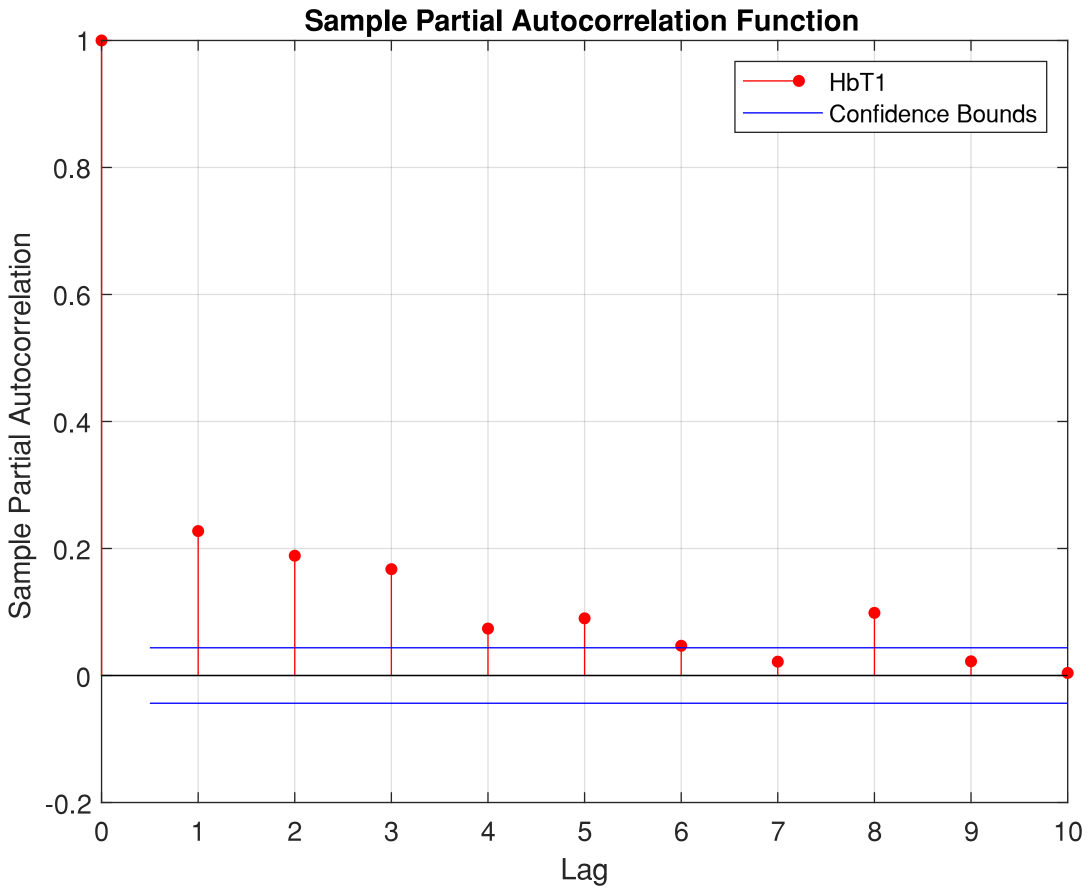

Figure S6.4. Sample partial autocorrelation function of HbT1

6.7. Augmented Dickey-Fuller Test

Null Hypothesis: HbT1 contains a unit root

$$y_t = c + \delta t + \phi y_{t-1} + \beta_1 \Delta y_{t-1} + \ldots + \beta_p \Delta y_{t-p} + \varepsilon_t$$

$$H_0 : \phi = 1$$

$$H_a : \phi < 1$$

Table S6.5. Test Parameters

	Lags	Model	Test Statistic	Significance Level
1	0	AR	t1	0.025
2	1	AR	t1	0.025

Table S6.6. Test Results

	Null Rejected	P-Value	Test Statistic	Critical Value
1	true	0.0030432	-3.0408	-2.2297
2	false	0.075324	-1.7549	-2.2297

7.ARIMA(8,0,0) Model (Gaussian Distribution) (AR_HbT1)

Autoregressive model of time series HbT1 with the following equation:

$$(1 - \phi_1 L - \ldots - \phi_8 L^8) y_t = c + \varepsilon_t$$

7.1. Model Estimation

Table S7.1. Estimation Results

Parameter	Value	Standard Error	t Statistic	P-Value
Constant	0.20393	0.020071	10.1601	2.9875e-24

AR{1}	0.12647	0.021665	5.8377	5.2922e-09
AR{2}	0.12318	0.023081	5.3368	9.4627e-08
AR{3}	0.12703	0.021535	5.8988	3.6623e-09
AR{4}	0.0473	0.022711	2.0827	0.037278
AR{5}	0.06769	0.022794	2.9696	0.0029821
AR{6}	0.031609	0.021644	1.4604	0.14417
AR{7}	0.0084907	0.021697	0.39134	0.69555
AR{8}	0.099317	0.021107	4.7053	2.5346e-06
Variance	0.0031003	9.5729e-05	32.386	4.3177e-230

Table S7.2. Goodness of Fit

AIC	-6144.727
BIC	-6088.2778

7.2. Residual Quantile-Quantile plot

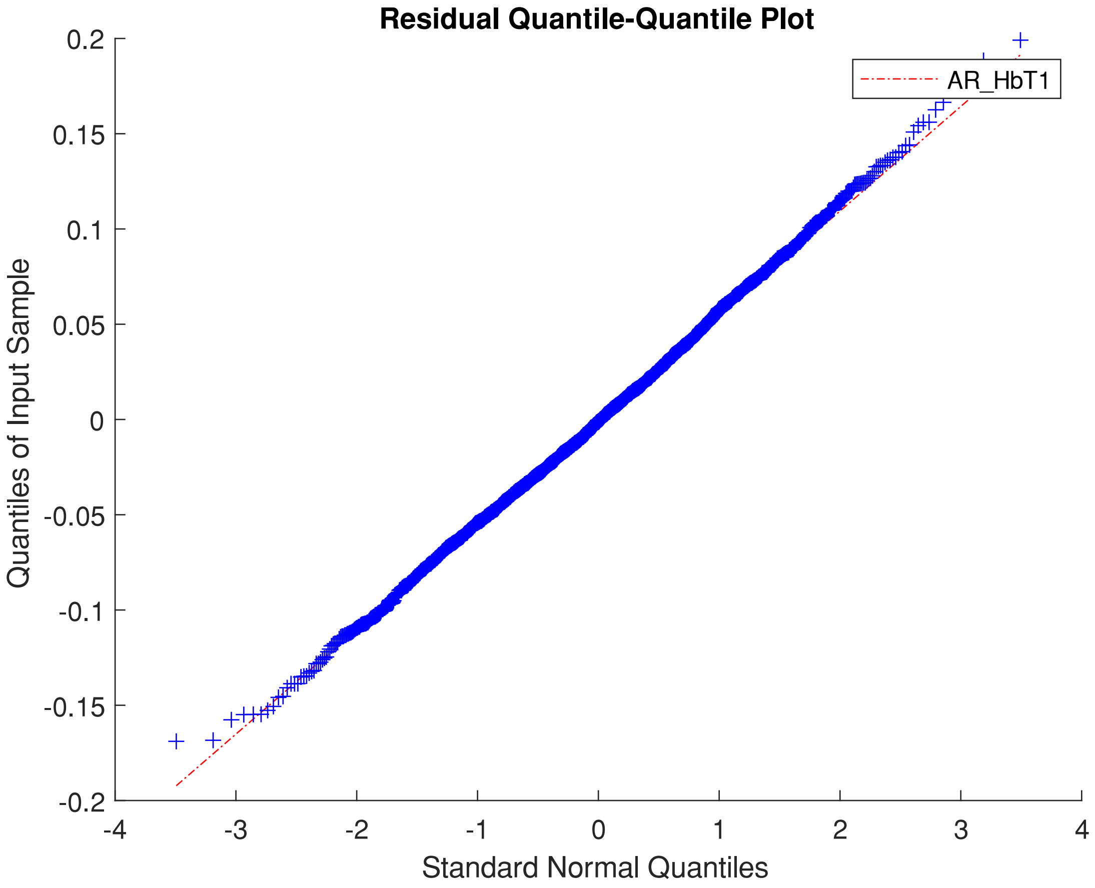

Figure S7.1. Quantile-quantile plot of the residuals of model AR_HbT1.

7.3. Residual Sample Autocorrelation Function

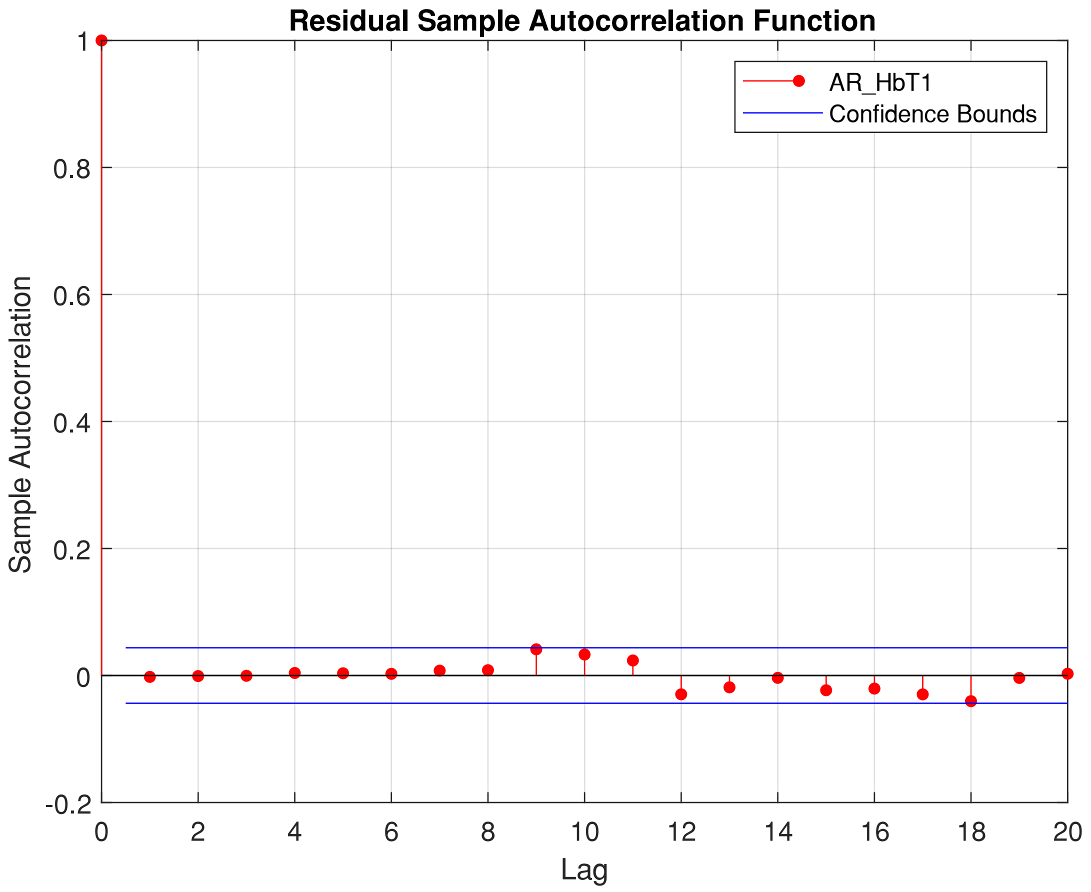

Figure S7.2. Sample autocorrelation function of the residuals of AR_HbT1

7.4. Ljung-Box Q-Test

Null Hypothesis: The first m autocorrelations of the residuals of AR_HbT1 are jointly 0

$$H_0 : \rho_1 = \rho_2 = \ldots = \rho_m = 0$$

$$H_a : \rho_j \neq 0, j \in 1, \ldots, m$$

Table S7.3. Test Parameters

	Lags	DOF	Significance Level
1	10	10	0.025

Table S7.4. Test Results

	Null Rejected	P-Value	Test Statistic	Critical Value
1	false	0.794	6.2481	20.4832

7.5. Sample Autocorrelation Function

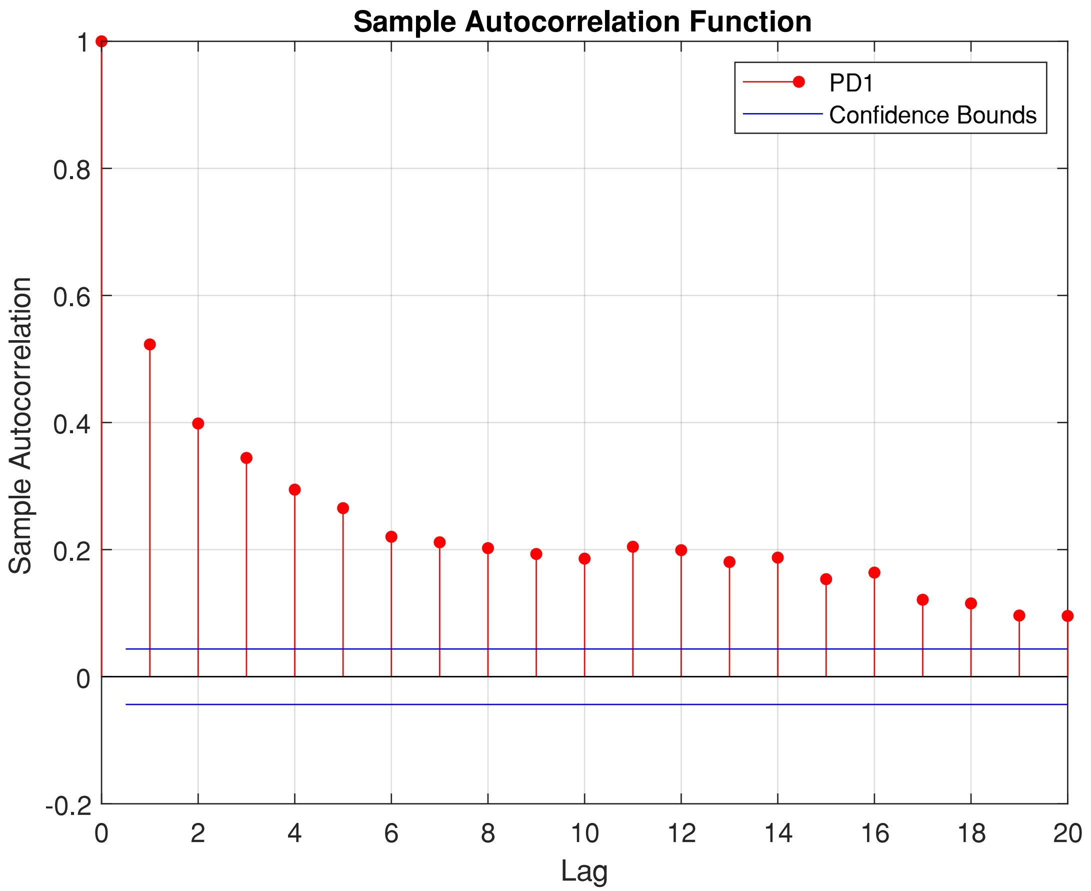

Figure S7.3. Sample autocorrelation function of PD1

7.6. Sample Partial Autocorrelation Function

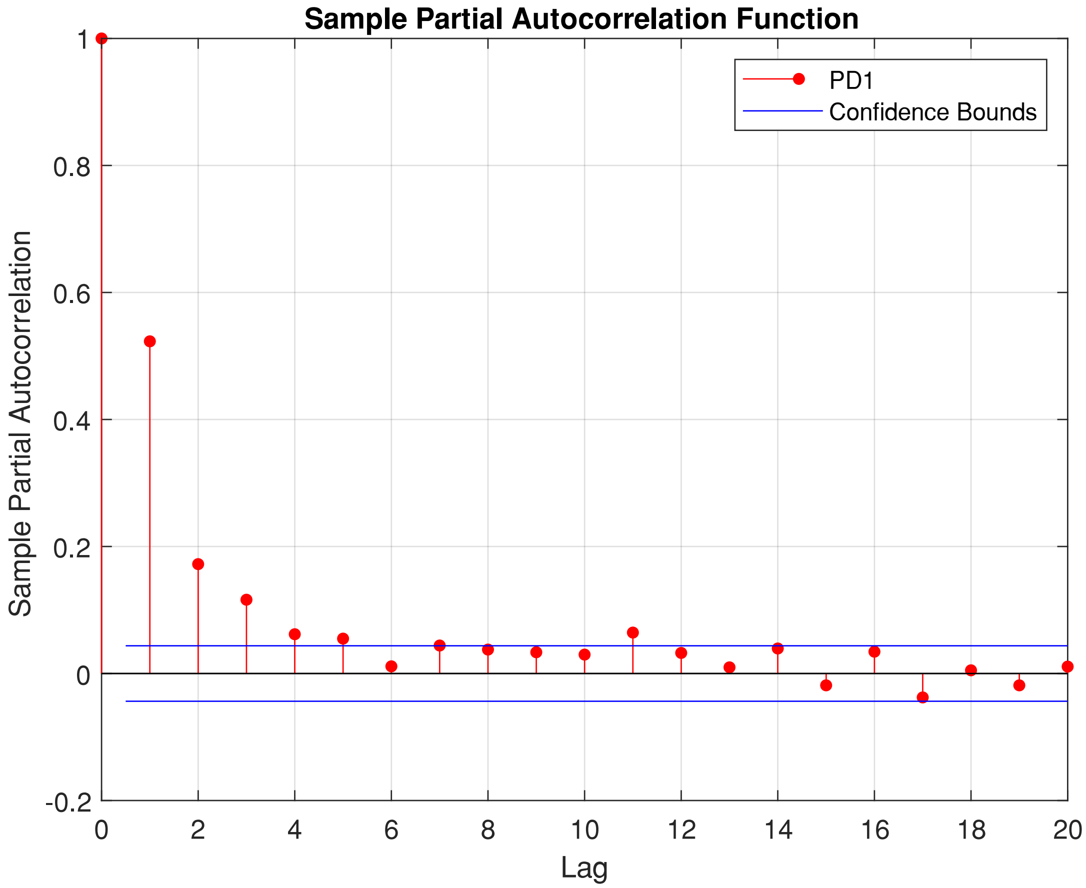

Figure S7.4. Sample partial autocorrelation function of PD1

7.7. Augmented Dickey-Fuller Test

Null Hypothesis: PD1 contains a unit root

$$y_t = c + \delta t + \phi y_{t-1} + \beta_1 \Delta y_{t-1} + \ldots + \beta_p \Delta y_{t-p} + \varepsilon_t$$

$$H_0 : \phi = 1$$

$$H_a : \phi < 1$$

Table S7.5. Test Parameters

	Lags	Model	Test Statistic	Significance Level
1	0	AR	t1	0.025
2	1	AR	t1	0.025
3	2	AR	t1	0.025

Table S7.6. Test Results

	Null Rejected	P-Value	Test Statistic	Critical Value
1	true	0.001	-3.9264	-2.2297
2	true	0.0076111	-2.6806	-2.2297
3	false	0.034412	-2.1012	-2.2297

8.ARIMA(7,0,0) Model (Gaussian Distribution) (AR_PD1)

Autoregressive model of time series PD1 with the following equation:

$$(1 - \phi_1 L - \dots - \phi_7 L^7) y_t = c + \varepsilon_t$$

8.1. Model Estimation

Table S8.1. Estimation Results

Parameter	Value	Standard Error	t Statistic	P-Value
Constant	0.21007	0.018614	11.2853	1.5517e-29
AR{1}	0.40136	0.01345	29.8408	1.1563e-195
AR{2}	0.1068	0.020085	5.3173	1.0533e-07
AR{3}	0.081733	0.020672	3.9538	7.6917e-05
AR{4}	0.03467	0.023506	1.475	0.14022
AR{5}	0.045591	0.020905	2.1809	0.029191
AR{6}	-0.0064368	0.035389	-0.18189	0.85567
AR{7}	0.044098	0.026355	1.6733	0.094277
Variance	0.011109	0.00020157	55.111	0

Table S8.2. Goodness of Fit

AIC	-3469.175
BIC	-3418.3664

8.2. Residual Quantile-Quantile plot

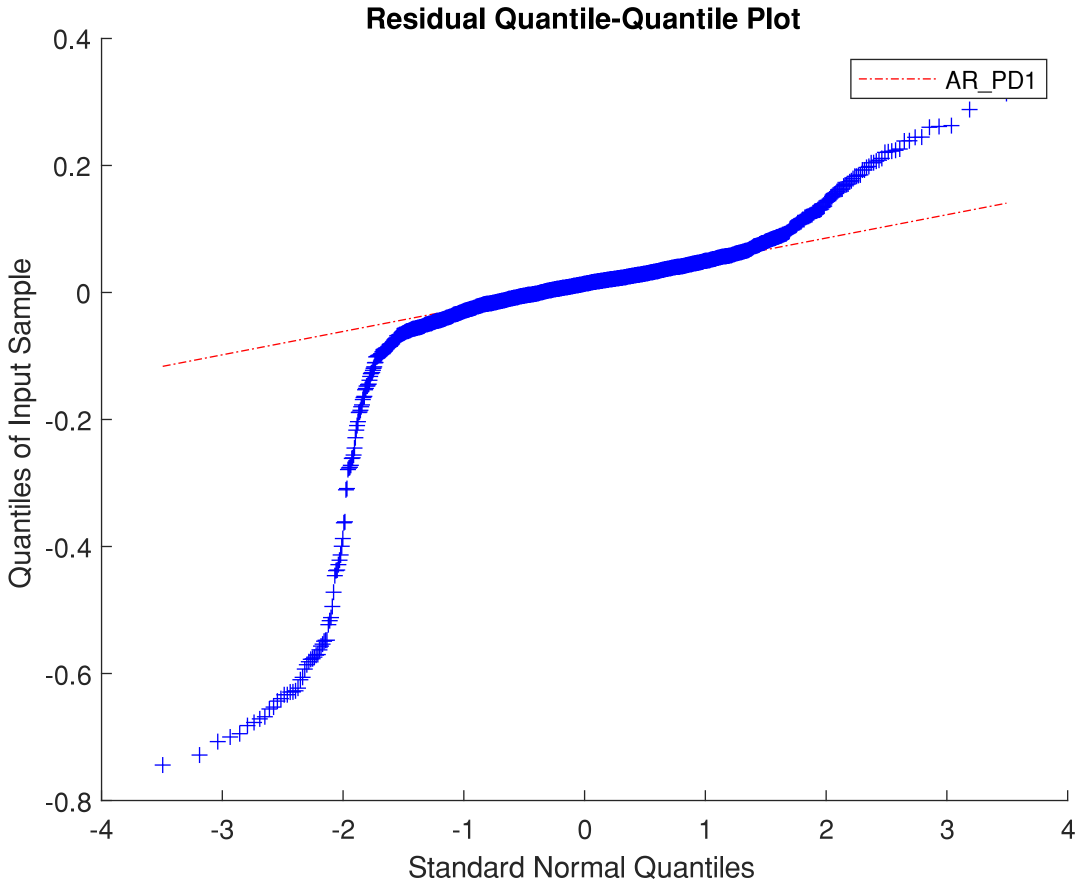

Figure S8.1. Quantile-quantile plot of the residuals of model AR_PD1.

8.3. Residual Sample Autocorrelation Function

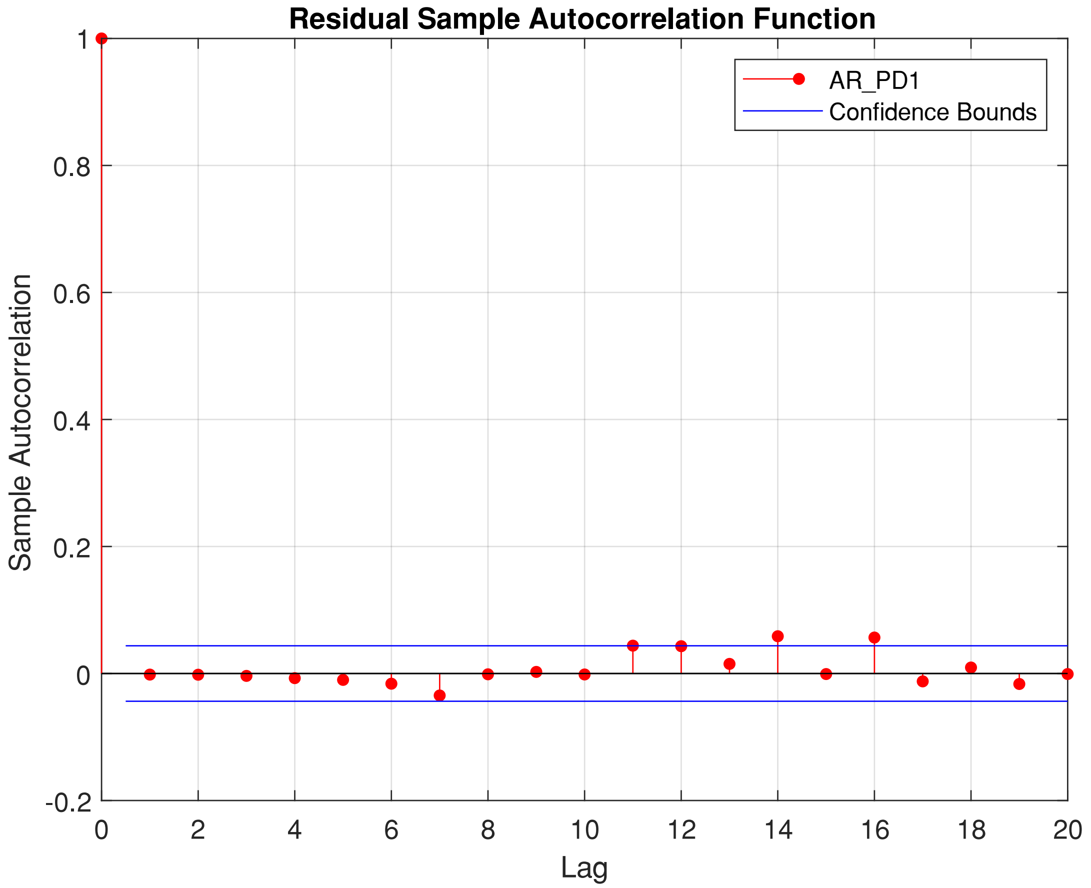

Figure S8.2. Sample autocorrelation function of the residuals of AR_PD1

8.4. Ljung-Box Q-Test

Null Hypothesis: The first m autocorrelations of the residuals of AR_PD1 are jointly 0

$$H_0 : \rho_1 = \rho_2 = \ldots = \rho_m = 0$$

$$H_a : \rho_j \neq 0, j \in 1, \ldots, m$$

Table S8.3. Test Parameters

	Lags	DOF	Significance Level
1	10	10	0.025

Table S8.4. Test Results

	Null Rejected	P-Value	Test Statistic	Critical Value
1	false	0.96867	3.4531	20.4832

3. GCtest 'blockwise'

H0	Decision	Distribution	Statistic	PValue	CriticalValue
"Exclude lagged tDCS in HbT,PD equations"	"Reject H0"	"F(36,2013)"	2.0446	0.00026555	1.4226

H0	Decision	Distribution	Statistic	PValue	CriticalValue
"Exclude lagged tDCS in HbT equations"	"Reject H0"	"F(18,2013)"	1.9065	0.012024	1.609

H0	Decision	Distribution	Statistic	PValue	CriticalValue
"Exclude lagged tDCS in PD equations"	"Reject H0"	"F(18,2013)"	2.1702	0.0029907	1.609

SUBJECT 5:

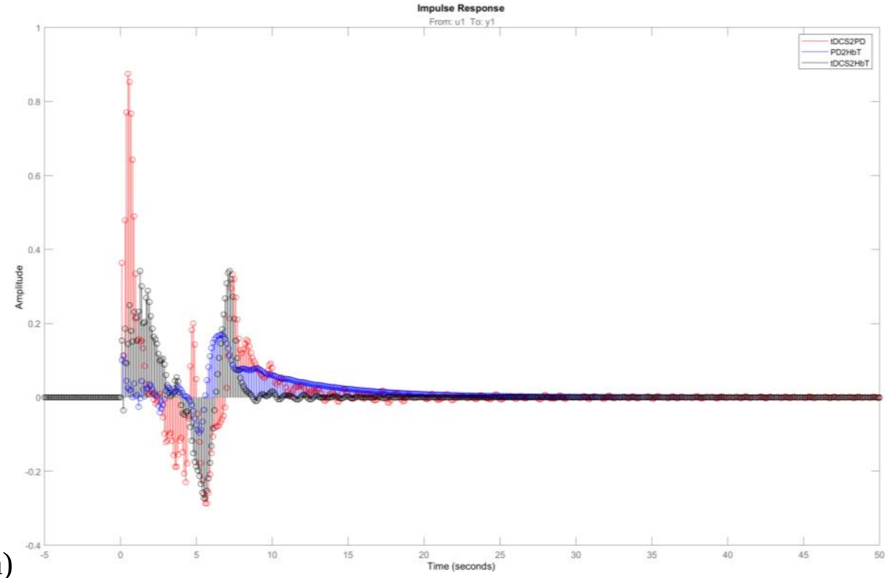

(a)

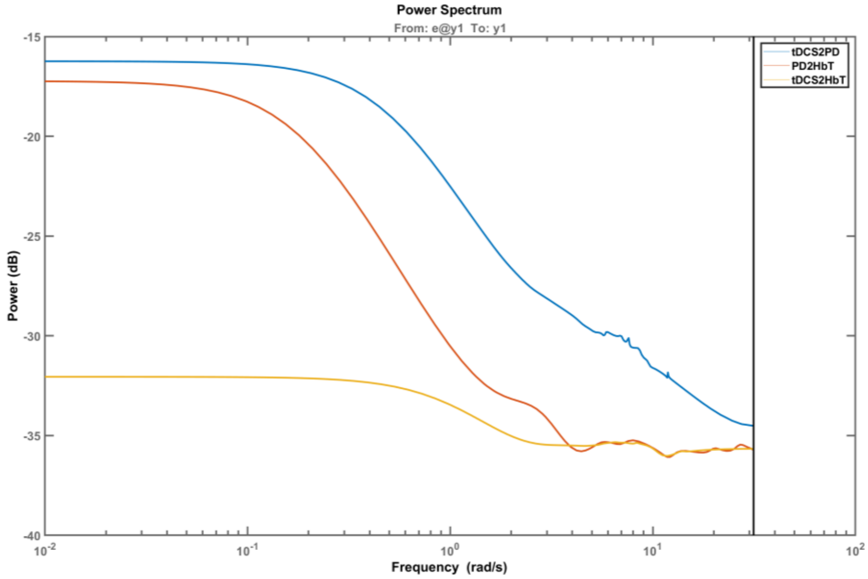

(b)

Figure S3: Subject 3 (a) impulse responses of these state-space models using regularized reduction of an ARX model. Partly, an anti-phase relationship between tDCS-evoked pupil diameter changes (tDCS2PD line in red) and tDCS-evoked blood volume changes (tDCS2HbT line in black) can be seen. Then, the pupil diameter changes as input and the blood volume changes as output (PD2HbT line in blue) has no initial dip at tDCS onset (near t=0). (b) Spectrum of the black box models with tDCS2PD line in blue, PD2HbT line in red, and tDCS2HbT line in yellow.

8.5. Sample Autocorrelation Function

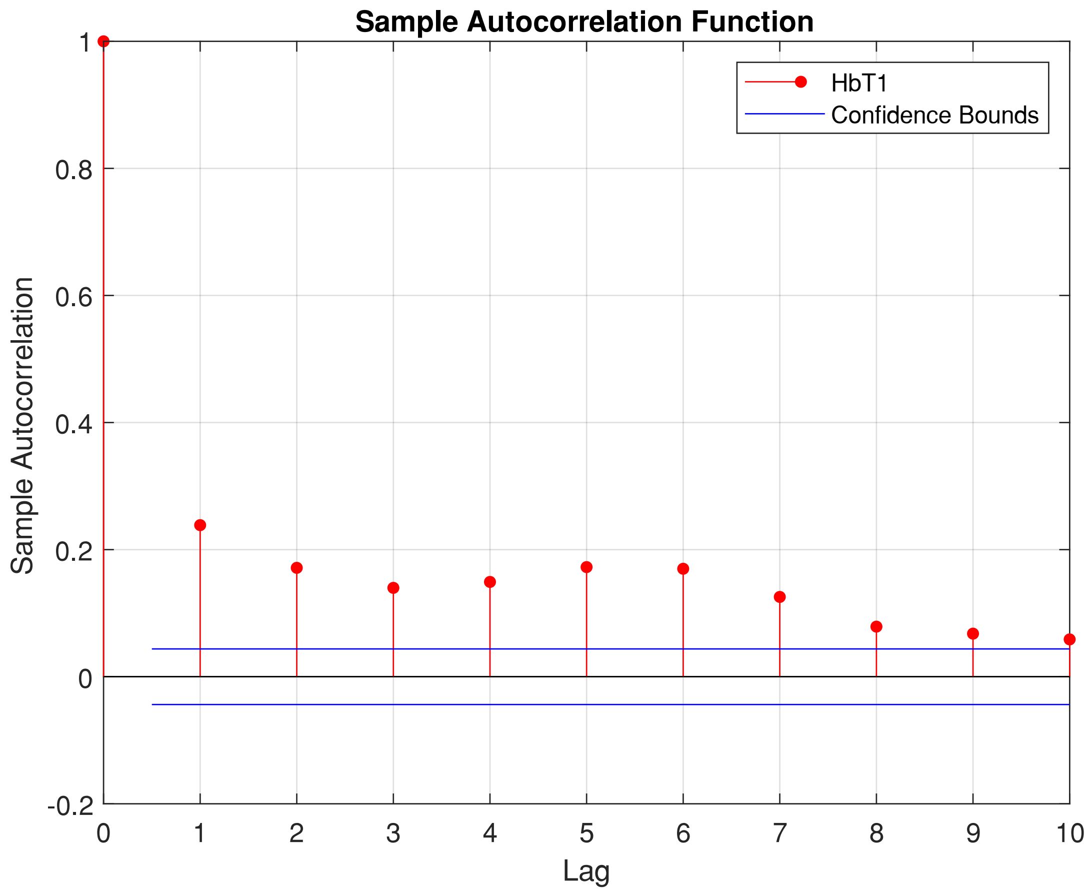

Figure 8.3. Sample autocorrelation function of HbT1

8.6. Sample Partial Autocorrelation Function

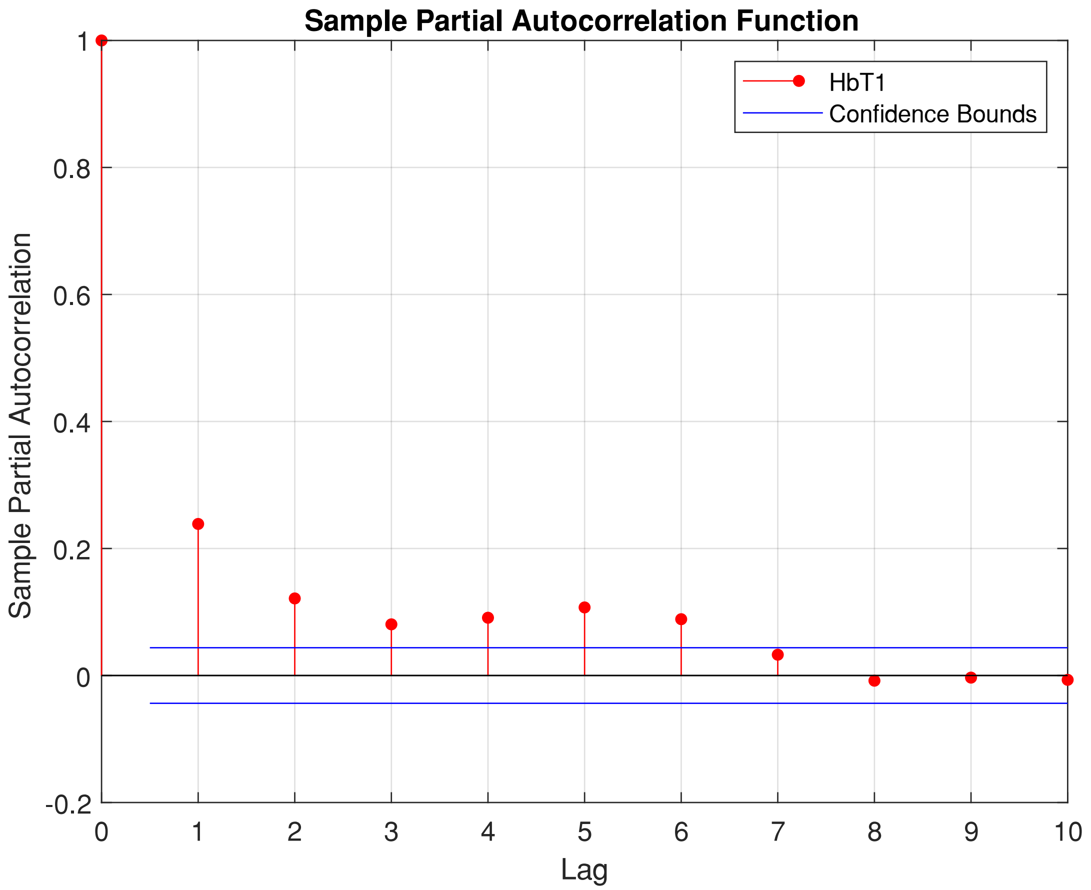

Figure 8.4. Sample partial autocorrelation function of HbT1

8.7. Augmented Dickey-Fuller Test

Null Hypothesis: HbT1 contains a unit root

$$y_t = c + \delta t + \phi y_{t-1} + \beta_1 \Delta y_{t-1} + \ldots + \beta_p \Delta y_{t-p} + \varepsilon_t$$

$$H_0 : \phi = 1$$

$$H_a : \phi < 1$$

Table 8.5. Test Parameters

	Lags	Model	Test Statistic	Significance Level
1	0	AR	t1	0.025
2	1	AR	t1	0.025

Table 8.6. Test Results

	Null Rejected	P-Value	Test Statistic	Critical Value
1	true	0.0045342	-2.8593	-2.2297
2	false	0.074558	-1.7598	-2.2297

9.ARIMA(8,0,0) Model (Gaussian Distribution) (AR_HbT1)

Autoregressive model of time series HbT1 with the following equation:

$$(1 - \phi_1 L - \ldots - \phi_8 L^8) y_t = c + \varepsilon_t$$

9.1. Model Estimation

Table 9.1. Estimation Results

Parameter	Value	Standard Error	t Statistic	P-Value
Constant	0.24299	0.021291	11.413	3.5998e-30
AR{1}	0.17045	0.021997	7.7488	9.277e-15
AR{2}	0.079595	0.022547	3.5301	0.00041534
AR{3}	0.046239	0.022355	2.0684	0.038606
AR{4}	0.061613	0.022318	2.7607	0.0057672
AR{5}	0.088834	0.022391	3.9673	7.2685e-05
AR{6}	0.083907	0.022364	3.7518	0.00017556
AR{7}	0.03427	0.021779	1.5735	0.1156
AR{8}	-0.0081881	0.021772	-0.37608	0.70686
Variance	0.0027967	8.8924e-05	31.4502	4.1766e-217

Table 9.2. Goodness of Fit

AIC	-6333.5761
BIC	-6277.17

9.2. Residual Quantile-Quantile plot

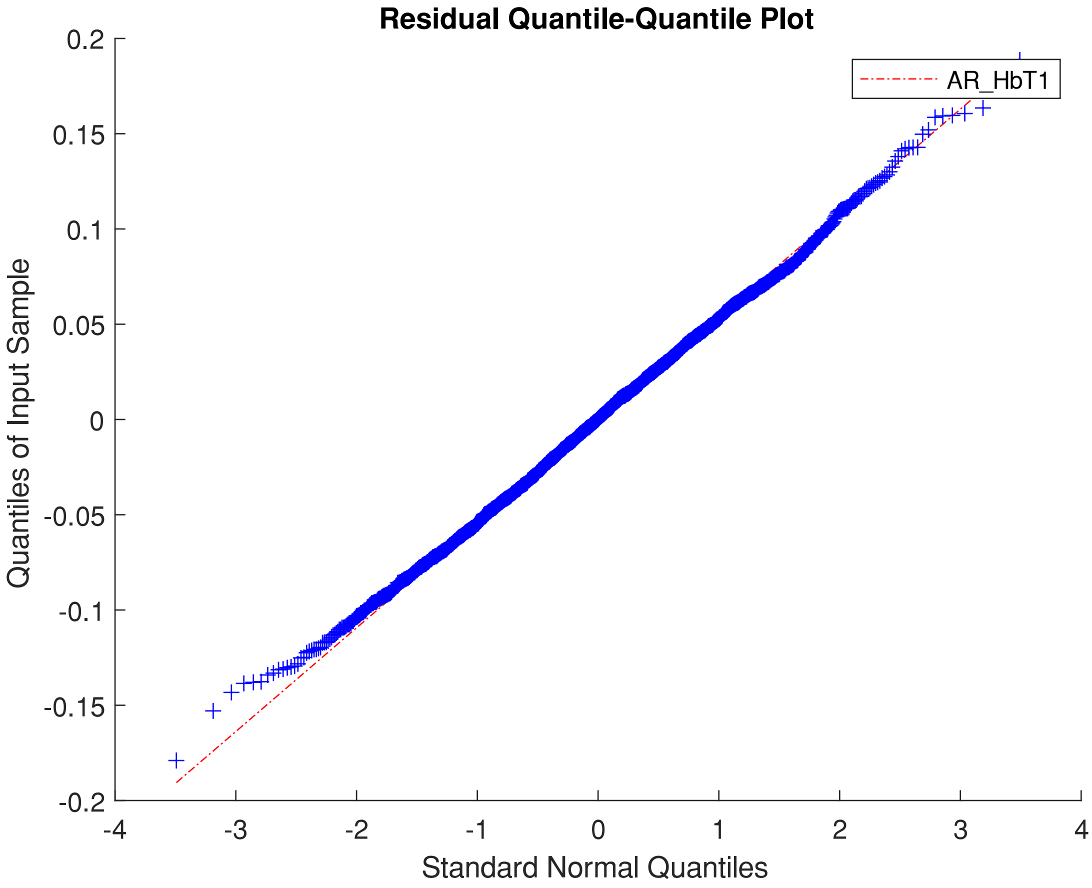

Figure 9.1. Quantile-quantile plot of the residuals of model AR_HbT1.

9.3. Residual Sample Autocorrelation Function

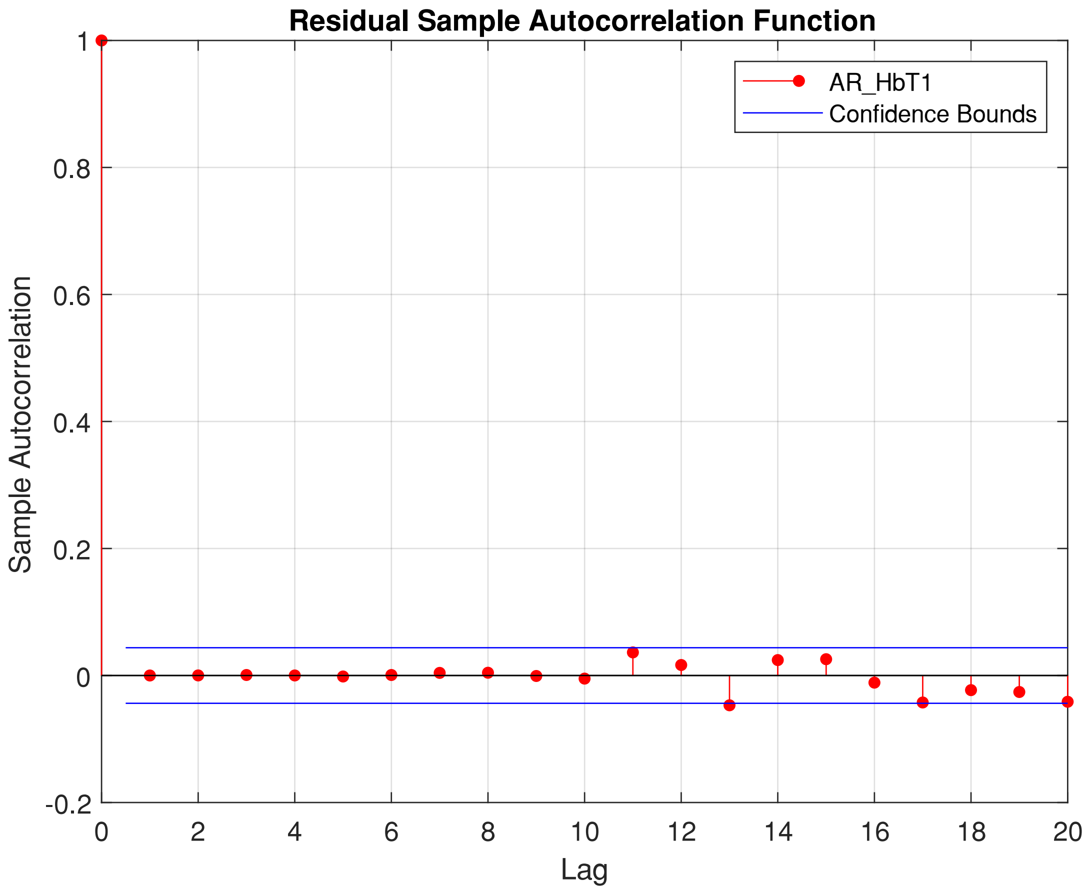

Figure 9.2. Sample autocorrelation function of the residuals of AR_HbT1

9.4. Ljung-Box Q-Test

Null Hypothesis: The first m autocorrelations of the residuals of AR_HbT1 are jointly 0

$$H_0 : \rho_1 = \rho_2 = \ldots = \rho_m = 0$$

$$H_a : \rho_j \neq 0, j \in 1, \ldots, m$$

Table 9.3. Test Parameters

	Lags	DOF	Significance Level
1	10	10	0.025

Table 9.4. Test Results

	Null Rejected	P-Value	Test Statistic	Critical Value
1	false	1	0.13467	20.4832

9.5. Sample Autocorrelation Function

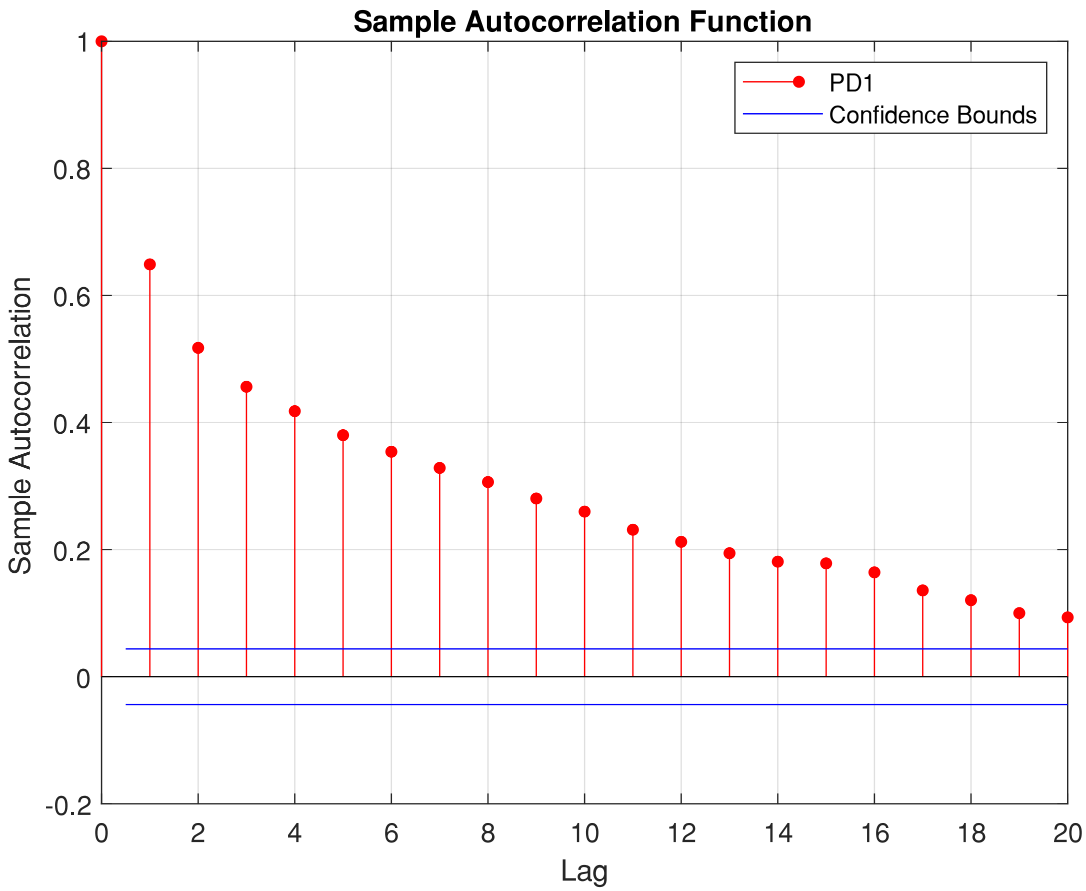

Figure 9.3. Sample autocorrelation function of PD1

9.6. Sample Partial Autocorrelation Function

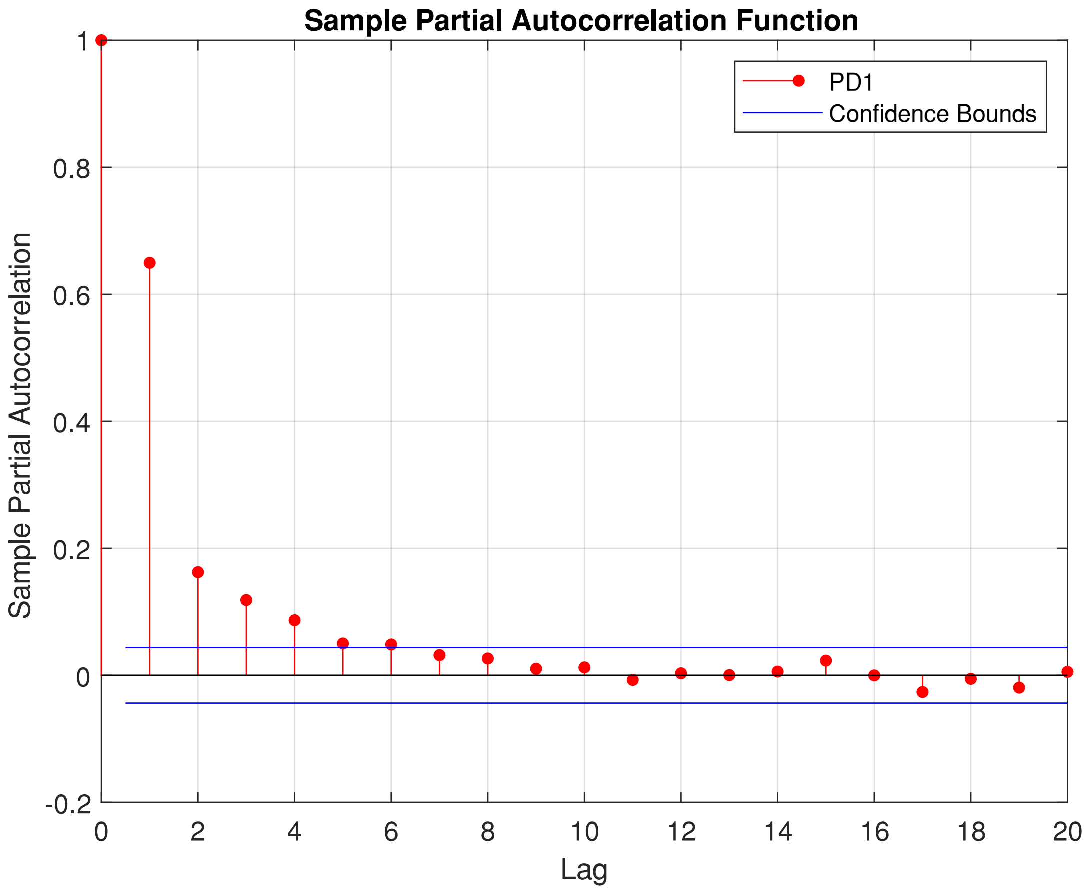

Figure 9.4. Sample partial autocorrelation function of PD1

9.7. Augmented Dickey-Fuller Test

Null Hypothesis: PD1 contains a unit root

$$y_t = c + \delta t + \phi y_{t-1} + \beta_1 \Delta y_{t-1} + \dots + \beta_p \Delta y_{t-p} + \varepsilon_t$$

$$H_0 : \phi = 1$$

$$H_a : \phi < 1$$

Table 9.5. Test Parameters

	Lags	Model	Test Statistic	Significance Level
1	0	AR	t1	0.025
2	1	AR	t1	0.025

Table 9.6. Test Results

	Null Rejected	P-Value	Test Statistic	Critical Value
1	true	0.0049631	-2.8071	-2.2297
2	false	0.035388	-2.0894	-2.2297

10.ARIMA(7,0,0) Model (Gaussian Distribution) (AR_PD1)

Autoregressive model of time series PD1 with the following equation:

$$(1 - \phi_1 L - \ldots - \phi_7 L^7) y_t = c + \varepsilon_t$$

10.1. Model Estimation

Table 10.1. Estimation Results

Parameter	Value	Standard Error	t Statistic	P-Value
Constant	0.14592	0.015835	9.2153	3.1031e-20
AR{1}	0.50915	0.0115	44.2736	0
AR{2}	0.080443	0.016879	4.7657	1.882e-06
AR{3}	0.06344	0.023377	2.7137	0.0066529
AR{4}	0.055222	0.026223	2.1059	0.035216
AR{5}	0.022734	0.030886	0.73605	0.4617
AR{6}	0.033137	0.03801	0.8718	0.38332
AR{7}	0.032287	0.030999	1.0416	0.29762
Variance	0.0063596	8.9936e-05	70.7127	0

Table 10.2. Goodness of Fit

AIC	-4619.3801
BIC	-4568.6103

10.2. Residual Quantile-Quantile plot

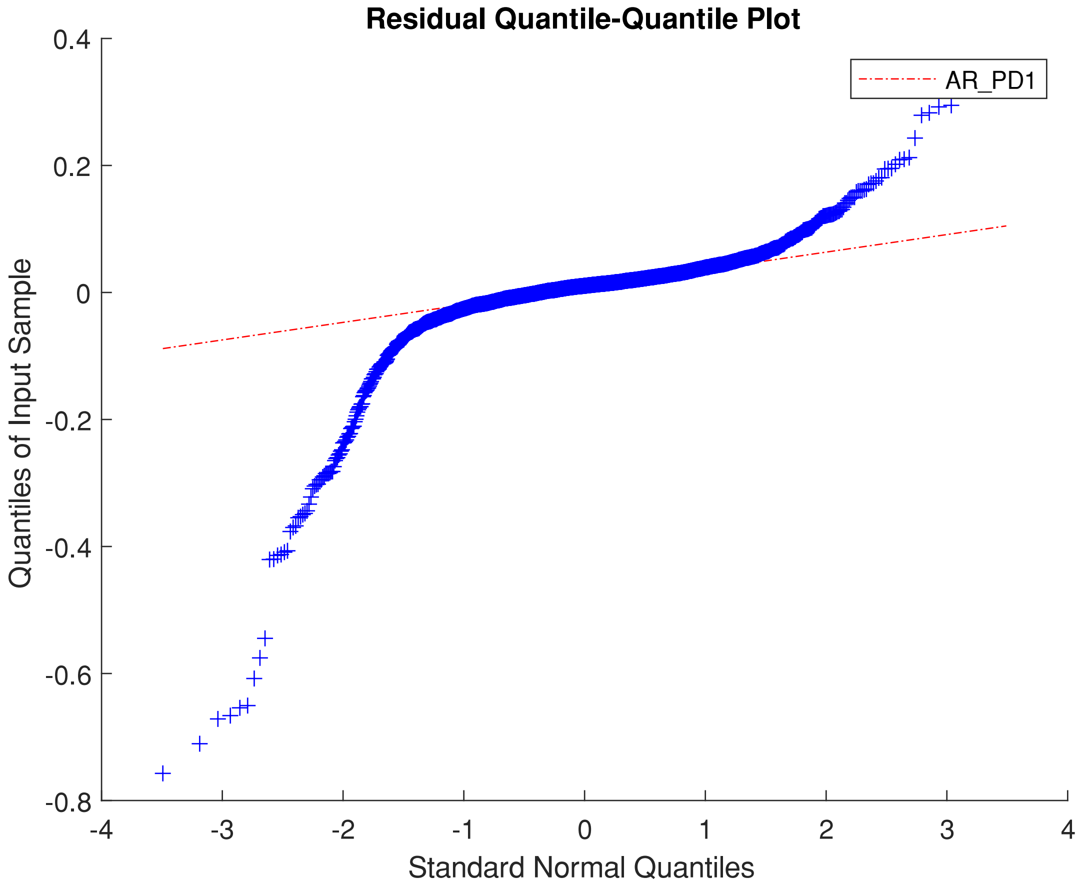

Figure 10.1. Quantile-quantile plot of the residuals of model AR_PD1.

10.3. Residual Sample Autocorrelation Function

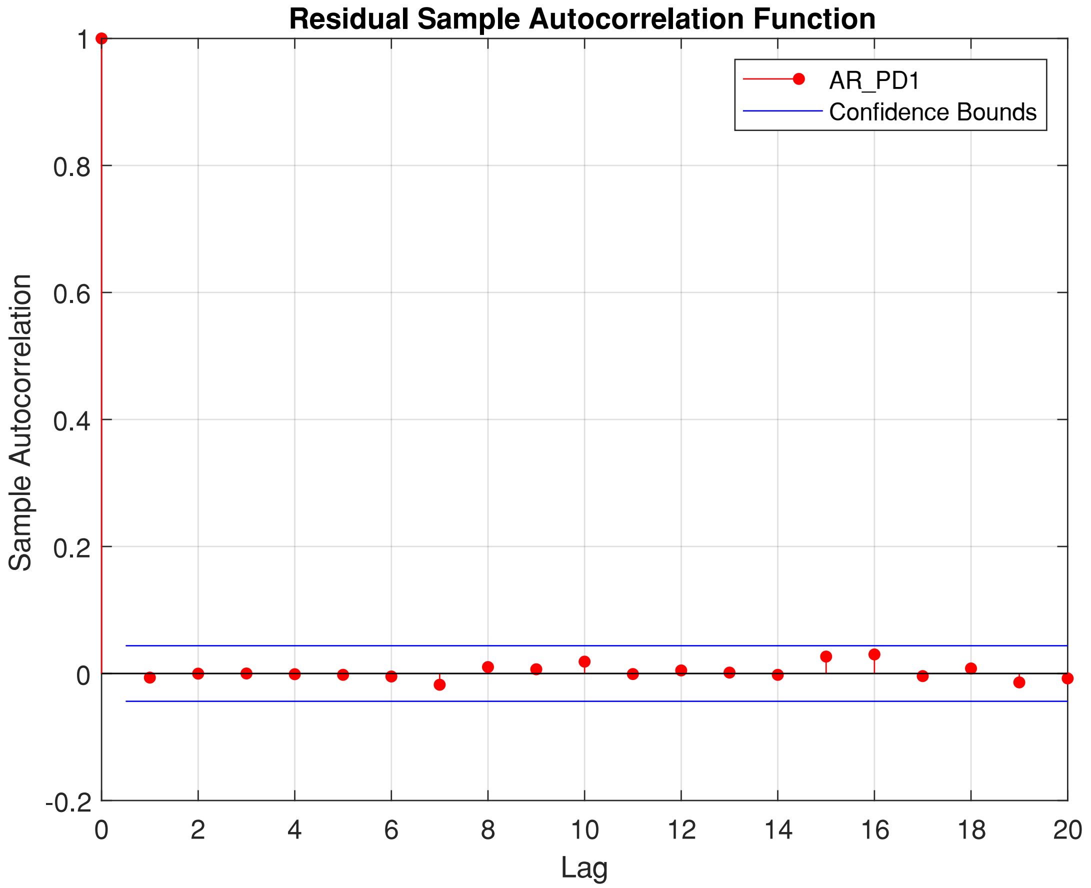

Figure 10.2. Sample autocorrelation function of the residuals of AR_PD1

10.4. Ljung-Box Q-Test

Null Hypothesis: The first m autocorrelations of the residuals of AR_PD1 are jointly 0

$$H_0 : \rho_1 = \rho_2 = \ldots = \rho_m = 0$$

$$H_a : \rho_j \neq 0, j \in 1, \ldots, m$$

Table 10.3. Test Parameters

	Lags	DOF	Significance Level
1	10	10	0.025

Table 10.4. Test Results

	Null Rejected	P-Value	Test Statistic	Critical Value
1	false	0.9974	1.8447	20.4832

3. GCtest 'blockwise'

H0	Decision	Distribution	Statistic	PValue	CriticalValue
"Exclude lagged tDCS in HbT,PD equations"	"Reject H0"	"F(38,2001)"	2.2517	2.1601e-05	1.4109

H0	Decision	Distribution	Statistic	PValue	CriticalValue
"Exclude lagged tDCS in HbT equations"	"Reject H0"	"F(19,2001)"	1.7127	0.028037	1.5917

H0	Decision	Distribution	Statistic	PValue	CriticalValue
"Exclude lagged tDCS in PD equations"	"Reject H0"	"F(19,2001)"	2.7897	5.4982e-05	1.5917
